# Study on the efficacy of IFN-γ- and sPD-1-overexpressing BMSCs in enhancing immune effects for the treatment of lung adenocarcinoma

**DOI:** 10.3389/fimmu.2025.1554467

**Published:** 2025-03-13

**Authors:** Yahui Xie, Zhen Lv, Yubin Wang, Jin Ma, Xingmin Wei, Guisen Zheng, Jianjun Wu

**Affiliations:** ^1^ School of Public Health, Gansu University of Traditional Chinese Medicine, Lanzhou, China; ^2^ Center for Laboratory Medicine, The Second Hospital of Lanzhou University, Lanzhou, China

**Keywords:** IFN-γ, sPD-1, BMSCs, lung adenocarcinoma, immune suppression

## Abstract

**Background:**

Soluble programmed cell death receptor-1 (sPD-1) blocks the PD-1/PD-L1 pathway, reverses tumor immune suppression, and inhibits tumor growth. However, clinical applications are limited by its poor tissue distribution and rapid dispersion. Bone marrow-derived mesenchymal stem cells (BMSCs) are favorable carriers for tumor immunotherapy due to their capacity for external gene introduction and targeted tumor homing. However, they may inadvertently promote tumor growth. Interferon-gamma (IFN-γ) inhibits BMSC-mediated tumor growth and stimulates antigen-presenting cells to activate T lymphocytes. This study utilizes BMSCs transfected with IFN-γ as carriers for sPD-1, enabling the targeted homing of sPD-1 to tumor tissues, thereby enhancing the efficacy and sustained stability of immunotherapy.

**Methods:**

stable IFN-γ- and sPD-1-overexpressing BMSCs were successfully constructed by lentiviral transfection. A non-contact co-culture system was established with Lewis and A549 lung adenocarcinoma cells to observe changes in the lung cancer cells after co-culture, using assays including cell migration and invasion experiments, as well as cellular senescence detection. Additionally, a subcutaneous lung adenocarcinoma model was established in C57BL/6J mice for intervention studies. Tumor volume, cellular apoptosis in tumor tissue (assessed by TUNEL assay), peripheral Treg cells (analyzed by flow cytometry), and histopathological markers (evaluated by HE staining and immunohistochemistry) were analyzed. The expression levels of BAX, BCL-2, AKT, PI3K, and PD-L1 were assessed by quantitative PCR and Western Blot.

**Results:**

IFN-γ- and sPD-1-overexpressing BMSCs exhibited high bioactivity and genetic stability, inhibiting lung adenocarcinoma cell proliferation, accelerating cellular senescence, and reducing migration and invasion. Furthermore, they upregulate Bax expression, downregulate Bcl-2, and promote apoptosis. Additionally, these cells alleviate inflammatory damage in lung tissue of tumor-bearing mice, lower Treg cell levels to inhibit tumor immune evasion, and reduce the expression of PI3K/AKT and PD-L1.

**Conclusion:**

IFN-γ- and sPD-1-overexpressing BMSCs effectively inhibit lung adenocarcinoma cell growth and tumor progression. The primary mechanisms include suppression of cancer cell growth, migration, and invasion; promotion of apoptosis and senescence in cancer cells; modulation of Treg cells; and inhibition of the PI3K/AKT signaling pathway and PD-1/PD-L1 pathways.

## 1 Introduction

Advances in tumor immunology, cellular biology, and molecular biology have driven rapid progress in tumor immunotherapy, both in laboratory research and clinical applications. The primary mechanism of tumor immunotherapy involves activating the host immune response to enhance anti-tumor immunity and suppress tumor cells (1). During tumor progression, programmed cell death receptor-1 (PD-1) binds to its ligand PD-L1, mediating a negative immune co-stimulatory signal. This interaction reduces the secretion of the T-cell immune-stimulatory cytokine IFN-γ while increasing the secretion of the immunosuppressive cytokine IL-10. Consequently, T-cell proliferation and activation are inhibited, enabling tumor cells to evade immune surveillance and continue proliferating (2). Soluble PD-1 (sPD-1) blocks the PD-1/PD-L1 signaling pathway, reverses host immunosuppression. This reactivates the endogenous T lymphocytes, promoting their proliferation and restoring immune surveillance functions, thereby inhibiting tumor cell growth (3). However, clinical applications face challenges due to poor tumor-targeting efficiency after injection (4), rapid systemic diffusion, and associated adverse reactions (5). BMSCs are promising vectors for tumor immunotherapy due to their ability to incorporate exogenous genes and home to tumor sites (6). Their tumor-homing capability enhances the precision of immunotherapy, making BMSCs ideal carriers for anti-tumor research and clinical applications. However, BMSCs may cross-communicate and inadvertently promote tumor growth (7), necessitating solutions to mitigate this effect. Interferon-gamma (IFN-γ) is a critical cytokine that modulates the tumor-promoting or tumor-suppressing behavior of BMSCs in the cellular matrix (8–10). IFN-γ not only inhibits BMSC-mediated pro-tumor crosstalk but also stimulates antigen-presenting cells to reactivate T lymphocytes, thereby enhancing cellular immunity (11, 12). This dual regulatory role makes IFN-γ a critical modulator in anti-tumor therapy (13). In this study, IFN-γ-transfected BMSCs serve as targeted carriers for sPD-1, enabling its delivery to tumor tissues and enhancing the specificity and sustained efficacy of immunotherapy. A lentiviral vector was used to construct IFN-γ and sPD-1 plasmids, which were then transfected into murine BMSCs. The genetic characteristics and biological activity of transfected BMSCs were validated, and their effects on lung adenocarcinoma cells and tumor-bearing mice were examined. This study investigates the stability, efficacy, and mechanisms of IFN-γ- and sPD-1-overexpressing BMSCs in targeted immunotherapy for lung adenocarcinoma, providing novel methods and insights for clinical tumor prevention and treatment.

## 2 Materials and methods

### 2.1 Experimental animals and cell lines

A total of 70 specific-pathogen-free (SPF) grade C57BL/6J male mice, aged 5-6 weeks and weighing 160-180 g, were obtained from Beijing SPF Biotechnology Co., Ltd. (Beijing, China). The animals were housed in the SPF-grade Experiment Animal Center of Gansu University of Chinese Medicine under controlled temperature (20–25°C), humidity (50% ± 5%), natural lighting, and free access to water. All animal experiments were approved by the Animal Research Ethics Committee of Gansu University of Chinese Medicine (SY2023-623).

The human lung adenocarcinoma cell line A549 and mouse lung adenocarcinoma cell line Lewis were obtained from the Cell Bank of the Chinese Academy of Sciences (Shanghai, China) and maintained in our laboratory under standard culture conditions.

### 2.2 Extraction, culture, and identification of BMSCs

Ten mice were used to isolate femurs and tibias under sterile conditions. Bone marrow was collected by flushing with MEMα medium using a sterile 2 mL syringe. The collected bone marrow was homogenized and filtered through a 200-mesh cell strainer. The filtrate was centrifuged to collect the cell pellet. Mouse lymphocyte separation fluid was added, followed by centrifugation, and the intermediate buffy coat cells were collected, diluted in PBS, centrifuged again, and the supernatant discarded. The cell pellet was resuspended in complete mouse BMSCs culture medium, seeded into pretreated culture dishes, and incubated under controlled conditions.

Identification was performed using flow cytometry. Approximately 1 × 10^6^ third-generation BMSCs were labeled with fluorescence-tagged antibodies, including CD44-FITC, CD105-PE, CD34-PE, CD45-FITC, and CD11b-PE. Samples were incubated at 4°C in the dark for 30 min. After incubation, 500μL of PBS containing fetal bovine serum was added to each tube, mixed, and centrifuged at 800 × g for 10 min. The supernatant was discarded, and 400μL PBS was added to resuspend the cells for detection.

### 2.3 Construction of mouse IFN-γ and sPD-1 viral plasmids

PCR primers were designed to include the target genes for mouse IFN-γ and sPD-1 with BP recombination, and the IRES-GFP (green fluorescent protein) sequence was amplified from the pIREs-GFP plasmid as a template to construct the target gene expression plasmid vector. Lentiviral shuttle plasmids and accessory packaging element vectors were co-transfected into HEK293T cells. After 48 hours of culture in complete medium, the virus-containing supernatant was collected, concentrated to obtain high-titer lentivirus, and stored at -80°C for later use.

### 2.4 Detection of biological activity and genetic stability of IFN-γ- and sPD-1-overexpressing BMSCs

The required viral volume was calculated based on a specific multiplicity of infection (MOI) and cell number, mixed with the prepared culture medium, and incubated overnight in a 37°C, 5% CO_2_ incubator. The medium was replaced and cells were cultured overnight, then observed under a fluorescence microscope. Puromycin selection was used to screen and purify the IFN-γ- and sPD-1-overexpressing BMSCs. The mRNA levels of IFN-γ and sPD-1 in BMSCs were verified by qPCR, telomerase activity was measured following the TERT kit protocol to assess genetic stability, and cell growth was observed microscopically.

### 2.5 qPCR

Cells were collected in centrifuge tubes, chloroform (0.2 mL) was added, and the samples were vortexed and centrifuged. Isopropanol (0.5 mL) was added, followed by centrifugation and removal of the supernatant. The pellet was washed with 1mL of 75% ethanol, centrifuged, and the supernatant discarded. One microliter of extracted RNA was measured using a micro-volume spectrophotometer for concentration (ng/µL). DNA was removed, and a reverse transcription reaction system was prepared to synthesize cDNA. The PCR reaction system was set up in RNase-free microtubes, with each template having three technical replicates. A two-step PCR method was used, and the amplification and melting curves of the real-time PCR were confirmed after the reaction (**Table 1**).

**Table 1:** qPCR Primer Sequences.

Gene	Forward Primer Sequence	Reverse Primer Sequence	Product Length (bp)
GAPDH	CCCTTAAGAGGGATGCTGCC	TACGGCCAAATCCGTTCACA	124
IFN-γ	CATGGCTGTTTCTGGCTGTT	TCCTTTTGCCAGTTCCTCCA	138
sPD-1	GGTCGGAGGATCTTATGCTGAACTG	AGAGGTAGATGCCACTGTCATTGC	125
BAX	CCCGAGCTGATCAGAACCAT	TCACTGAGGGGTCTCACCCA	104
BCL-2	AAACCCTCCATCCTGTCCAG	CCCTTTCCTAGACCCAGCAA	133
AKT	CACCTTTATCATCCGCTGCC	CTTCCTGCCTCTTGAGTCCA	131
PI3K	ACCTGGACTTAGAGTGTGCC	TACAGTAGTGGGCTTGGGTG	113
PD-L1	GAATTTCCGTGGATCCAGCC	ACTTCTCTTCCCACTCACGG	119

### 2.6 Western blot

Cells were washed with PBS and lysed in RIPA buffer (containing PMSF and protease inhibitors) on ice for 30 min. After centrifugation at 12,000 × g for 15 min at 4°C, the supernatant was collected, and protein concentration was determined using a BCA assay. Equal amounts of protein were mixed with loading buffer, boiled for 5 min, and separated by SDS-PAGE (10% gel) at 80 V through the stacking gel and 120 V through the resolving gel until the dye front reached the bottom. Proteins were then transferred to a PVDF membrane using a wet transfer system at 100 V for 1-2 h. The membrane was blocked with 5% non-fat milk or BSA in TBST for 1 h at room temperature, followed by incubation with primary antibodies (diluted 1:1000) at 4°C overnight. After washing with TBST, the membrane was incubated with HRP-conjugated secondary antibodies (diluted 1:4000) for 1.5 h at room temperature. The membrane was washed again with TBST, and protein bands were visualized using an ECL substrate and captured using a chemiluminescence imaging system. Quantification of band intensity was performed using ImageJ software. Experimental conditions were optimized as needed.

### 2.7 Establishment of non-contact co-culture system of IFN-γ- and sPD-1-overexpressing BMSCs with Lewis and A549 Cells

Lewis and A549 cells were seeded in the lower chamber of a Transwell co-culture system, with IFN-γ and sPD-1 dual-gene transfected BMSCs seeded in the upper chamber to establish a co-culture system with these lung adenocarcinoma cell lines. Cells were collected and transferred to culture flasks for continued culture.

The experimental groups were as follows: Control: Lung adenocarcinoma cells cultured alone; Vector: Lung adenocarcinoma cells co-cultured with empty vector BMSCs; IFN-γ: Lung adenocarcinoma cells co-cultured with IFN-γ-overexpressing BMSCs; sPD-1: Lung adenocarcinoma cells co-cultured with sPD-1-overexpressing BMSCs; IFN-γ + sPD-1: Lung adenocarcinoma cells co-cultured with IFN-γ- and sPD-1-overexpressing BMSCs.

### 2.8 Detection of cell migration and invasion ability

In a six-well plate, three lines were drawn to divide each well into three sections. Lung adenocarcinoma cells (1.8×10^5^) were seeded into each well, achieving a confluence of 70%. A scratch was made vertically across the bottom of each well along the pre-drawn lines, followed by medium replacement with serum-free medium and subsequent imaging.

Matrigel was diluted 1:8 with PBS and applied to Transwell chambers, incubated at 37°C to solidify. A cell suspension (200 μL) was added to the upper chamber, and serum-containing medium (500 μL) was added to the lower chamber. After 48 hours, cells were washed, fixed, stained with crystal violet, and observed under a microscope.

### 2.9 Senescence-associated β-galactosidase assay

After co-culture, lung adenocarcinoma cells were seeded at a density of 1×10^5^ cells per well in a six-well plate. Following 24 hours, β-galactosidase staining was performed according to the kit instructions to assess senescence levels, with imaging under an inverted microscope.

### 2.10 Establishment of a subcutaneous lung adenocarcinoma tumor-bearing model and therapeutic interventions

C57BL/6J mice (n=60, male, 5-6 weeks) were randomly divided into a normal control group (n=10) and a tumor-bearing group (n=50), with adaptive feeding for one week. Exponentially growing Lewis cells (1×10^7^ cells/mL, 0.1mL per mouse) were injected into the right axilla of each mouse. Tumor formation was confirmed by palpating a 3-5 mm nodule.

Groups (n=10/group) included: Control: Normal group; Model: Tumor model group; Vector: Intervention with empty vector BMSCs; IFN-γ: Intervention with IFN-γ-overexpressing BMSCs; sPD-1: Intervention with sPD-1-overexpressing BMSCs; IFN-γ + sPD-1: Intervention with IFN-γ- and sPD-1-overexpressing BMSCs.

Intervention involved tail vein injection of 2×10^4^ modified BMSCs every three days for a total of four injections. The experiment was terminated when the tumor volume reached the ethical limit (2cm³), after which all mice were euthanized, and samples were collected.

### 2.11 Quantitative analysis of tumor growth dynamics

Longitudinal monitoring of tumor progression was performed at 48-hour intervals using digital calipers. Tumor volume (mm³) was calculated using the standard ellipsoid formula: Volume = 0.5 × (major axis) × (minor axis)². After 12 days of intervention, mice were euthanized, and lung and tumor tissues were dissected for assessment of morphology, texture, color, and weight. The tumor inhibition rate was calculated as follows: inhibition rate = (average tumor weight in model group - average tumor weight in treatment group)/average tumor weight in model group × 100%.

### 2.12 Histopathological evaluation and immunohistochemical staining

Tumor and lung tissues were fixed in 4% paraformaldehyde, dehydrated in an ethanol gradient, permeabilized in xylene, and stained with hematoxylin and eosin. Slides were then dehydrated, mounted, and imaged under a microscope.

For immunohistochemistry, sections were treated with 3% H_2_O_2_ to block endogenous peroxidase, incubated with antibodies at 4°C, stained with DAB solution, counterstained with hematoxylin, and mounted for imaging. Positive cells were analyzed using ImageJ.

### 2.13 Detection of apoptotic cells by TUNEL assay

The TUNEL assay was performed according to the kit instructions, and apoptotic cells were observed and photographed under a fluorescence microscope.

### 2.14 Flow cytometric quantification of Treg cells

Peripheral blood (100 μL per tube) was stained with CD4 and CD25 for surface marker detection. Following the addition of 2mL of 1× FCM Lysing Solution, samples were vortexed thoroughly and centrifuged. The supernatant was discarded, and 2mL of PBS was added, mixed well, and centrifuged for 5 min. After removing the supernatant, cells were fixed by adding 1mL of Fixation/Permeabilization working solution, vortexed to mix, and stained with an appropriate amount of FoxP3 antibody according to the kit instructions. The mixture was incubated at room temperature in the dark for at least 30 min. Following this, 2 mL of 1× Permeabilization Buffer was added and centrifuged. Finally, the cells were resuspended in 500 μL of PBS for flow cytometry analysis.

### 2.15 Statistical analysis

Statistical analysis and plotting were conducted using GraphPad Prism 8.0. All experiments were independently repeated three times, and data were expressed as mean ± standard deviation ( $\bar{\text{x}}$ ± s). Intergroup comparisons were conducted using one-way analysis of variance (ANOVA) followed by Dunnett’s *post-hoc* test for multiple comparisons. A *P* -value of <0.05 was considered statistically significant.

## 3 Results

### 3.1 IFN-γ- and sPD-1-overexpressing BMSCs have high biological activity and genetic stability

**Figure 1:**
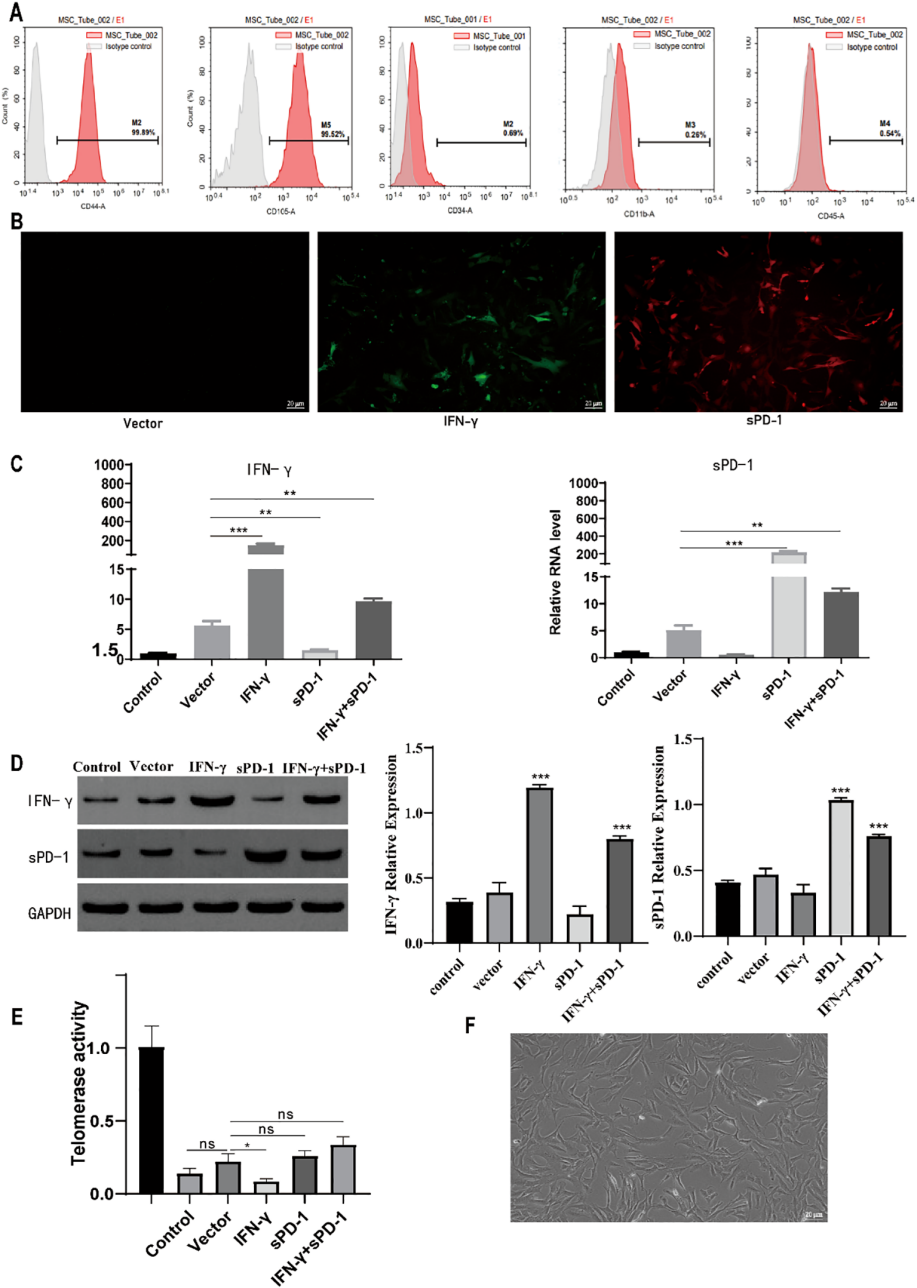
Identification of BMSCs and results of IFN-γ- and sPD-1-overexpressing BMSCs. **(A)** Identification of surface markers in BMSCs; **(B)** Immunofluorescence results of IFN-γ- and sPD-1-overexpressing BMSCs; **(C)** qPCR results of IFN-γ- and sPD-1-overexpressing BMSCs; **(D)** WB results of IFN-γ- and sPD-1-overexpressing BMSCs; **(E)** Telomerase activity of IFN-γ- and sPD-1-overexpressing BMSCs in mice; **(F)** Morphology of BMSCs after overexpression of IFN-γ and sPD-1. * *P <*0.05;**P<0.01;*** *P <*0.001 vs Control group. ns, not significant.

Flow cytometric analysis revealed that the isolated BMSCs exhibited high purity, with surface marker expression of CD44 (99.82% ± 0.52%) and CD105 (99.52% ± 1.71%), both exceeding the threshold of 95% for BMSC identification. The expression of negative markers CD34, CD11b, and CD45 was 0.69% ± 0.25%, 0.26% ± 0.13%, and 0.54% ± 0.29%, respectively, all within the acceptable range for BMSC identification (**Figure 1A**).

At an MOI of 20, distinct red and green fluorescence signals were observed, with specific fluorescence and no apparent nonspecific signals. IFN-γ emits green fluorescence, while sPD-1 emits red fluorescence (**Figure 1B**). qPCR and WB analysis demonstrated that sPD-1 mRNA and protein levels were significantly upregulated in the sPD-1 and IFN-γ+sPD-1 groups compared to the control group (P < 0.05). Similarly, IFN-γ mRNA and protein expression was markedly elevated in the IFN-γ and IFN-γ+sPD-1 groups (*P* < 0.05) (**Figures 1C, D**).

Telomerase activity, assessed using the TERT assay, showed no significant difference between the control and empty vector groups (*P* > 0.05). However, the IFN-γ group exhibited a significant reduction in telomerase activity compared to the empty vector group (*P* < 0.05). In contrast, the sPD-1 and IFN-γ+sPD-1 groups showed comparable telomerase activity to the empty vector group (*P* > 0.05) (**Figure 1E**). Additionally, the morphology of the transfected BMSCs appeared normal, fibroblast-like in shape, with uniform size and good growth status (**Figure 1F**).

### 3.2 Suppression of lung adenocarcinoma cell proliferation and acceleration of cellular senescence by >IFN-γ- and sPD-1-overexpressing BMSCs

**Figure 2:**
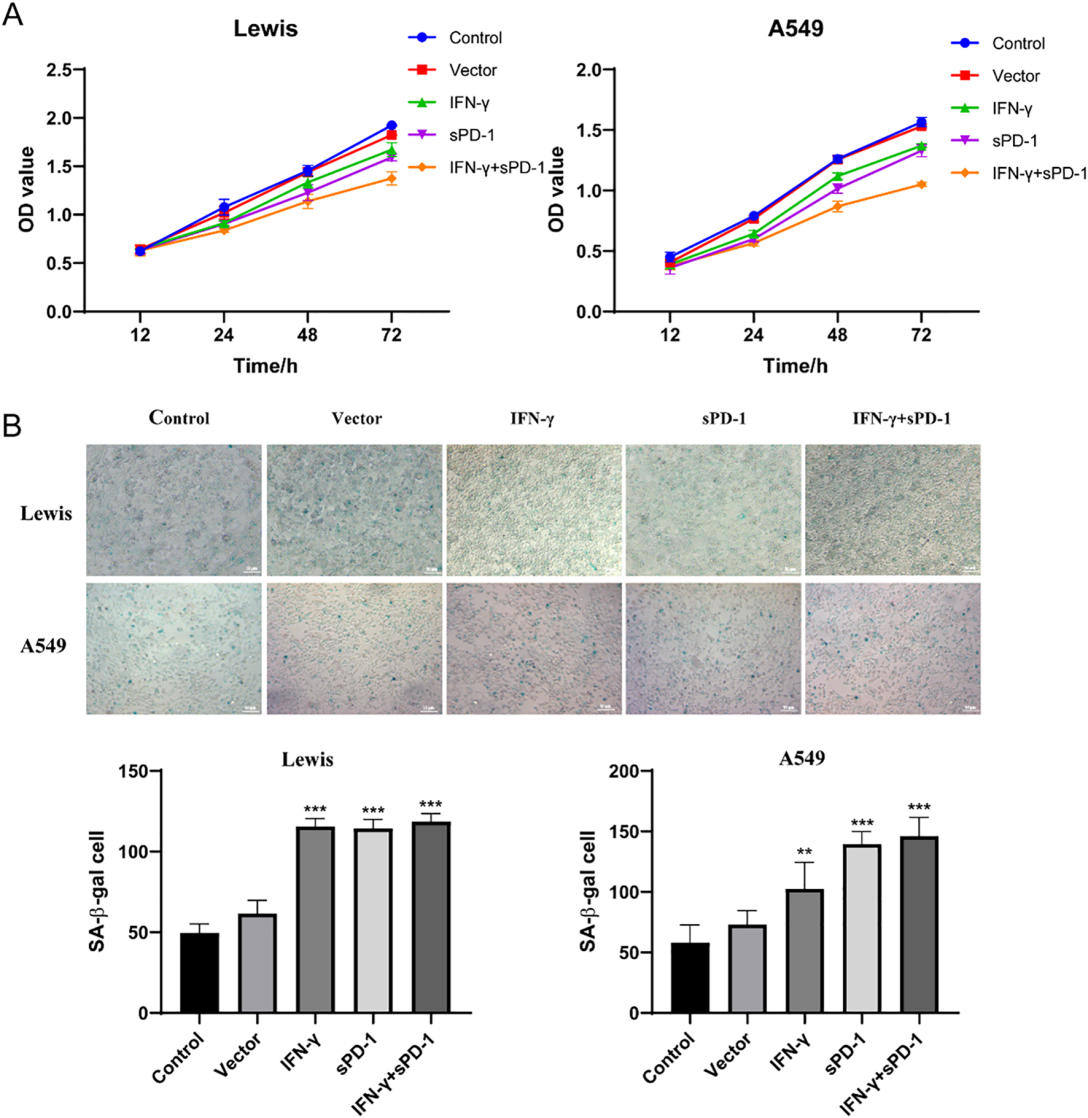
Effects of IFN-γ- and sPD-1-overexpressing BMSCs on lung adenocarcinoma cell proliferation and senescence. **(A)** CCK8 assay showing the proliferation of lung adenocarcinoma cells after co-culture with IFN-γ- and sPD-1-overexpressing BMSCs. **(B)** β-galactosidase assay showing the effect of IFN-γ- and sPD-1-overexpressing BMSCs on the senescence level of lung adenocarcinoma cells after co-culture.**p<0.01 and ***p<0.001 vs Control group.

Cell proliferation assays using the CCK-8 kit revealed that co-culture with IFN-γ- or sPD-1-overexpressing BMSCs significantly suppressed the proliferation of Lewis and A549 cells compared to the control group. The IFN-γ+sPD-1 group exhibited the most pronounced anti-proliferative effect, with optical density (OD) values significantly lower than those of the single-gene-transfected groups (*P* < 0.05) (**Figure 2A**).

Senescence-associated β-galactosidase (SA-β-Gal) staining demonstrated that co-culture with IFN-γ, sPD-1, or IFN-γ+sPD-1 overexpressing BMSCs significantly increased the proportion of senescent cells in both Lewis and A549 cells compared to the control group (*P* < 0.001). This indicated that IFN-γ and sPD-1 overexpression in BMSCs potently induced cellular senescence in lung adenocarcinoma cells (**Figure 2B**).

### 3.3 IFN-γ- and sPD-1-overexpressing BMSCs reduces the migration and invasion of lung adenocarcinoma cells

**Figure 3:**
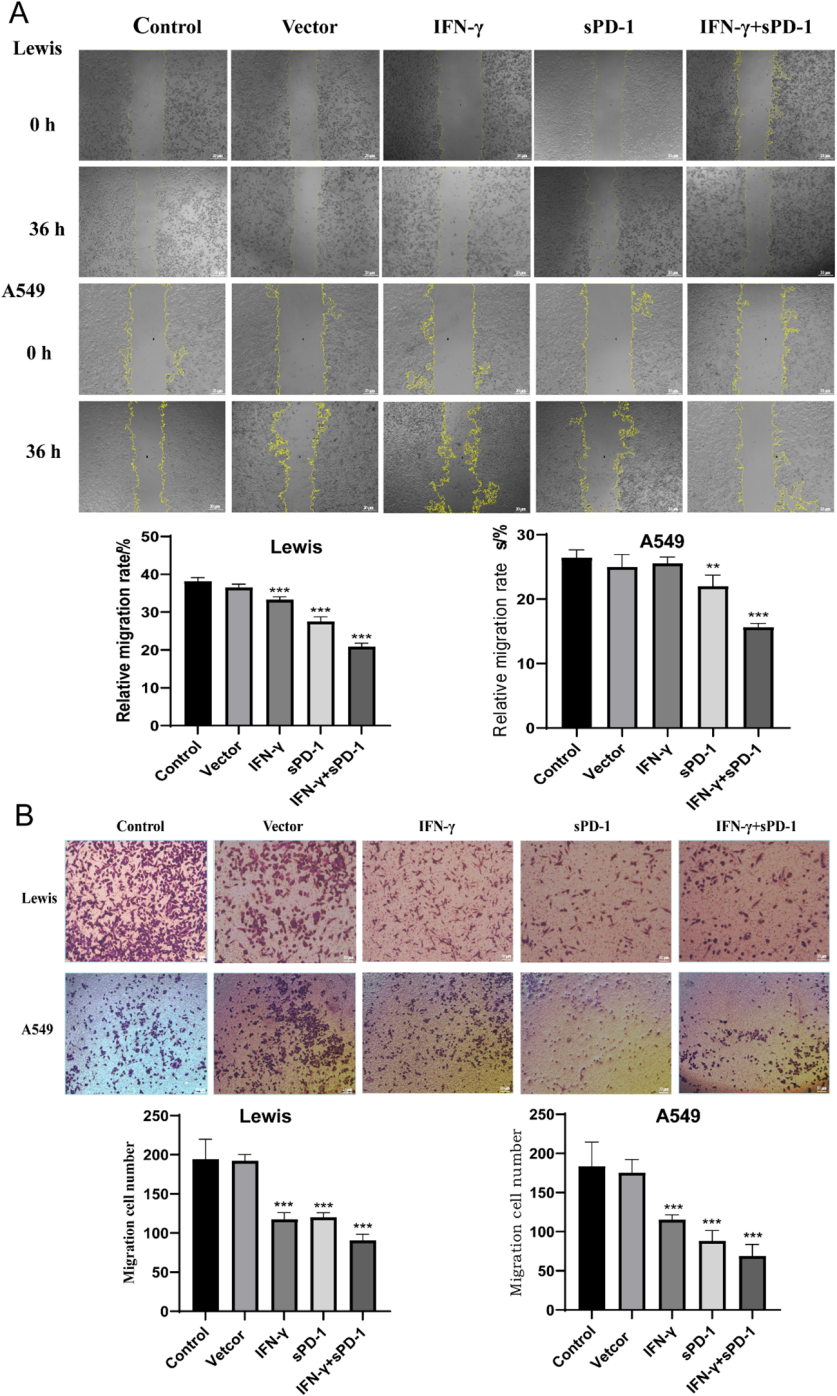
Effects of IFN-γ- and sPD-1-overexpressing BMSCs on the migration and invasion of lung adenocarcinoma cells. **(A)** Scratch assay detecting the migration ability of lung adenocarcinoma cells. **(B)** Transwell cell invasion assay detecting the invasion ability of lung adenocarcinoma cells. **P<0.01 and ***p<0.001 vs Control group.

Scratch wound assays demonstrated that compared to the control group, the migration rate of Lewis cells was significantly lower in the BMSC intervention groups overexpressing IFN-γ, sPD-1, and IFN-γ + sPD-1 (*P* < 0.01). Similarly, in A549 cells, the migration rate was significantly reduced in the BMSC intervention groups overexpressing sPD-1 and IFN-γ + sPD-1 (*P* < 0.001). Notably, the IFN-γ+sPD-1 group displayed the lowest migration rate, significantly outperforming the single-gene-transfected groups (*P* < 0.001 for Lewis cells; *P* < 0.01 for A549 cells) (**Figure 3A**).

Transwell invasion assays further confirmed that IFN-γ- and sPD-1-overexpressing BMSCs significantly inhibited the invasive capacity of Lewis and A549 cells. After 48 hours, the number of invading cells was significantly reduced in all treatment groups compared to the control group (*P* < 0.001) (**Figure 3B**).

### 3.4 Tumor growth dynamics and apoptosis in tumor-bearing mice

**Figure 4:**
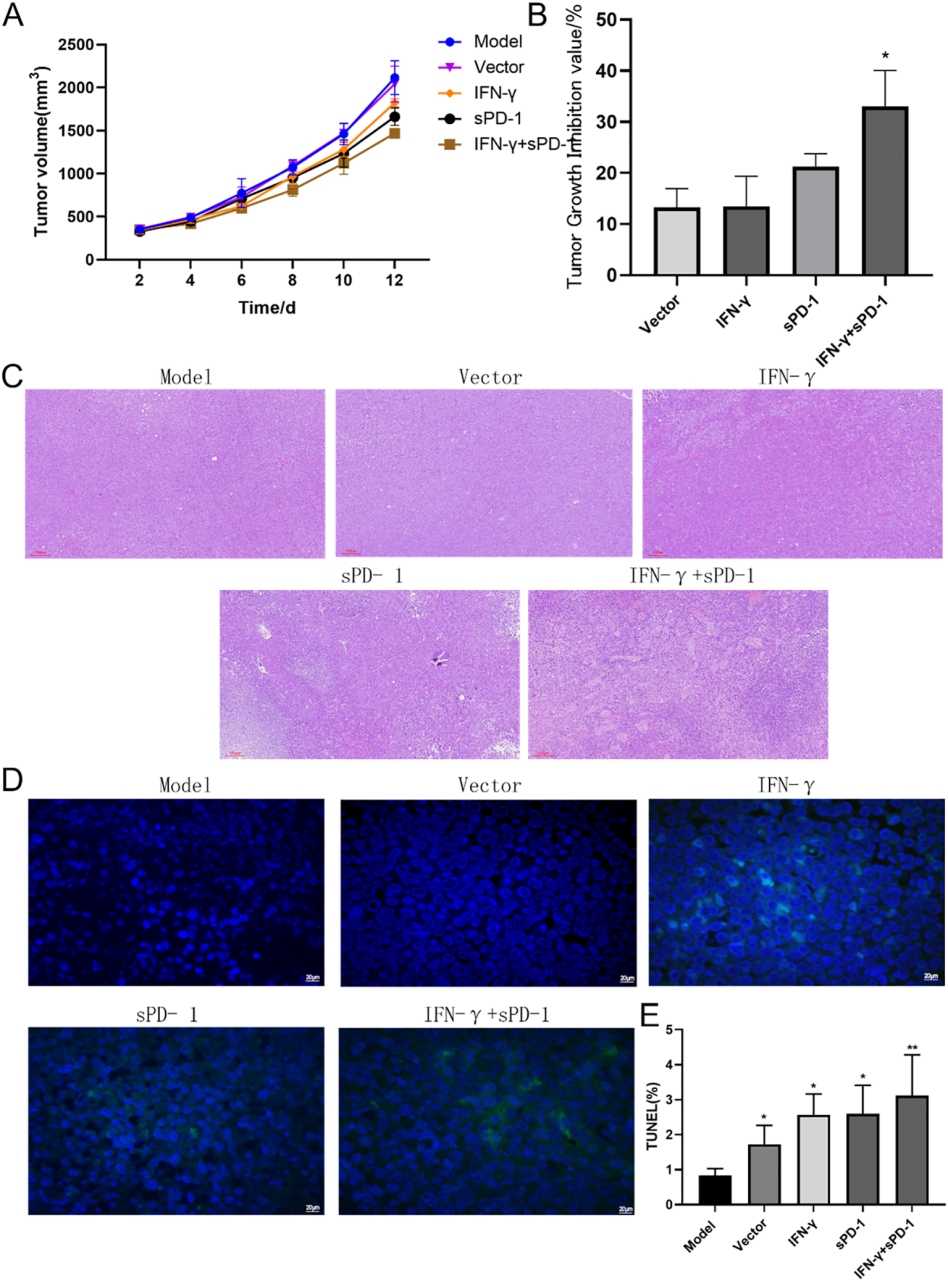
Tumor growth, histological observation, and cell apoptosis in mice. **(A)**:Tumor volume growth curve in mice; **(B)** Tumor inhibition rate analysis; **(C)** Histological observation of tumor tissues in mice; **(D)** TUNEL staining marking primary apoptotic tumor cells; **(E)** ImageJ analysis of tumor cell apoptosis levels in each group. * *P*<0.05 and **p<0.01 vs Model group.

The tumor volume growth curves showed that the slope of the model group was steeper, indicating faster tumor growth (**Figure 4A**). The mice in the IFN-γ + sPD-1 BMSC intervention group exhibited the slowest tumor growth. When calculating the tumor inhibition rate, the results demonstrated that the IFN-γ + sPD-1 group had the highest inhibition rate, and the difference between the IFN-γ + sPD-1-BMSC intervention group and the vetcor group was statistically significant (**Figure 4B**).

Histopathological analysis of H&E-stained tumor sections revealed that in the model group, the tumor cells had clear contours, were densely packed, and were distributed closely, with frequent atypical nuclei and sparse lymphocyte infiltration in the surrounding connective tissue. In contrast, after intervention with IFN-γ + sPD-1 BMSCs, the tumors showed areas of necrosis, and lymphocyte infiltration in the surrounding connective tissue was more pronounced (**Figure 4C**).

TUNEL staining, which marks apoptotic tumor cells, revealed large areas of apoptosis in the IFN-γ + sPD-1 BMSC intervention group (**Figure 4D**), with a significantly higher apoptotic area compared to the model group (*P* < 0.01). Apoptosis rates in other intervention groups were also higher than in the model group (*P* < 0.01, *P* < 0.05) (**Figure 4E**).

### 3.5 IFN-γ- and sPD-1-overexpressing BMSCs upregulates Bax expression and downregulates Bcl-2 expression

**Figure 5:**
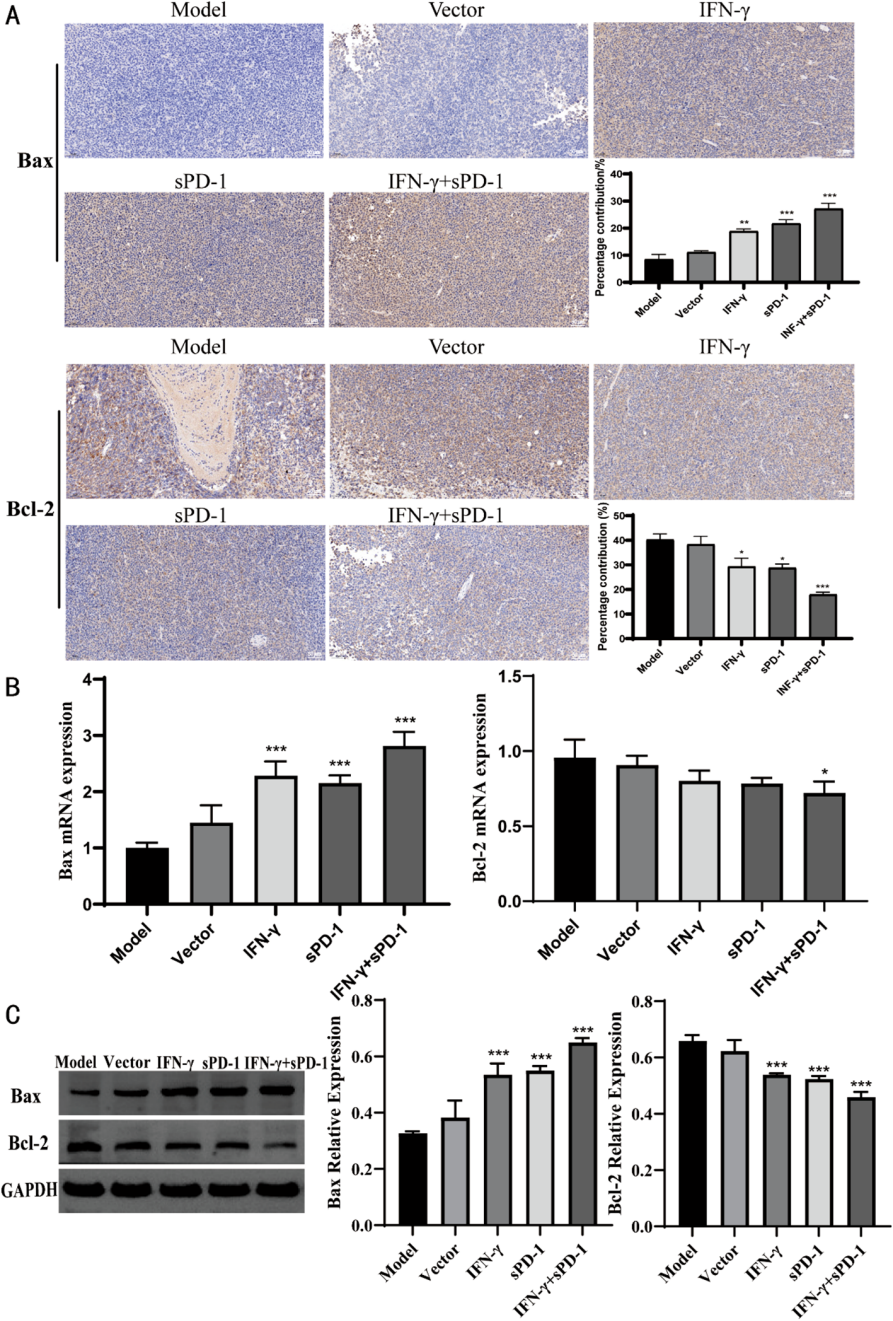
Expression of Bax and Bcl-2 in tumor tissue of each group. **(A)** Immunohistochemical results of Bax and Bcl-2 in each group; **(B)** qPCR results of Bax and Bcl-2 in each group; **(C)** WB results of Bax and Bcl-2 in each group.* *P*<0.05, **p<0.01 and ***p<0.001 vs Model group.

Immunohistochemical staining was performed on tumor tissues from mice to determine the expression of apoptosis-related proteins. The results showed that, compared to the model group, the relative positive expression of Bax protein was significantly higher in the IFN-γ group, the sPD-1 group, and the sPD-1 + IFN-γ BMSC group (*P* < 0.01, *P* < 0.001). Conversely, the relative positive expression of Bcl-2 was significantly lower in these groups (*P* < 0.01, *P* < 0.001) (**Figure 5A**). To further verify the effect of sPD-1 + IFN-γ BMSCs on the expression of apoptosis-related genes in tumor tissues, RT-qPCR and WB was used to measure the mRNA and protein levels of Bax and Bcl-2 in the tumor tissues of mice from each group. The results showed that the mRNA and protein expression levels of Bax were higher in all intervention groups compared to the model group. For Bcl-2 mRNA and protein expression, the sPD-1 + IFN-γ intervention group exhibited lower expression levels than the model group, with statistical significance (*P* < 0.05, *P* < 0.001) (**Figures 5B, C**).

### 3.6 IFN-γ- and sPD-1-overexpressing BMSCs alleviates inflammatory injury in the lung tissue of tumor-bearing mice, reduces Treg levels, and inhibits tumor immune evasion

**Figure 6:**
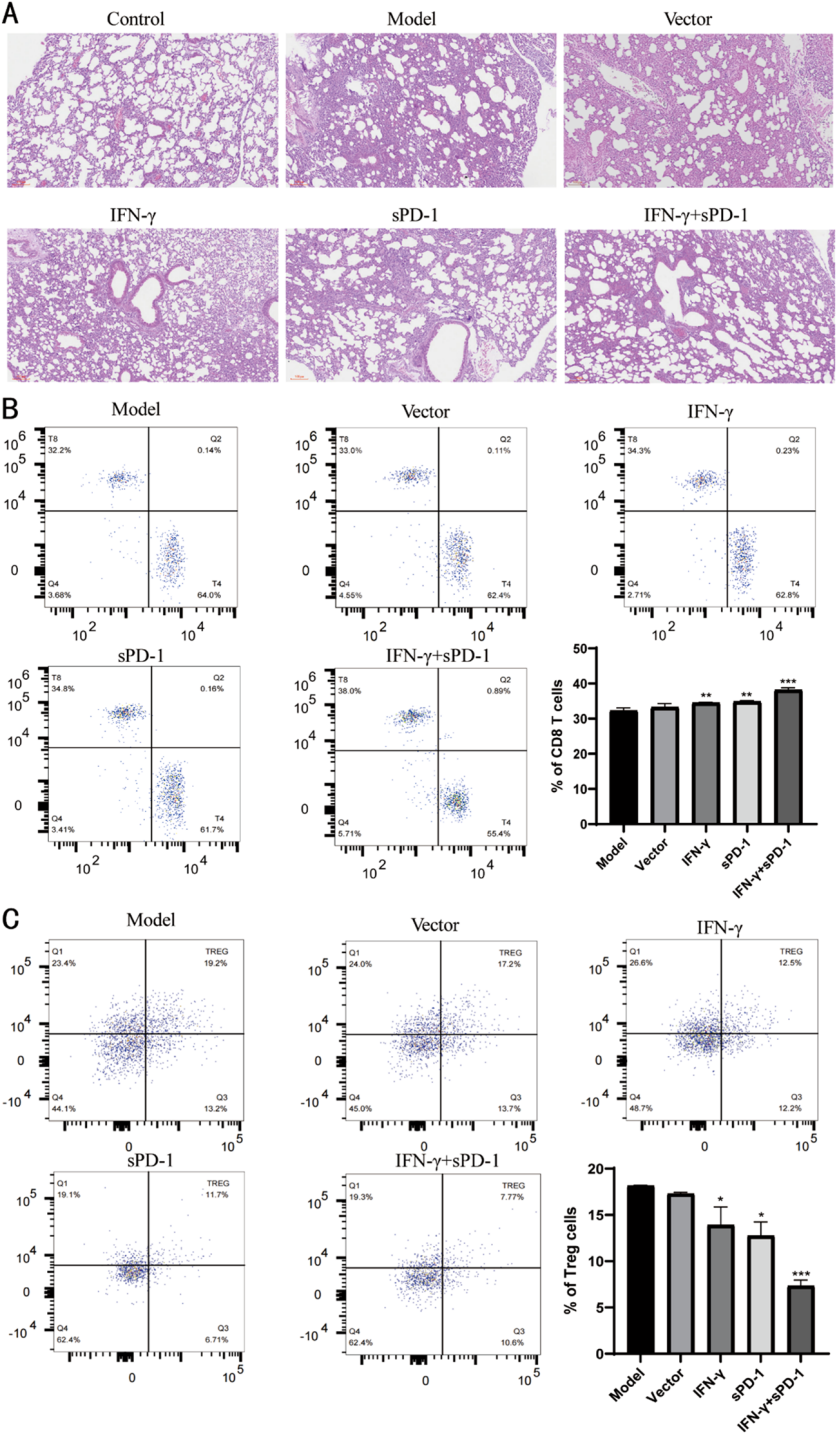
Pathological observation of tumor tissue in mice and distribution of Treg cells in peripheral blood. **(A)** Pathological observation of tumor tissue in mice; **(B)** Distribution of CD8 T cells in peripheral blood of mice and proportion of each group; **(C)** Distribution of Treg cells in peripheral blood of mice and proportion of each group.* *P*<0.05, **p<0.01 and *** *p*<0.001 vs Model group.

Histopathological examination of lung tissues revealed that the control group exhibited normal alveolar architecture, while the model group showed severe pulmonary interstitial edema, alveolar septal thickening, and extensive inflammatory cell infiltration. The IFN-γ+sPD-1 group displayed significantly less tissue damage compared to the model group (**Figure 6A**).

The levels of CD8 T cells and Treg cells in peripheral blood were measured. It was observed that the levels of CD8 T cells in the IFN-γ-BMSC, sPD-1-BMSC, and IFN-γ + sPD-1-BMSC intervention groups were significantly higher than those in the model group (*P* < 0.01, *P* < 0.001) (**Figure 6B**). In contrast, the levels of Treg cells in the IFN-γ-BMSC, sPD-1-BMSC, and IFN-γ + sPD-1-BMSC intervention groups were significantly lower than those in the model group (*P* < 0.05, *P* < 0.001) (**Figure 6C**).

### 3.7 IFN-γ- and sPD-1-overexpressing BMSCs downregulates PI3K/AKT expression

**Figure 7:**
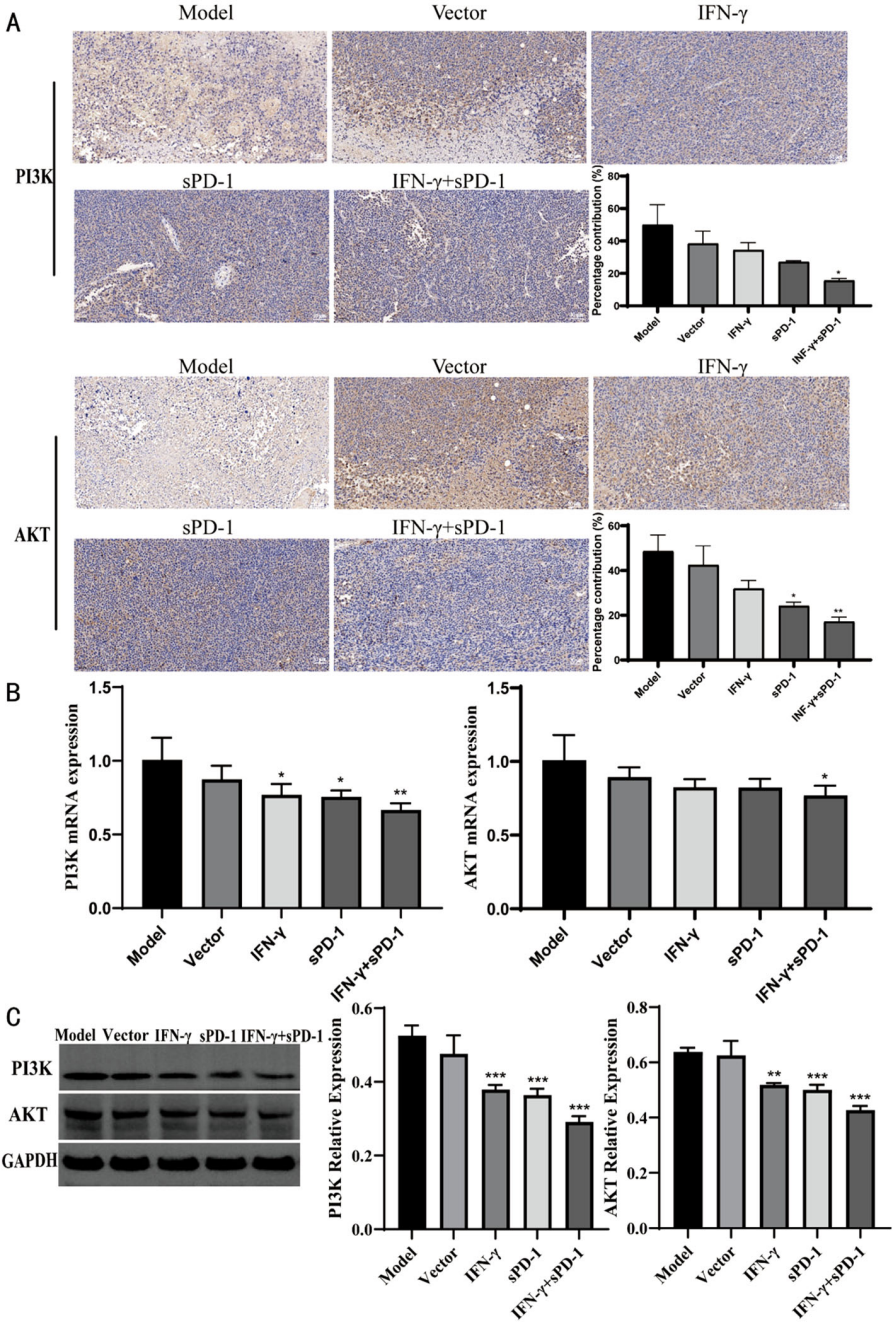
Expression of PI3K/AKT in tumor tissue of each group. **(A)** Immunohistochemical results of PI3K/AKT in each group; **(B)** qPCR results of PI3K/AKT in each group; **(C)** WB results of PI3K/AKT in each group. * *P*<0.05, **p<0.01and ***p<0.001 vs Model group.

Immunohistochemical analysis revealed that relative positive expression of PI3K and AKT protein expression was significantly reduced in the IFN-γ+sPD-1 group compared to the model group (*P* < 0.05, *P* < 0.01) (**Figure 7A**). RT-qPCR and WB analysis confirmed that PI3K and AKT mRNA and protein levels were markedly downregulated in the IFN-γ+sPD-1 group (*P* < 0.05, *P* < 0.01 and *P* < 0.001) (**Figures 7B, C**).

### 3.8 IFN-γ- and sPD-1-overexpressing BMSCs reduces PD-L1 expression in tumor tissue

**Figure 8:**
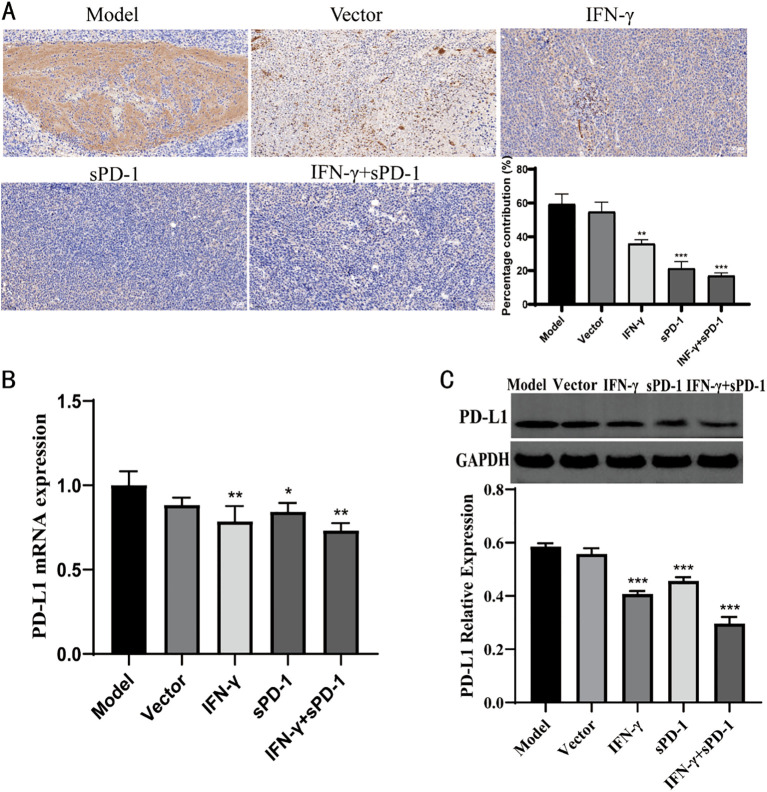
Expression of PD-L1 in tumor tissue of each group. **(A)** Immunohistochemical results of PD-L1 in each group; **(B)** qPCR results of PD-L1 in each group; **(C)** WB results of PD-L1 in each group.* *P*<0.05, **p<0.01 and ***p<0.001 vs Model group.

Immunohistochemical analysis showed that, compared to the model group, the relative positive expression of PD-L1 protein in the IFN-γ intervention group, sPD-1 intervention group, and sPD-1 + IFN-γ intervention group was significantly reduced (P < 0.01, P < 0.001) (**Figure 8A**). RT-qPCR and WB was used to measure the mRNA and protein expression levels of PD-L1 in the tumor tissues of mice from each group. The results showed that, except for the sPD-1 intervention groups, the mRNA and protein expression levels of PD-L1 in the other intervention groups were significantly lower than those in the model group (*P* < 0.05, *P* < 0.01) (**Figures 8B, C**).

## 4 Discussion

Immune checkpoint inhibitors exert their therapeutic effects by interrupting the PD-1/PD-L1 interaction between T lymphocytes and neoplastic cells, thereby reactivating cytotoxic T cells and restoring their antitumor capabilities (14). This immunotherapeutic strategy provides broad clinical applicability and durable survival benefits, establishing novel treatment paradigms for lung adenocarcinoma patients (15, 16). In this study, sPD-1, as an immune checkpoint inhibitor, demonstrated significant effects on immune therapy for lung adenocarcinoma, leveraging the homing capability of BMSCs to target tumor tissues and the immune-regulatory role of IFN-γ (17, 18). Notably, sPD-1 is a monomeric protein, and studies suggest that it has a dual role: besides blocking the PD-L1 pathway on tumor cells, it can also enhance immune responses by interacting with immune cells (19, 20). Thus, research has combined sPD-1 with other immunotherapy targets to improve therapeutic efficacy.

BMSCs exhibit dual regulatory roles in lung adenocarcinoma (21), dynamically influencing tumor progression through CXCR4-mediated homing to the microenvironment where they secrete pro-angiogenic (VEGF), pro-metastatic (IL-6, TGF-β), and immunosuppressive factors, while paradoxically suppressing metastasis via TRAIL-induced apoptosis or differentiation into cancer-associated fibroblasts under specific conditions, with these opposing effects governed by BMSC heterogeneity and inflammatory signaling dynamics within the tumor niche (22).

Concurrently, IFN-γ secreted by Th1-polarized CD4+ T lymphocytes orchestrates macrophage activation, NK cell cytotoxicity, and T cell effector functions, conferring comprehensive antiviral and antitumor responses (23, 24). Of particular relevance, IFN-γ has been shown to promote s proliferation (25) and enhance their immune-modulatory capacity *in vitro (* 26), which is important for improving cell therapy efficacy, reducing adverse reactions, and lowering treatment costs (27). And IFN-γ enhances the immunomodulatory capacity of BMSCs through activation of the JAK-STAT1 signaling pathway, which upregulates key mediators such as indoleamine 2,3-dioxygenase (28).

Our experimental data revealed that the BMSCs co-expressing IFN-γ and sPD-1 maintained robust biological activity and genetic stability, providing a theoretical feasibility for immune therapy in lung adenocarcinoma.

Therapeutic suppression of tumor proliferation and metastatic potential represents a cornerstone of cancer management (29). Our findings demonstrate that IFN-γ- and sPD-1-overexpressing BMSCs significantly suppressed the growth of Lewis and A549 cells. Compared to single-gene transfection, IFN-γ- and sPD-1-overexpressing BMSCs had a more pronounced inhibitory effect on cell proliferation. Furthermore, co-culture experiments revealed a reduction in migration and invasion abilities of lung adenocarcinoma cells after BMSC intervention. Additionally, IFN-γ- and sPD-1-overexpressing BMSCs significantly promoted senescence in lung adenocarcinoma cells.

In animal models, IFN-γ- and sPD-1-overexpressing BMSCs significantly suppressed tumor growth in mice. Compared to single-gene overexpression, this combination showed superior tumor-suppressive effects. Bcl-2 and Bax play crucial roles in regulating apoptosis in tumor cells. When Bcl-2 is overexpressed, it forms heterodimers with Bax, reducing mitochondrial membrane permeability and inhibiting apoptosis (30). Numerous studies have suggested that Bcl-2 is a favorable prognostic marker, with its expression generally associated with improved cancer patient outcomes (31). Our study confirmed that IFN-γ- and sPD-1-overexpressing BMSCs significantly increased the relative positive expression of Bax protein, decreased the relative positive expression of Bcl-2, and increased the area of apoptosis-positive regions and the number of senescent tumor cells in mouse tumors, further confirming the effectiveness of their antitumor actions.

Histopathological analysis using HE staining revealed that, compared to the model group, the lung tissue in the IFN-γ and sPD-1 overexpression BMSC intervention group showed clearer structure, with no significant widening of alveolar septa or inflammatory cell infiltration in the lung interstitium. The cell nuclei showed lighter atypical staining, suggesting that this intervention significantly reduced lung tissue damage in mice, improved pathological morphology of lung tissue in lung adenocarcinoma mice, and reduced the occurrence of metastasis. This may be related to the intervention improving the immune status of the mice, enhancing their ability to surveil and eliminate tumors. Treg cells play a unique immunoregulatory role (32), maintaining immune system homeostasis, inhibiting the activation of other immune cells, and controlling excessive responses to foreign antigens[ (33, 34). In this study, the Treg levels in the intervention groups were lower than in the model group, with the most significant effect observed in the IFN-γ and sPD-1 overexpressing BMSC group. This suggests that the overexpression of IFN-γ and sPD-1 in BMSCs significantly reduced Treg levels in lung adenocarcinoma mice, inhibited tumor cell immune evasion, enhanced the immune function of tumor-bearing mice, and thereby strengthened the antitumor immune response.

Furthermore, this study explored the mechanism behind the inhibition of lung adenocarcinoma. After the intervention with IFN-γ- and sPD-1-overexpressing BMSCs, the expression of PD-L1 protein in tumor cells significantly decreased. This combination treatment may enhance the efficacy of immune therapy by downregulating PD-L1 expression on tumor cells. The results confirm that IFN-γ- and sPD-1-overexpressing BMSCs produce a synergistic effect by lowering PD-L1 expression, thus enhancing the activity of immune effector cells and exerting antitumor effects. It may also induce apoptosis in lung adenocarcinoma cells, further inhibiting their proliferation and invasion. Possible mechanisms include activation of apoptotic pathways and upregulation of apoptotic genes, such as Bax and Bcl-2. Moreover, it may be related to the tumor-associated signaling pathway PI3K/AKT, which plays a key role in tumor progression by promoting cell proliferation, invasion, metastasis, and anti-apoptosis (35, 36). Our study found that IFN-γ- and sPD-1-overexpressing BMSCs significantly reduced the relative positive expression of PI3K and AKT in lung cancer tissues, blocking the signaling pathway and inhibiting tumor cell growth and metastasis. Although this study provides a preliminary exploration of the PI3K/AKT and PD-1/PD-L1 pathways, tumor development involves complex signaling networks, and further research is needed to fully reveal the mechanisms.

In conclusion, this study confirmed that IFN-γ- and sPD-1-overexpressing BMSCs exhibited significant effects in inhibiting the growth of lung adenocarcinoma cells and tumors in mice. These effects are mediated through various mechanisms, including inhibiting tumor cell growth, migration, and invasion, promoting apoptosis and senescence, modulating Treg cells, inhibiting the PI3K/AKT pathway, and reducing PD-L1 expression. This strategy significantly enhanced the immune function of lung adenocarcinoma mice, suppressed tumor growth and metastasis, and provides a new approach for immune therapy in lung adenocarcinoma, offering important theoretical and experimental support for future clinical research.

## Data Availability

The original contributions presented in the study are included in the article/**Supplementary Material**. Further inquiries can be directed to the corresponding authors.
