# Supplementary figures and images for: Study on the efficacy of IFN-γ- and sPD-1-overexpressing BMSCs in enhancing immune effects for the treatment of lung adenocarcinoma
Source: Front Immunol. 2025 Mar 13;16:1554467. doi: 10.3389/fimmu.2025.1554467 (PMC11965897; doi:10.3389/fimmu.2025.1554467)

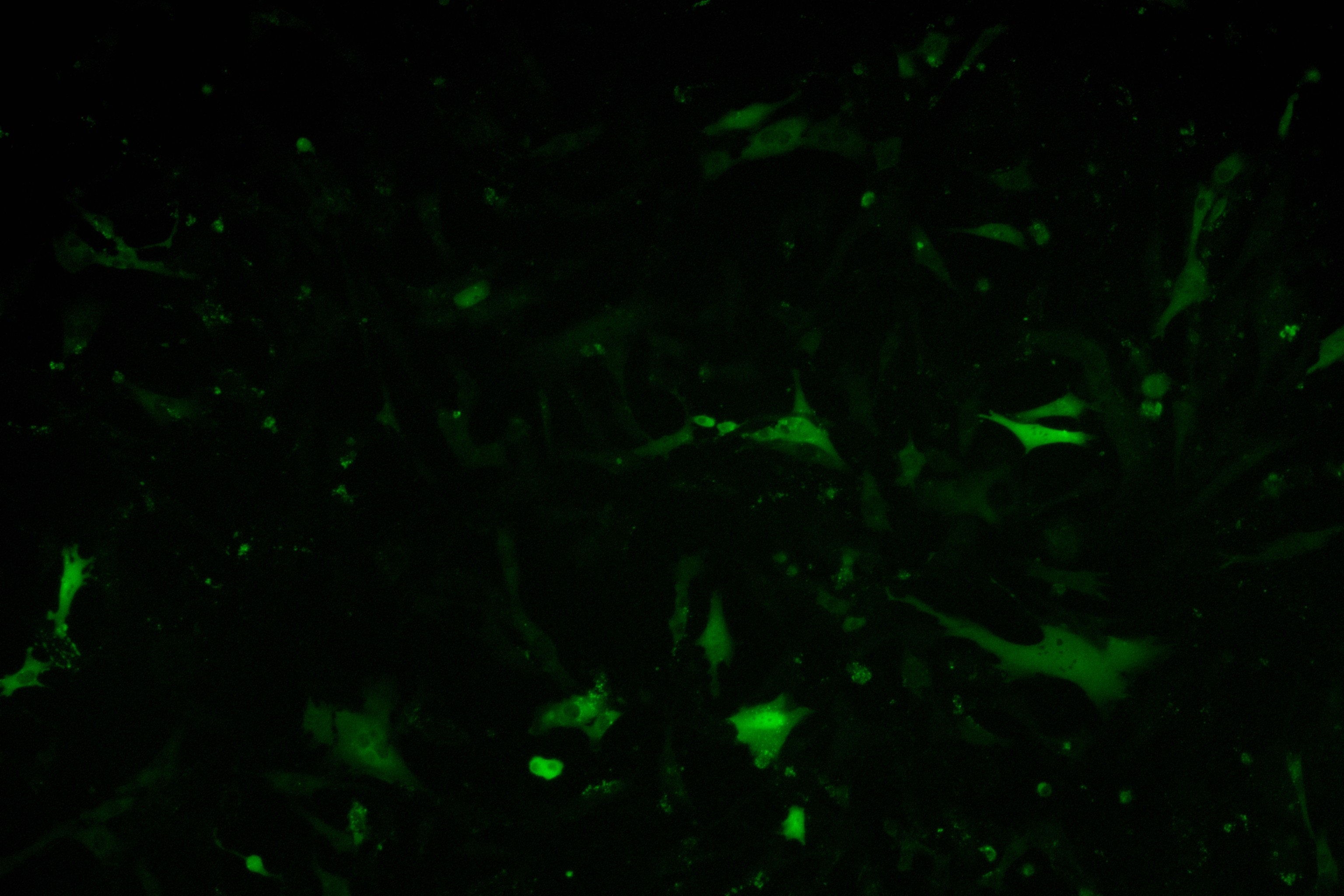

Supplement: Supplementary file 1 [file DataSheet1.zip › Immunofluorescence results of BMSCs overexpressing IFN-γ and sPD-1/IFN-γ 100x- (1).jpg]

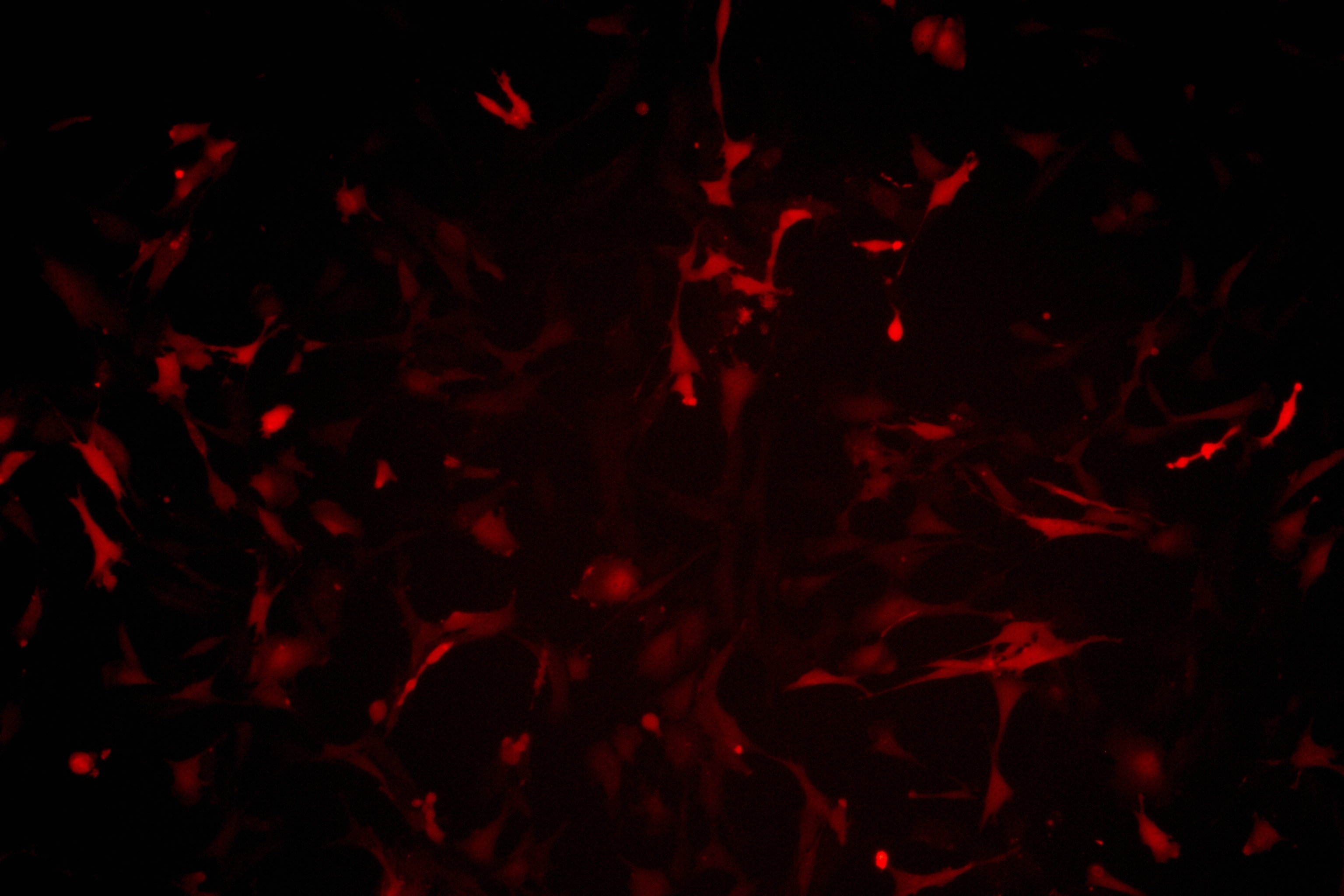

Supplement: Supplementary file 1 [file DataSheet1.zip › Immunofluorescence results of BMSCs overexpressing IFN-γ and sPD-1/sDP-1 100x.jpg]

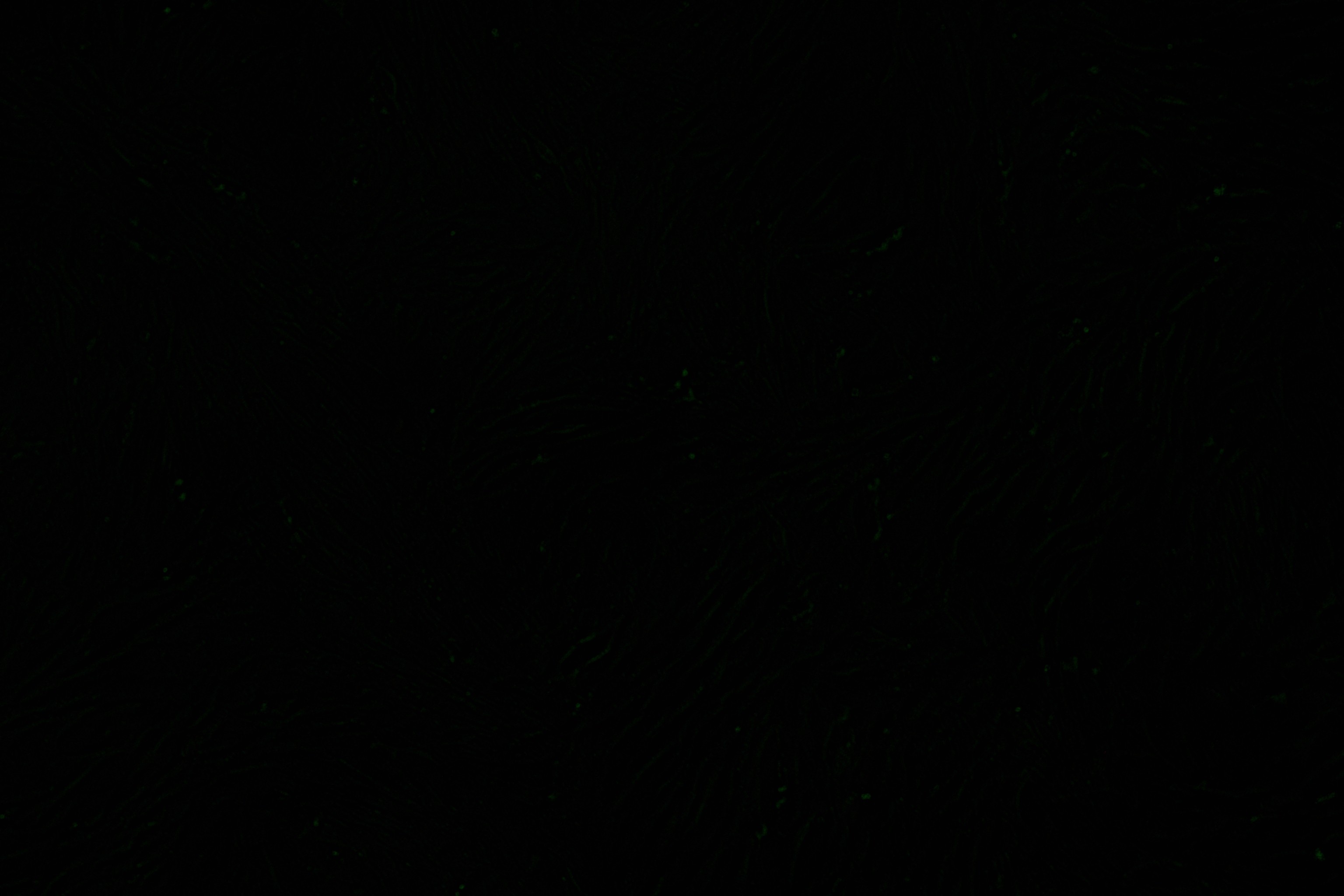

Supplement: Supplementary file 1 [file DataSheet1.zip › Immunofluorescence results of BMSCs overexpressing IFN-γ and sPD-1/vector 100X.jpg]

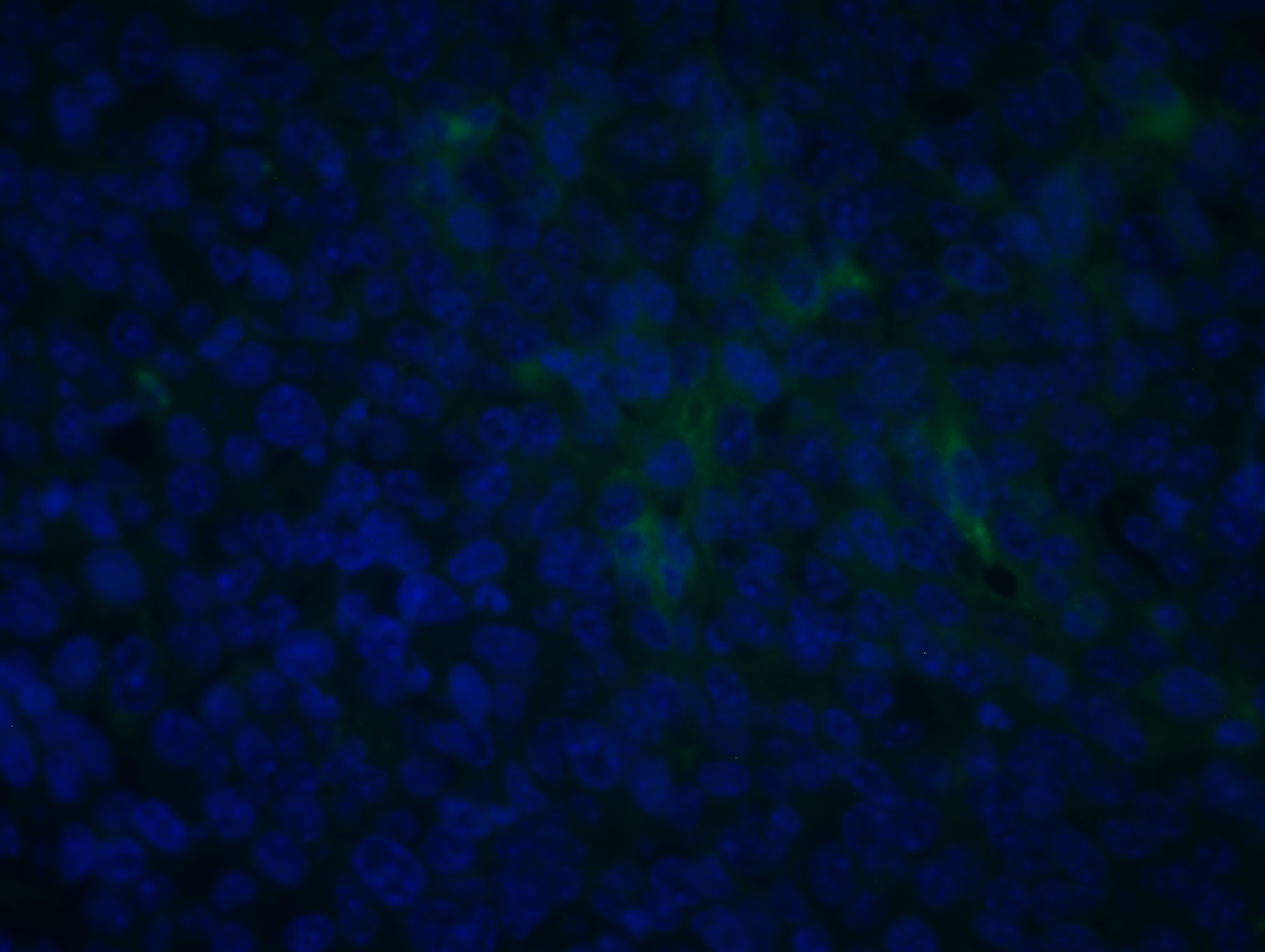

Supplement: Supplementary file 2 [file DataSheet2.zip › tunel/ifn-y+spd-1.png]

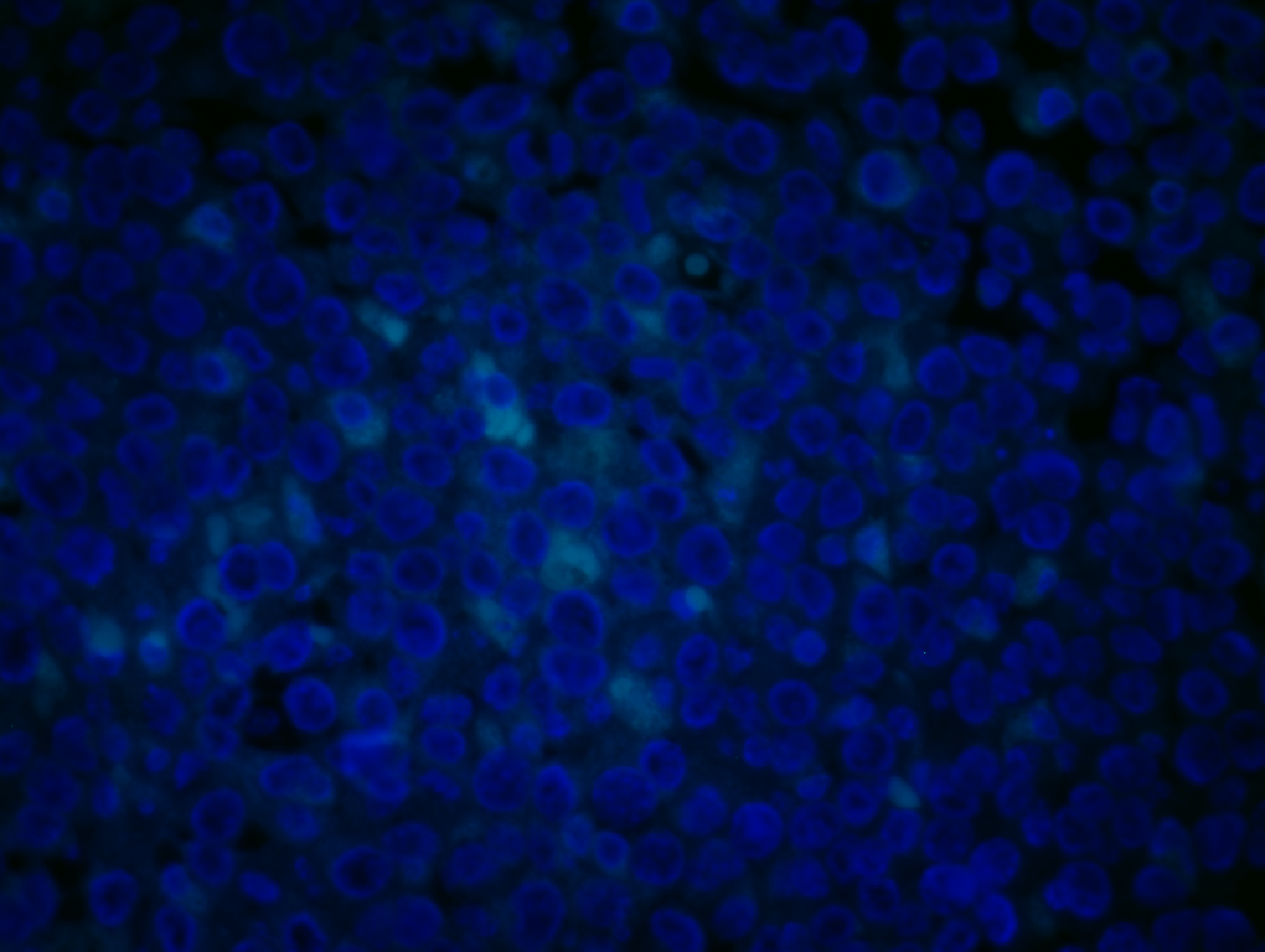

Supplement: Supplementary file 2 [file DataSheet2.zip › tunel/IFN-y.png]

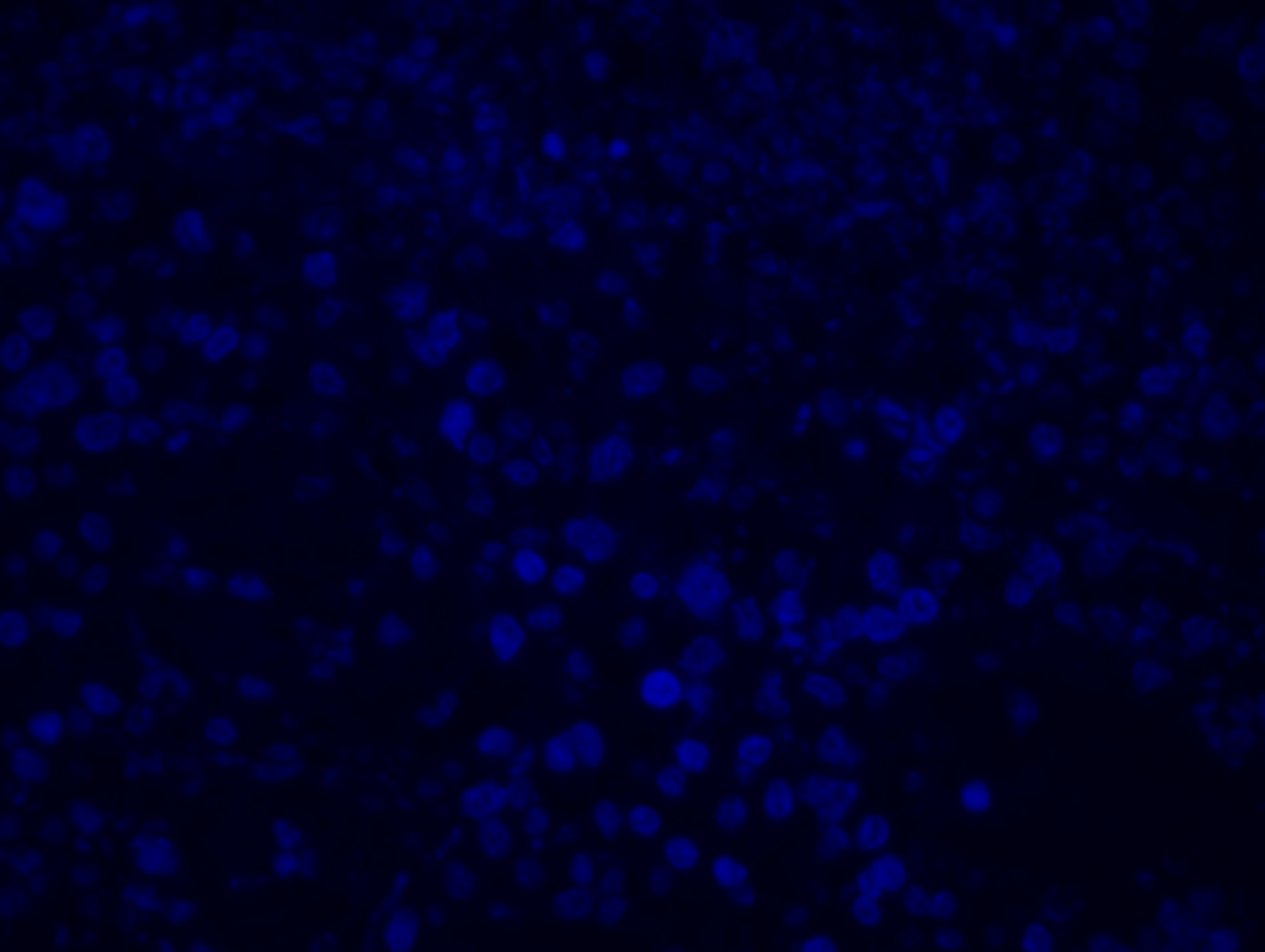

Supplement: Supplementary file 2 [file DataSheet2.zip › tunel/model.png]

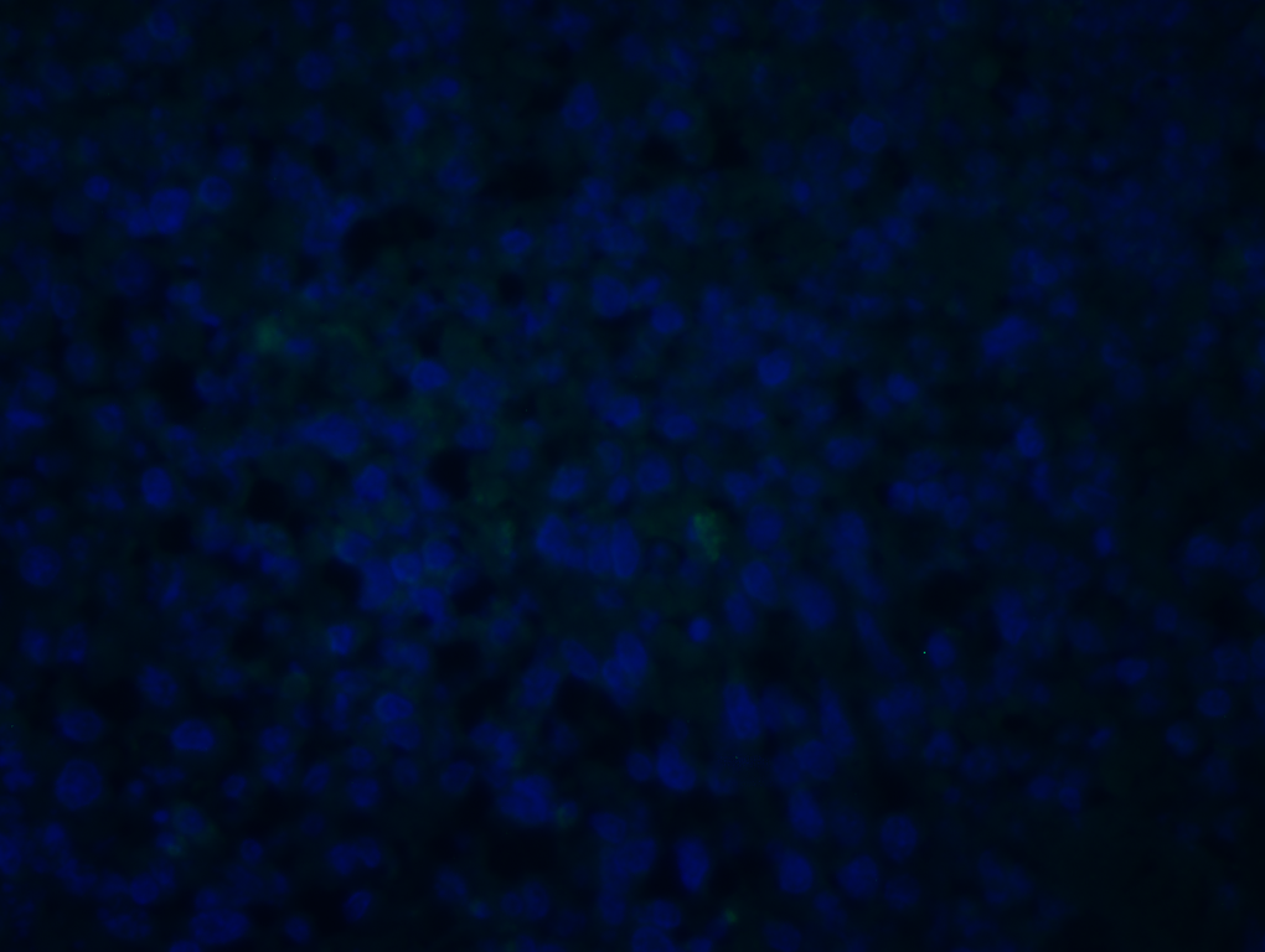

Supplement: Supplementary file 2 [file DataSheet2.zip › tunel/spd-1 .png]

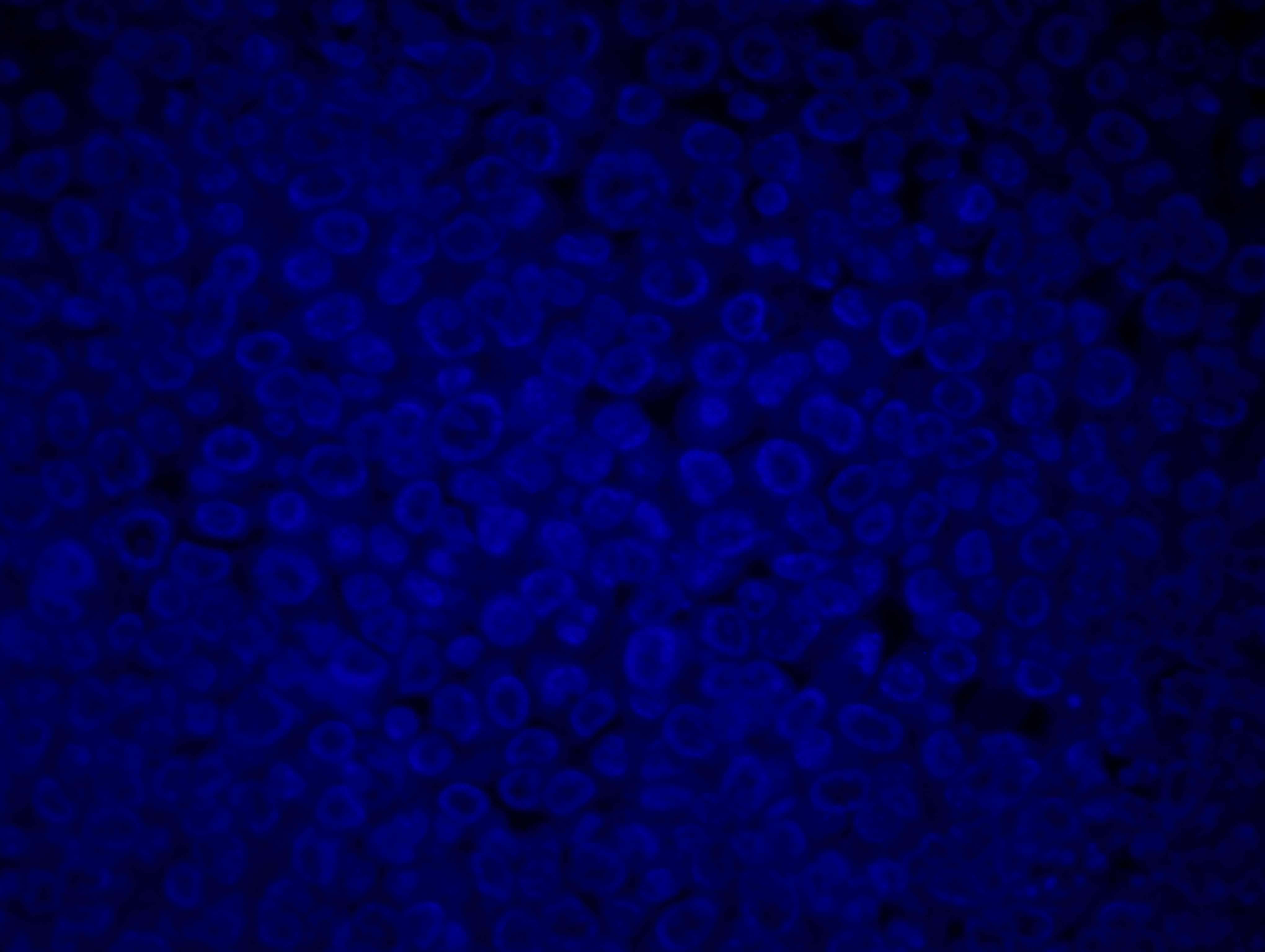

Supplement: Supplementary file 2 [file DataSheet2.zip › tunel/vector.png]

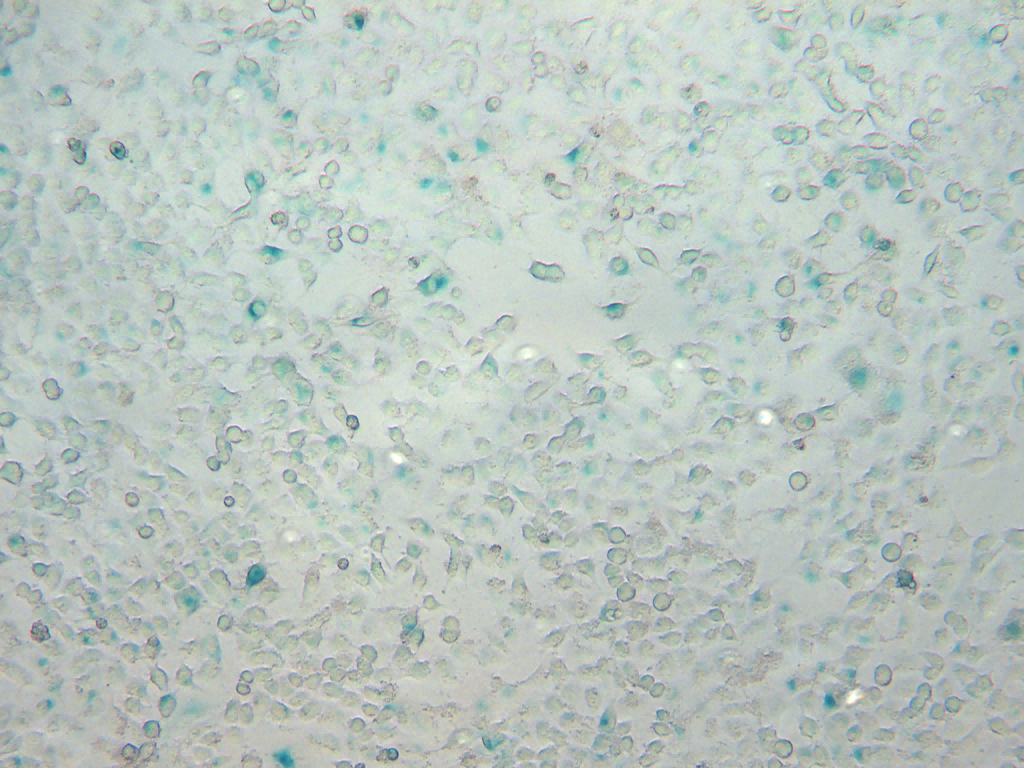

Supplement: Supplementary file 3 [file DataSheet3.zip › β-galactosidase assay showing the effect of BMSCs overexpressing IFN-γ and sPD-1/A549-Control (2).png]

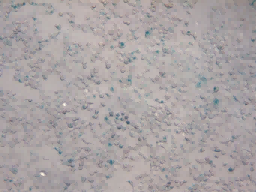

Supplement: Supplementary file 3 [file DataSheet3.zip › β-galactosidase assay showing the effect of BMSCs overexpressing IFN-γ and sPD-1/A549-IFN-y (2).png]

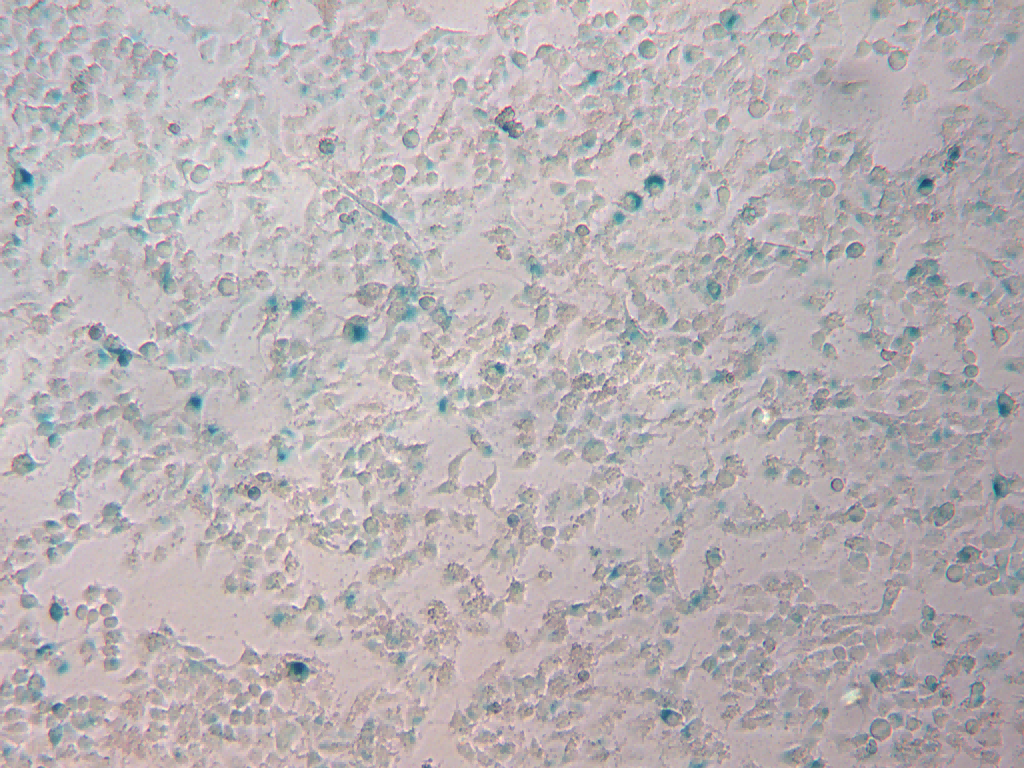

Supplement: Supplementary file 3 [file DataSheet3.zip › β-galactosidase assay showing the effect of BMSCs overexpressing IFN-γ and sPD-1/A549-IFN-γ+sPD-1 (2).png]

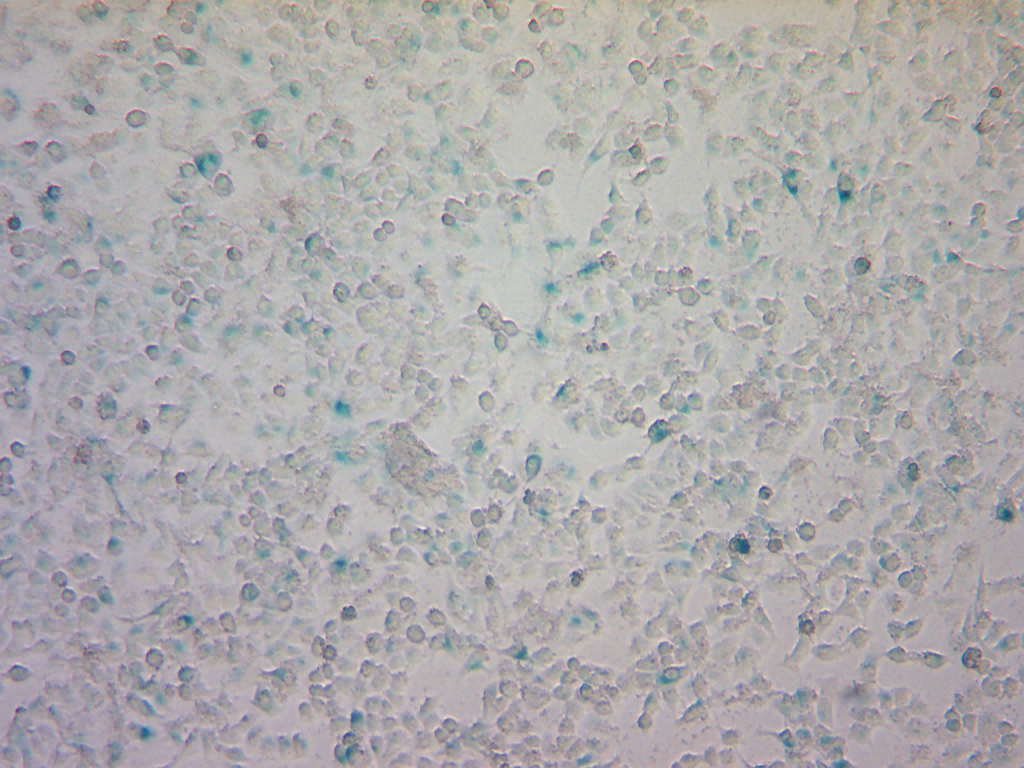

Supplement: Supplementary file 3 [file DataSheet3.zip › β-galactosidase assay showing the effect of BMSCs overexpressing IFN-γ and sPD-1/A549-sPD-1 (2).png]

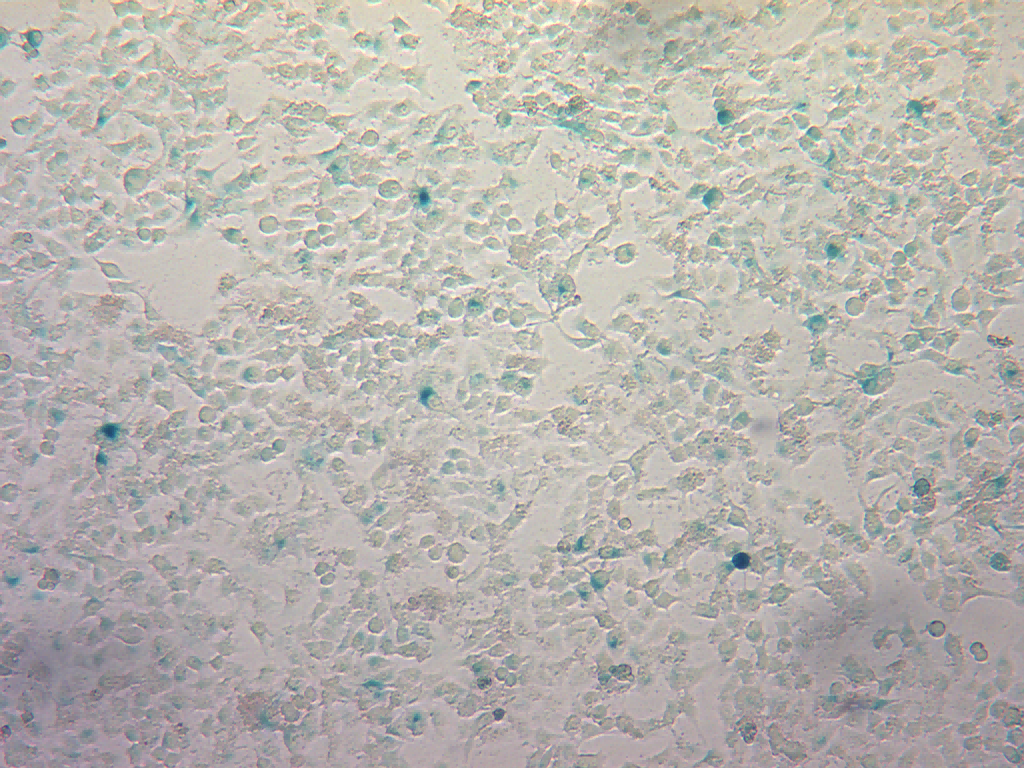

Supplement: Supplementary file 3 [file DataSheet3.zip › β-galactosidase assay showing the effect of BMSCs overexpressing IFN-γ and sPD-1/A549-Vector (2).png]

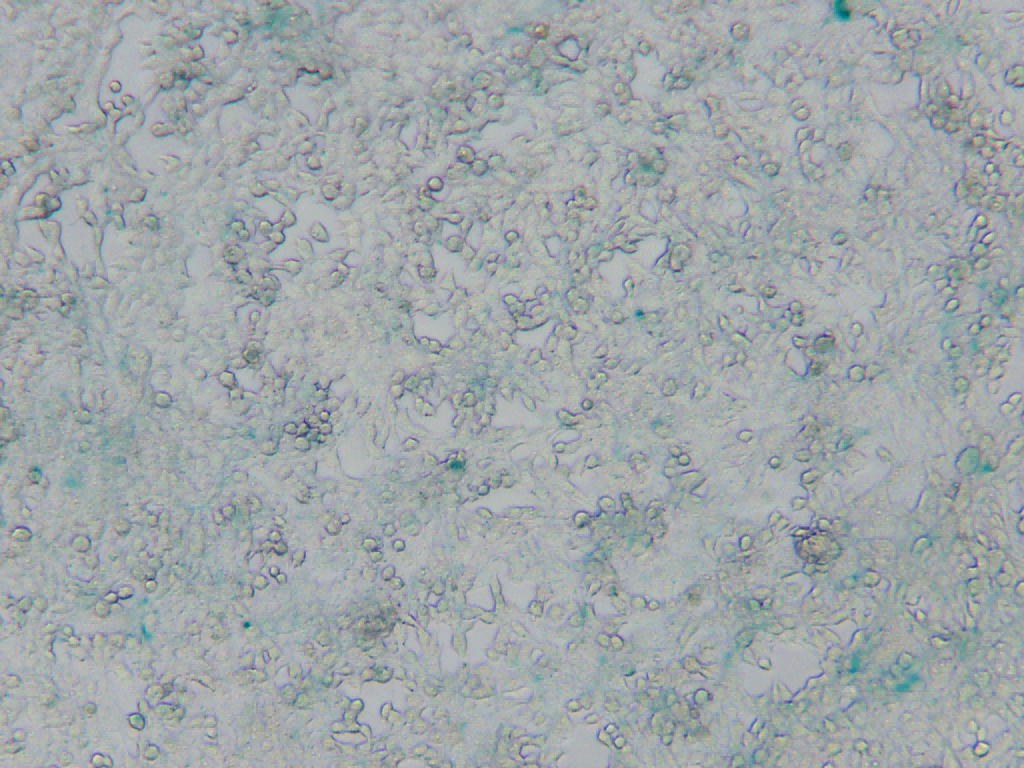

Supplement: Supplementary file 3 [file DataSheet3.zip › β-galactosidase assay showing the effect of BMSCs overexpressing IFN-γ and sPD-1/Lewis-Control (2).png]

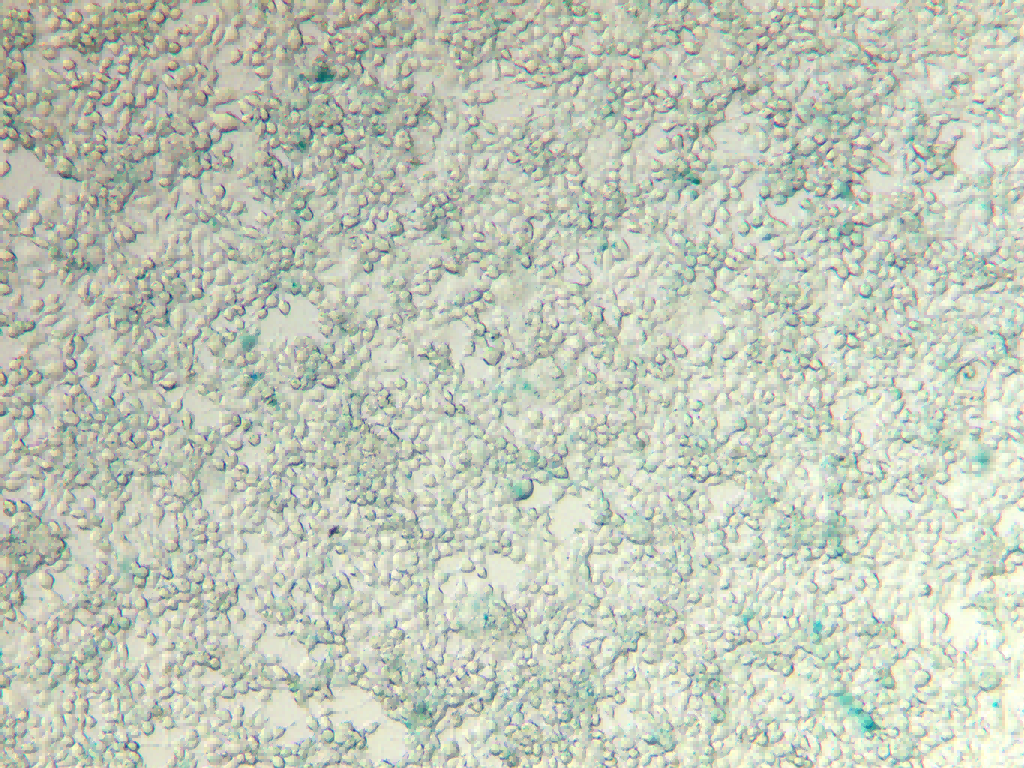

Supplement: Supplementary file 3 [file DataSheet3.zip › β-galactosidase assay showing the effect of BMSCs overexpressing IFN-γ and sPD-1/Lewis-IFN-y (2).png]

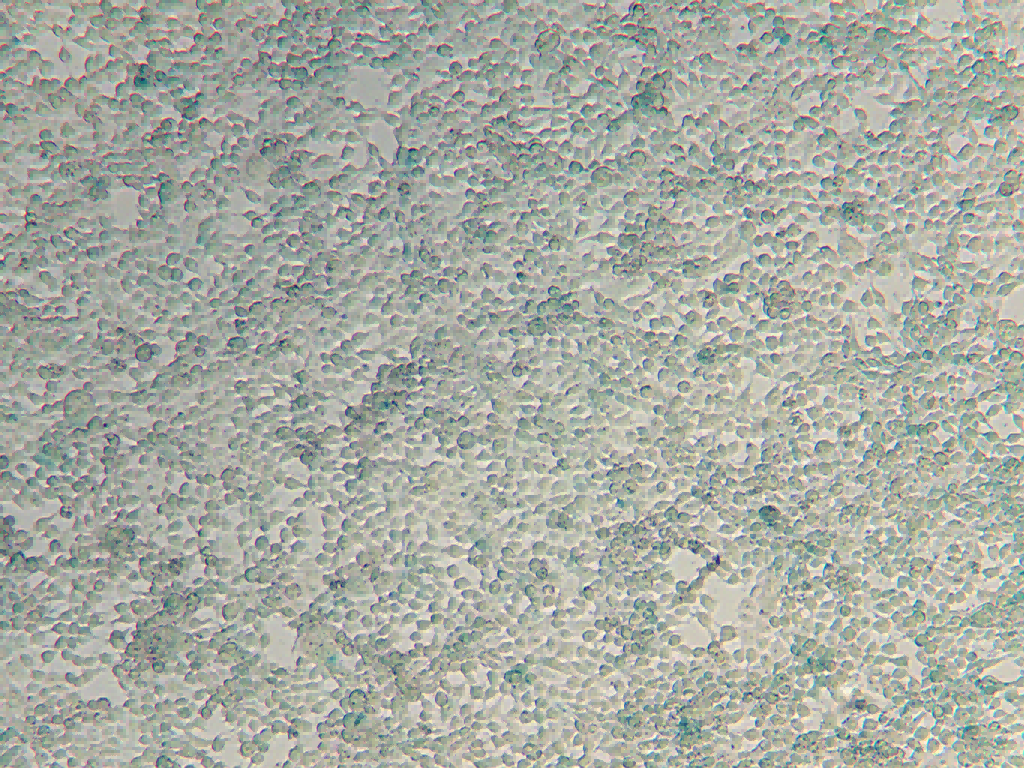

Supplement: Supplementary file 3 [file DataSheet3.zip › β-galactosidase assay showing the effect of BMSCs overexpressing IFN-γ and sPD-1/Lewis-IFN-y+sPD-1 (2).png]

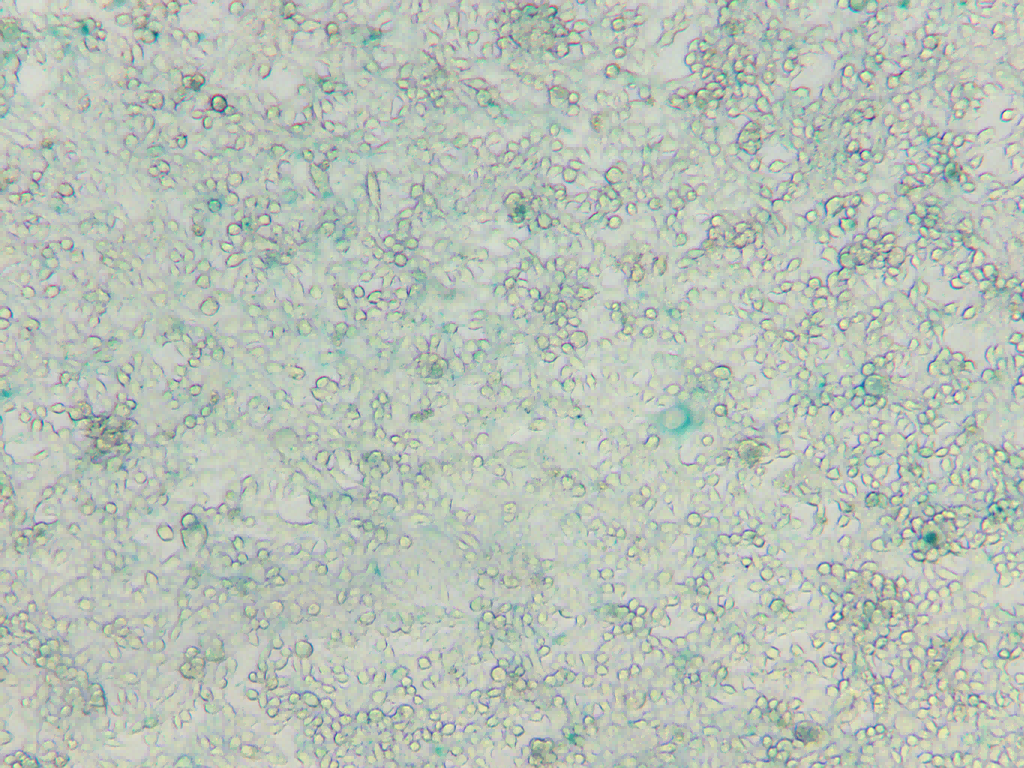

Supplement: Supplementary file 3 [file DataSheet3.zip › β-galactosidase assay showing the effect of BMSCs overexpressing IFN-γ and sPD-1/Lewis-sPD-1 (2).png]

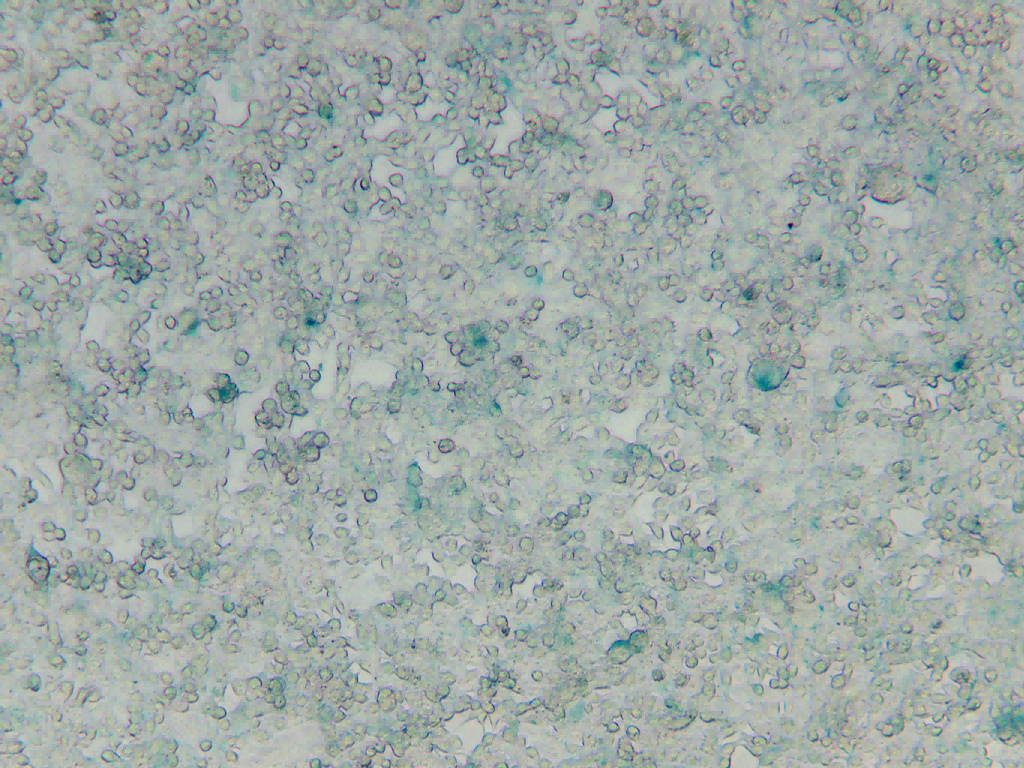

Supplement: Supplementary file 3 [file DataSheet3.zip › β-galactosidase assay showing the effect of BMSCs overexpressing IFN-γ and sPD-1/Lewis-Vector (2).png]

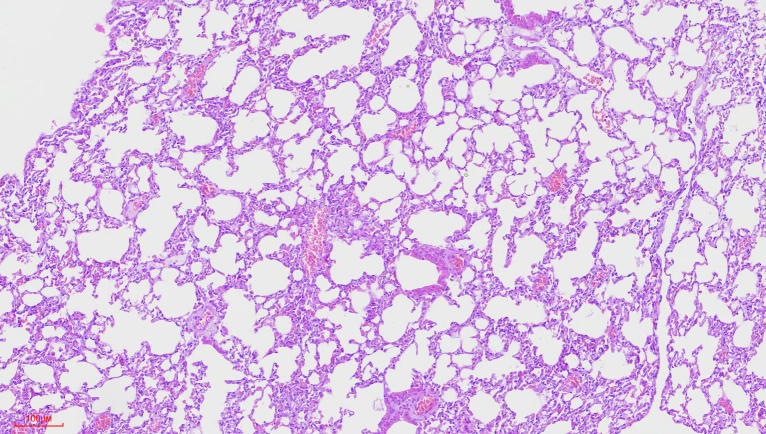

Supplement: Supplementary file 4 [file DataSheet4.zip › HE/lung/Control.png]

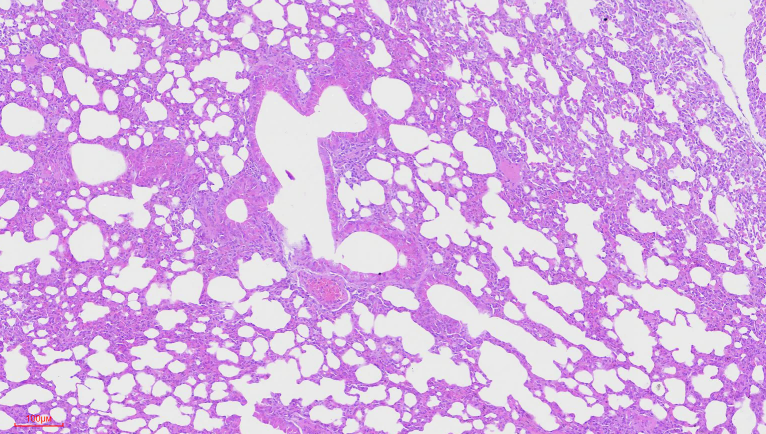

Supplement: Supplementary file 4 [file DataSheet4.zip › HE/lung/IFN-y+sPD-1.png]

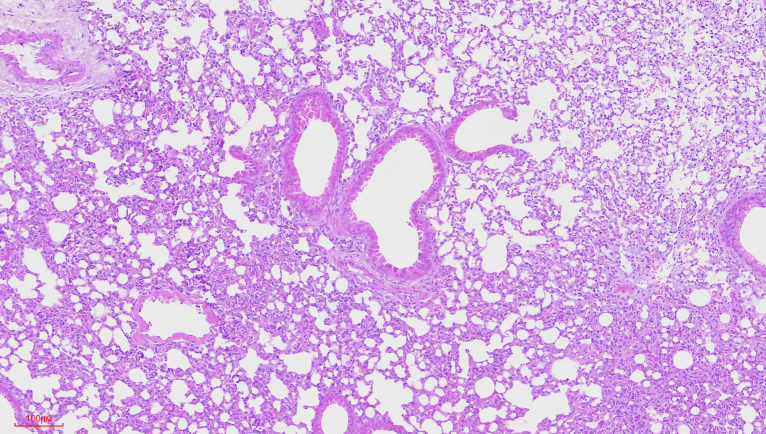

Supplement: Supplementary file 4 [file DataSheet4.zip › HE/lung/IFN-y.png]

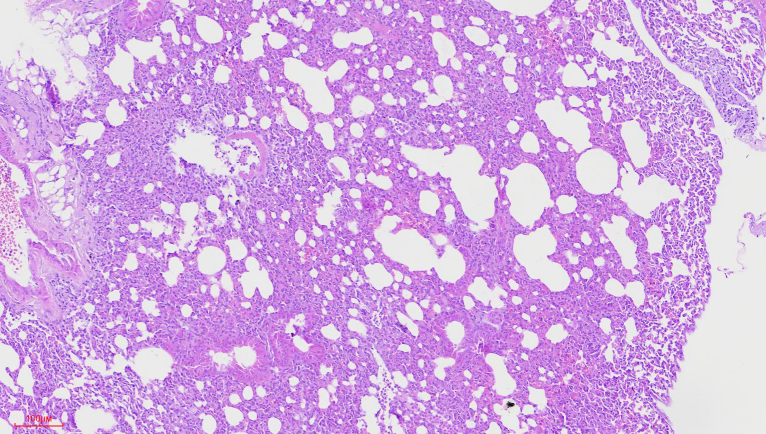

Supplement: Supplementary file 4 [file DataSheet4.zip › HE/lung/Model.png]

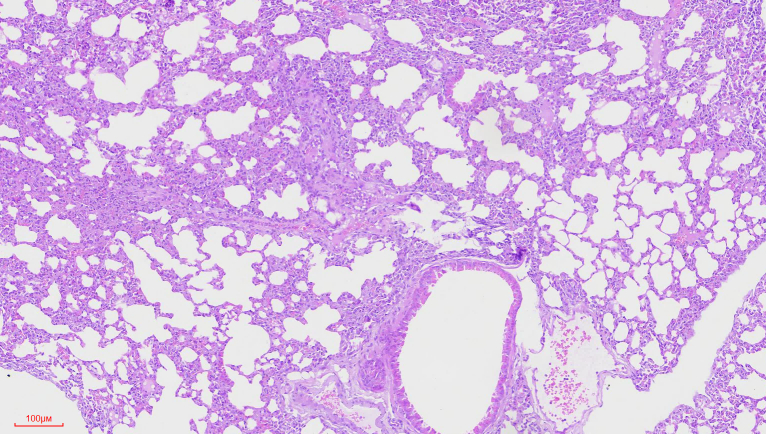

Supplement: Supplementary file 4 [file DataSheet4.zip › HE/lung/sPD-1.png]

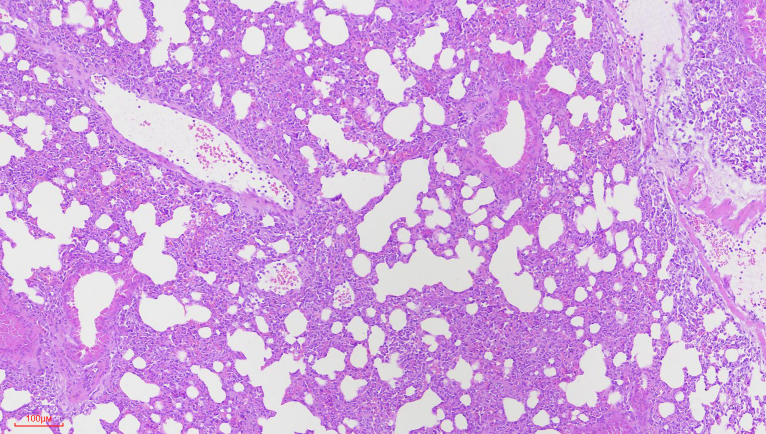

Supplement: Supplementary file 4 [file DataSheet4.zip › HE/lung/vector.png]

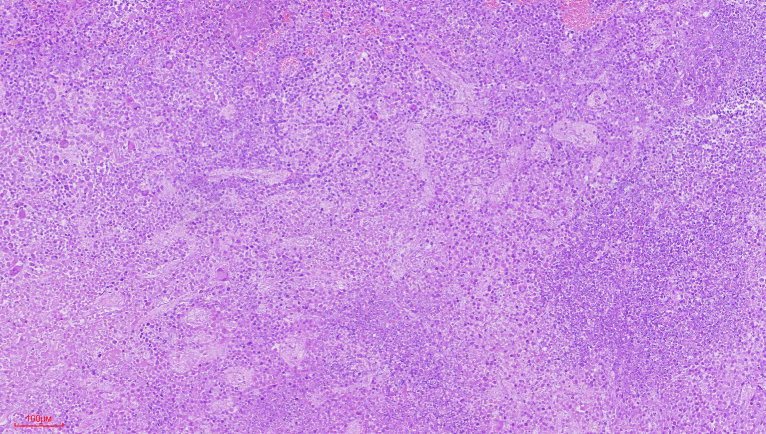

Supplement: Supplementary file 4 [file DataSheet4.zip › HE/Tumor/ifn-y+spd-.png]

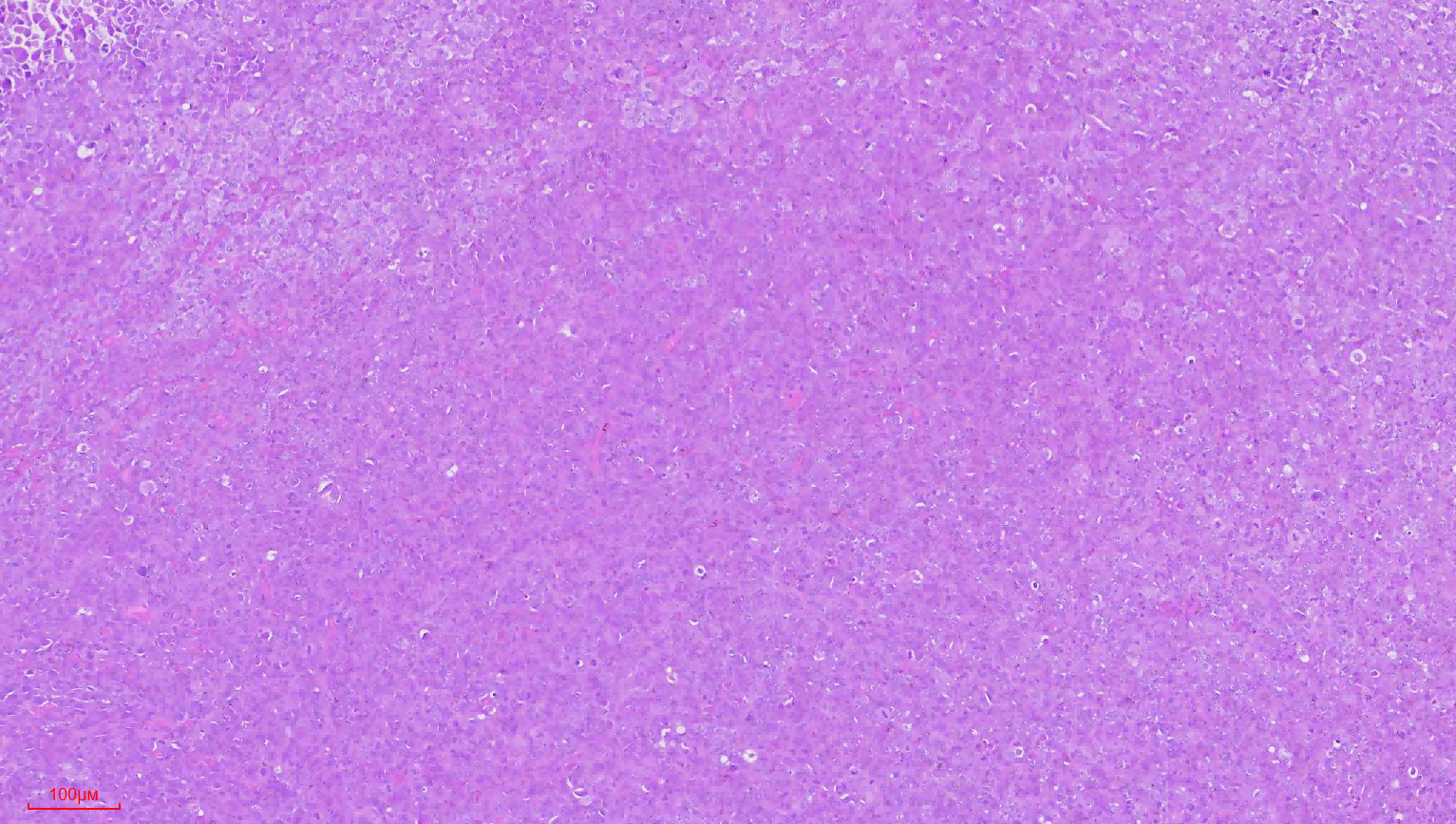

Supplement: Supplementary file 4 [file DataSheet4.zip › HE/Tumor/ifn-y.png]

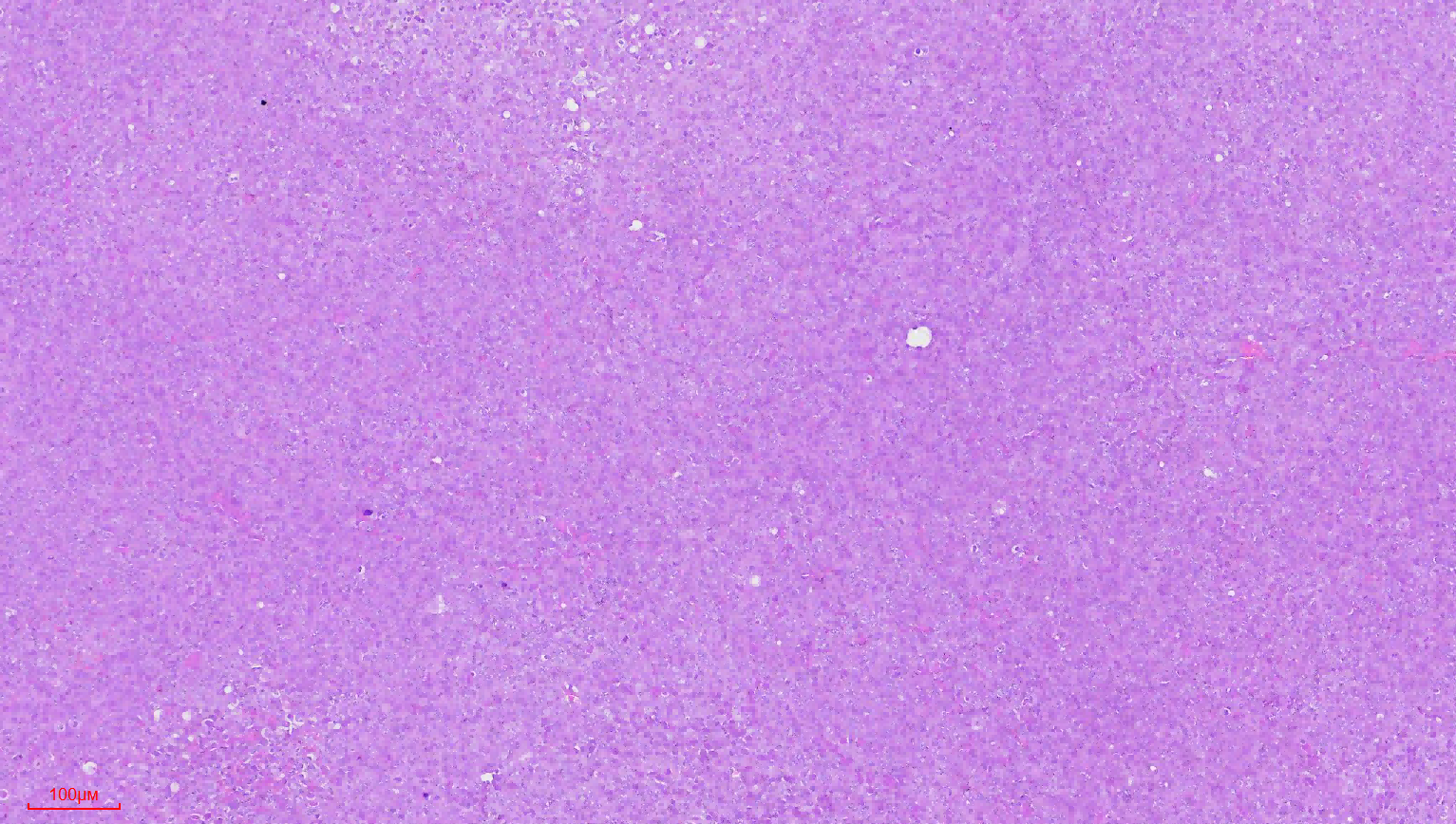

Supplement: Supplementary file 4 [file DataSheet4.zip › HE/Tumor/model.png]

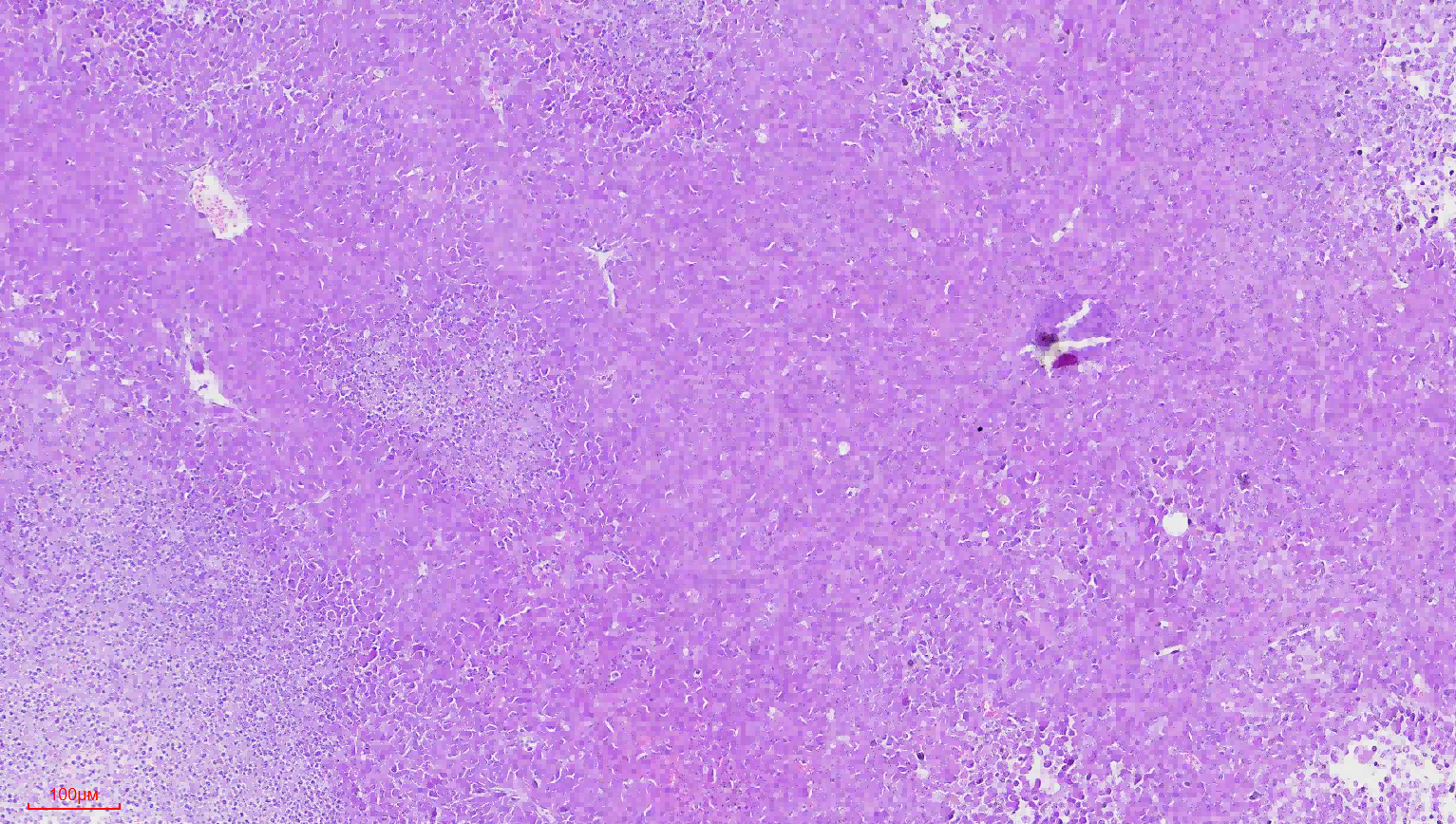

Supplement: Supplementary file 4 [file DataSheet4.zip › HE/Tumor/spd-1.png]

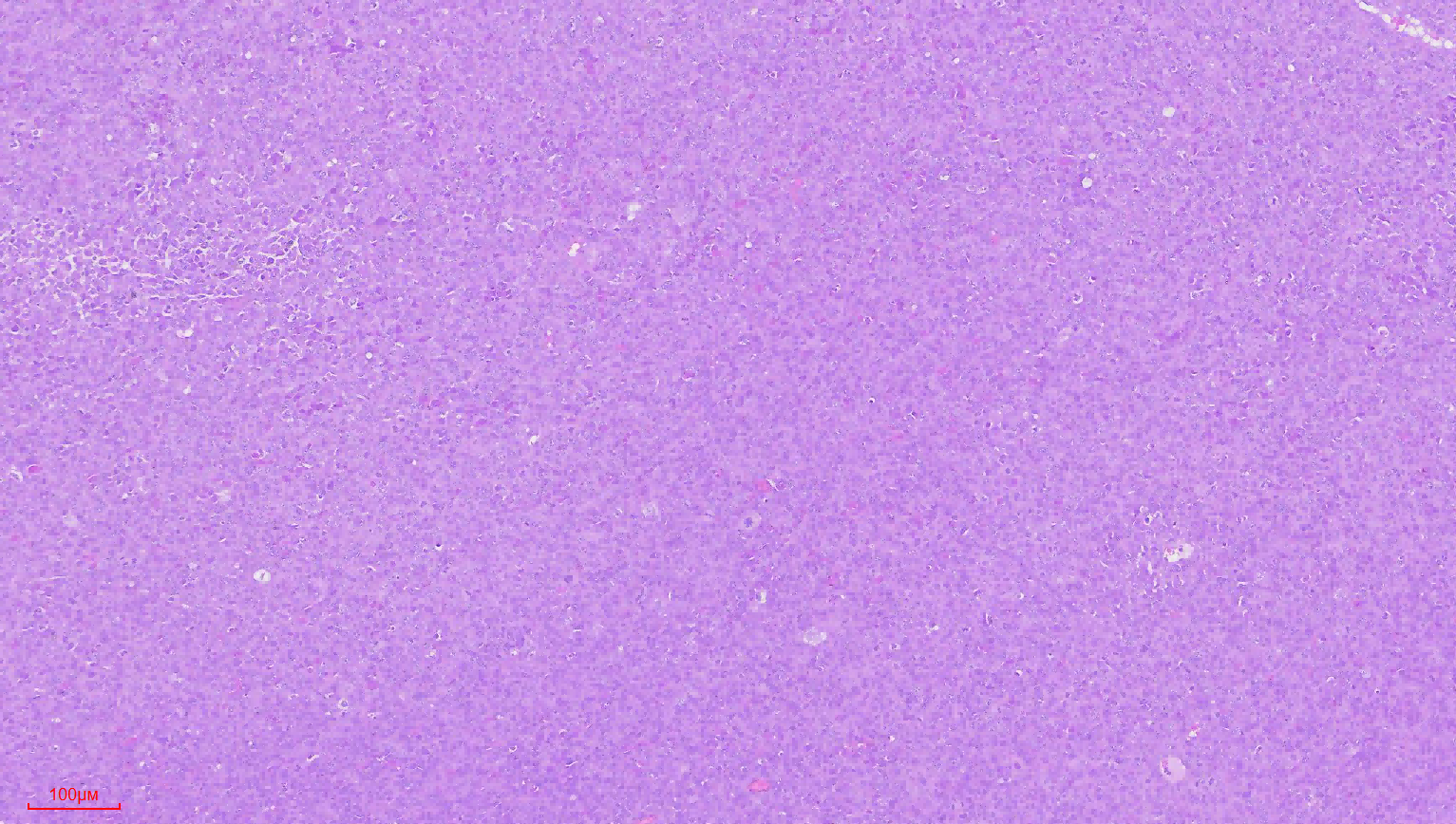

Supplement: Supplementary file 4 [file DataSheet4.zip › HE/Tumor/vector.png]

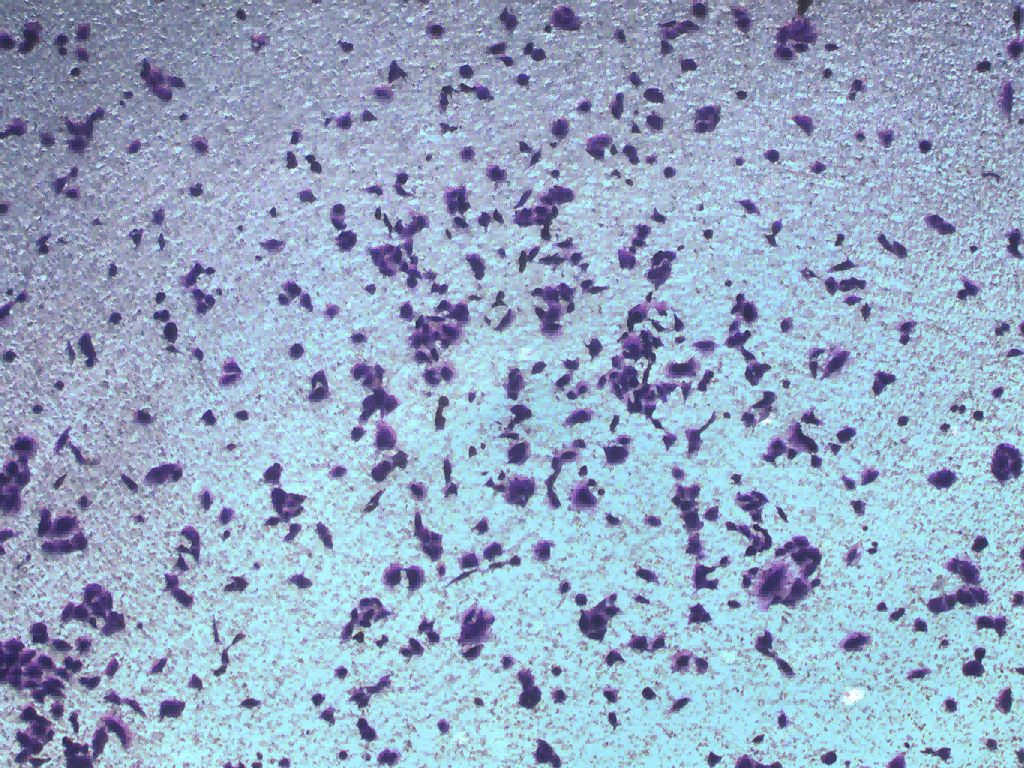

Supplement: Supplementary file 5 [file DataSheet5.zip › Transwell cell invasion assay detecting the invasion ability of lung adenocarcinoma cells/A549-control.png]

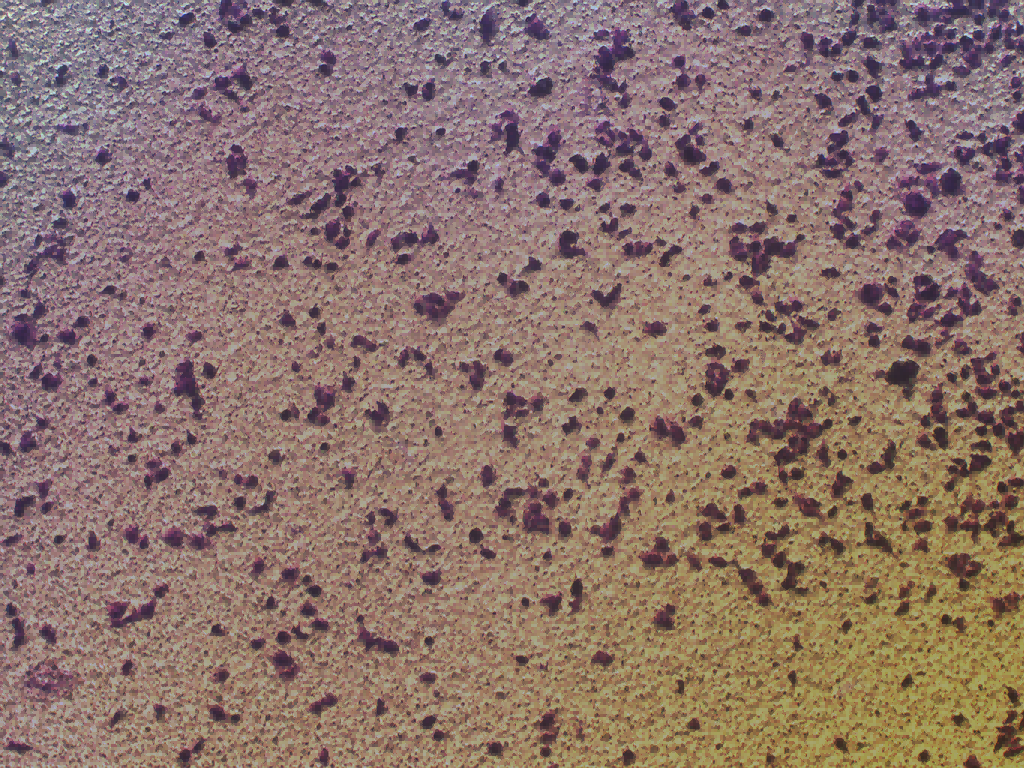

Supplement: Supplementary file 5 [file DataSheet5.zip › Transwell cell invasion assay detecting the invasion ability of lung adenocarcinoma cells/A549-IFN-y 2.png]

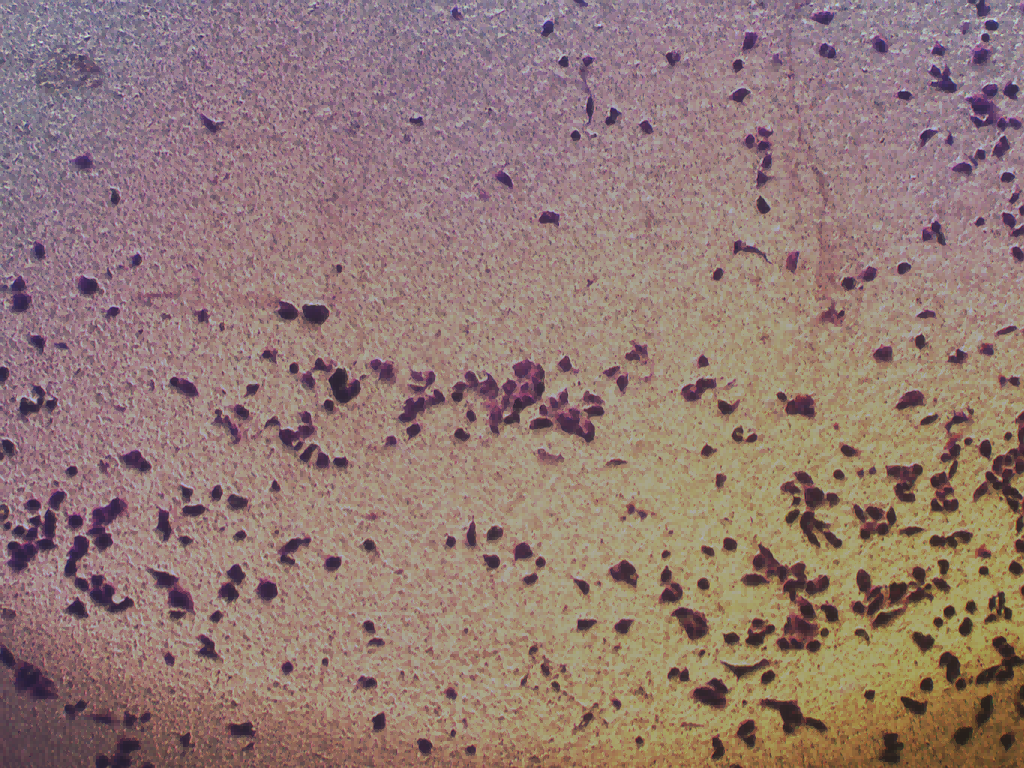

Supplement: Supplementary file 5 [file DataSheet5.zip › Transwell cell invasion assay detecting the invasion ability of lung adenocarcinoma cells/A549-ifn-y+spd-1.png]

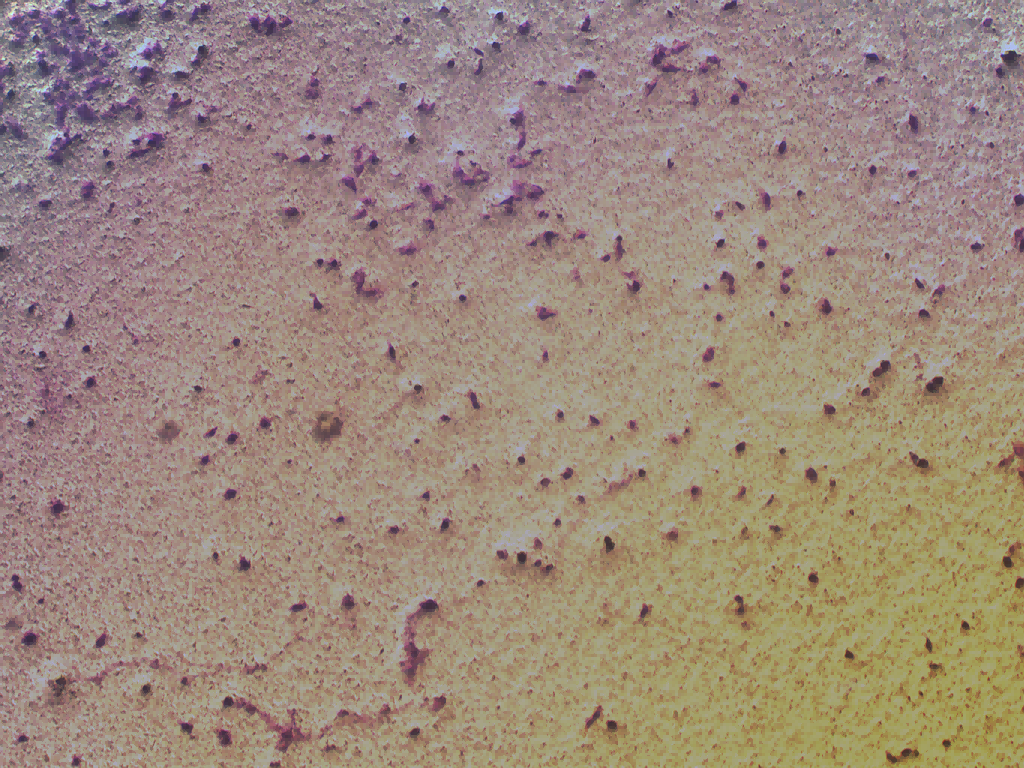

Supplement: Supplementary file 5 [file DataSheet5.zip › Transwell cell invasion assay detecting the invasion ability of lung adenocarcinoma cells/A549-SPD-1 2.png]

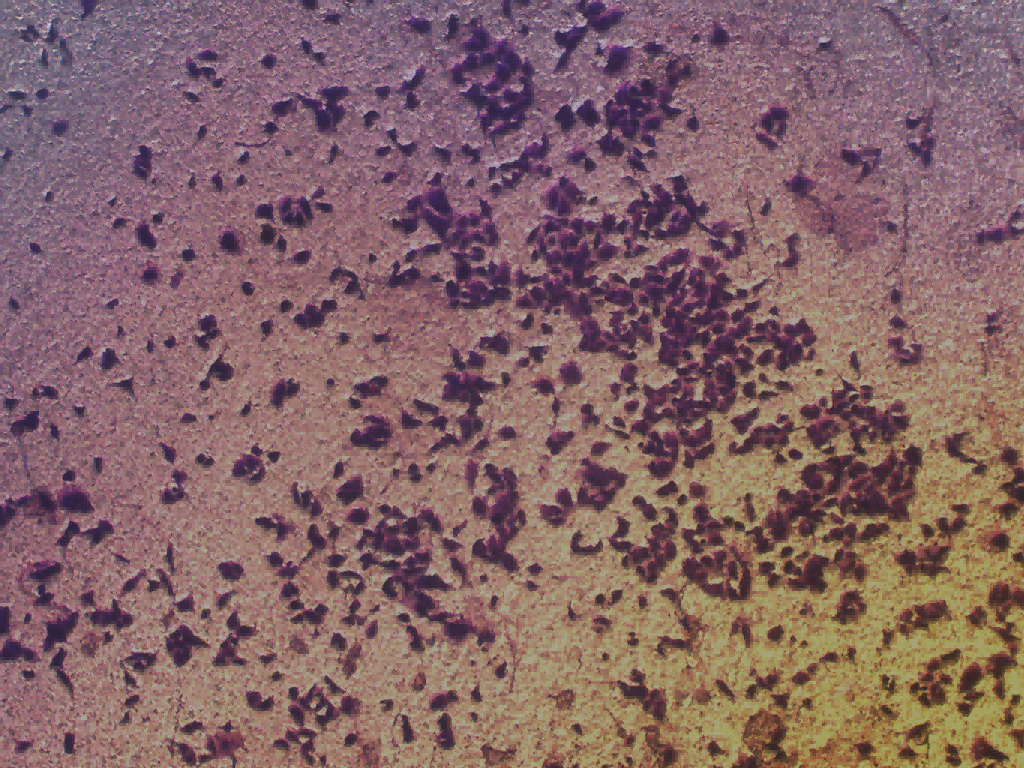

Supplement: Supplementary file 5 [file DataSheet5.zip › Transwell cell invasion assay detecting the invasion ability of lung adenocarcinoma cells/A549-vector.png]

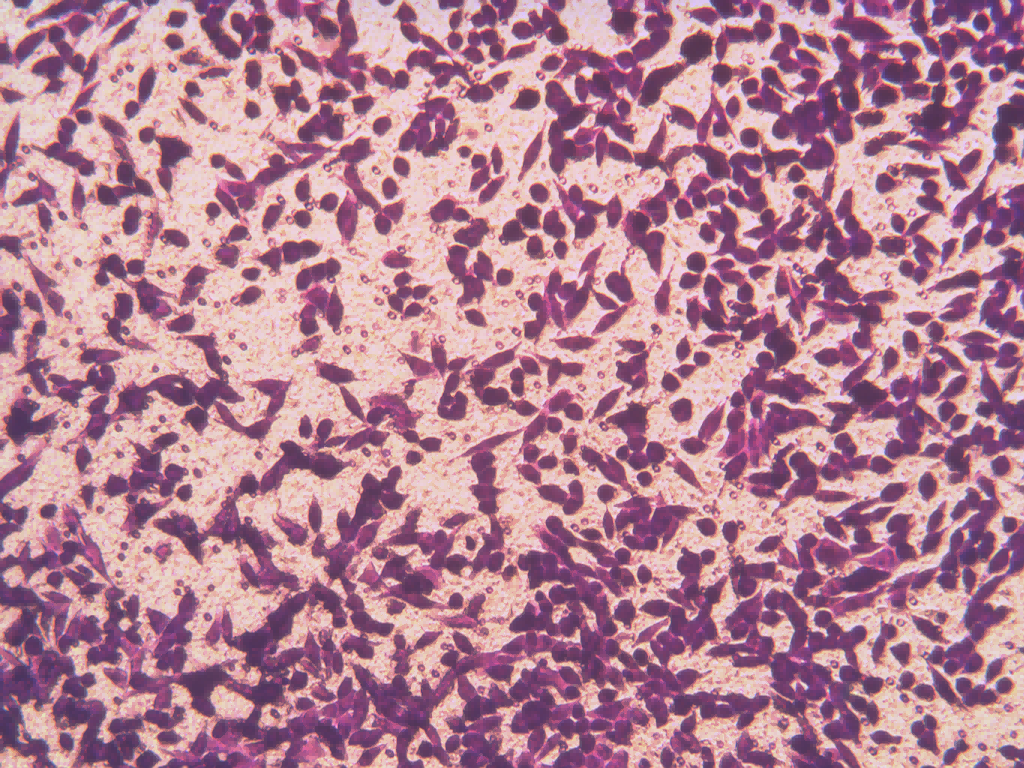

Supplement: Supplementary file 5 [file DataSheet5.zip › Transwell cell invasion assay detecting the invasion ability of lung adenocarcinoma cells/lewis-control.png]

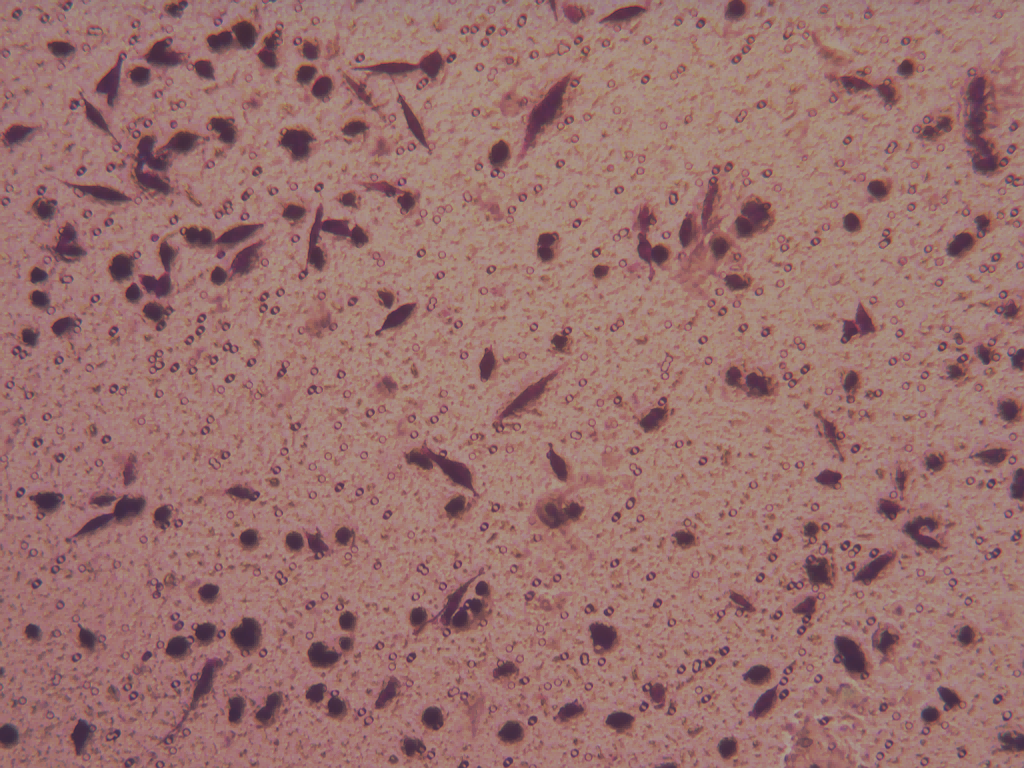

Supplement: Supplementary file 5 [file DataSheet5.zip › Transwell cell invasion assay detecting the invasion ability of lung adenocarcinoma cells/lewis-ifn-y-+spd-1.png]

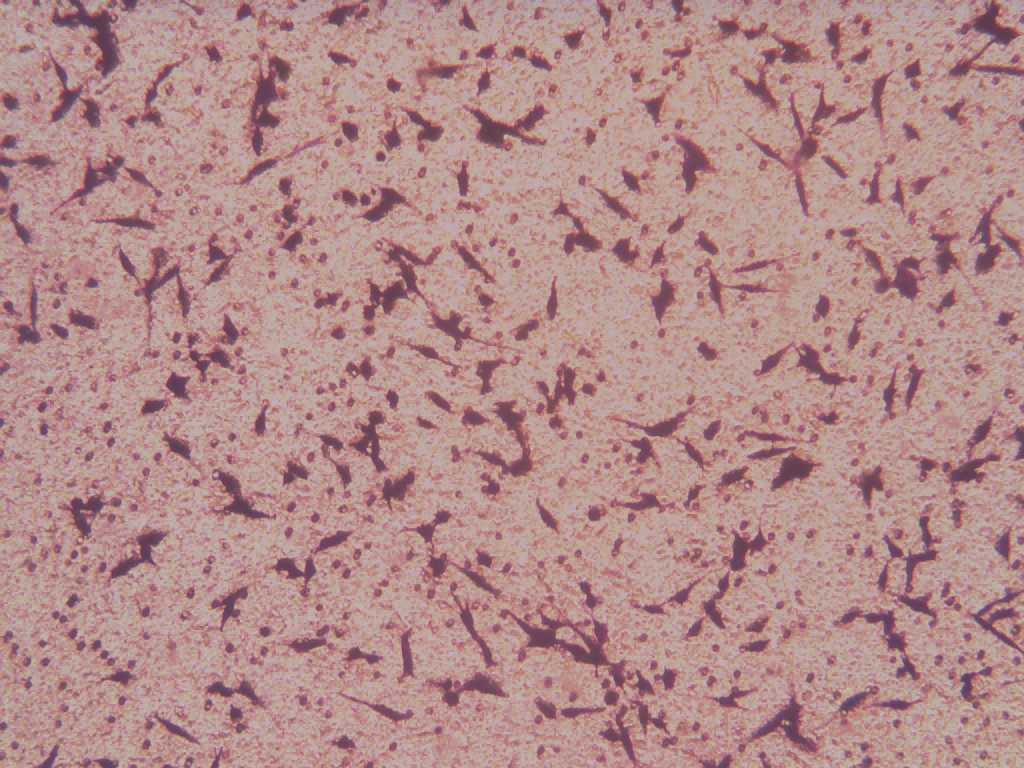

Supplement: Supplementary file 5 [file DataSheet5.zip › Transwell cell invasion assay detecting the invasion ability of lung adenocarcinoma cells/lewis-ifn-y.png]

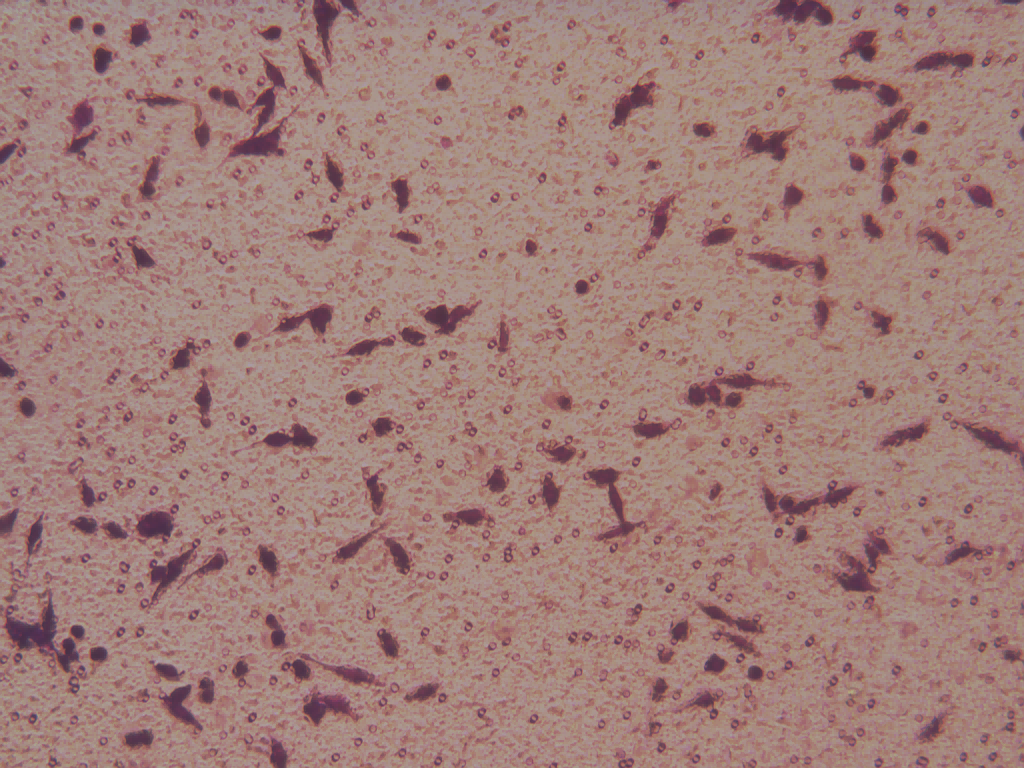

Supplement: Supplementary file 5 [file DataSheet5.zip › Transwell cell invasion assay detecting the invasion ability of lung adenocarcinoma cells/lewis-spd-1.png]

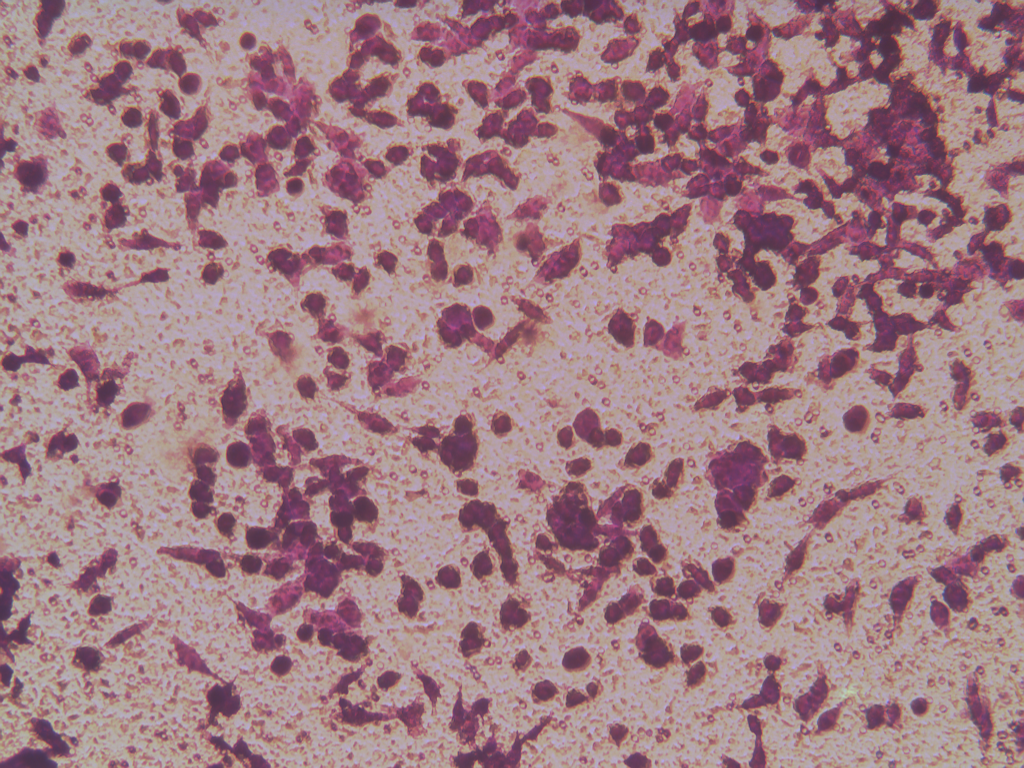

Supplement: Supplementary file 5 [file DataSheet5.zip › Transwell cell invasion assay detecting the invasion ability of lung adenocarcinoma cells/lewis-vector.tif]

KEBAO\_model-2023\_12\_12\_18\_18\_24.fcs

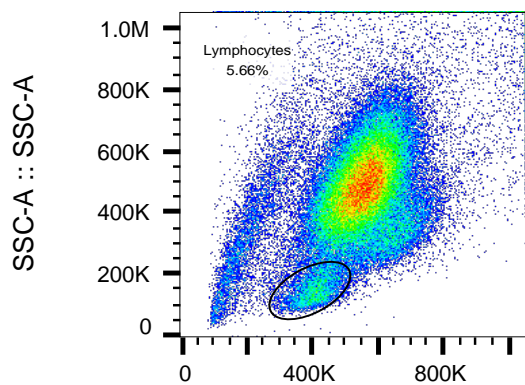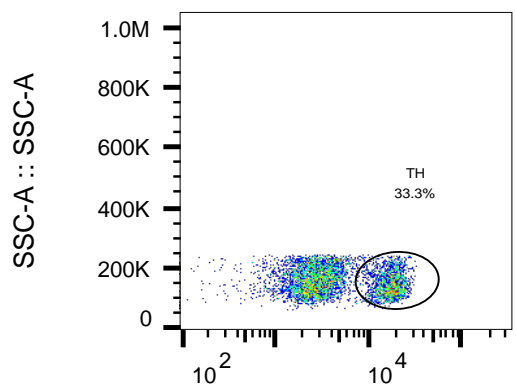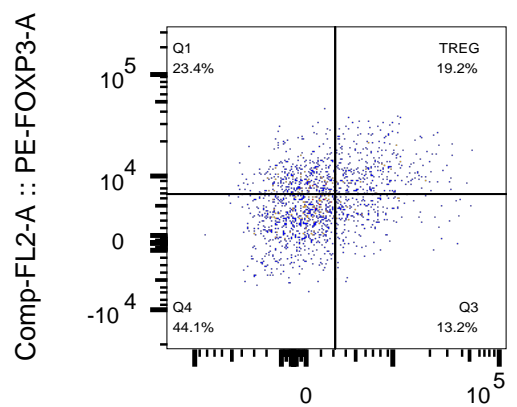

KEBAO\_vector\_2023\_12\_12\_18\_18\_24.fcs

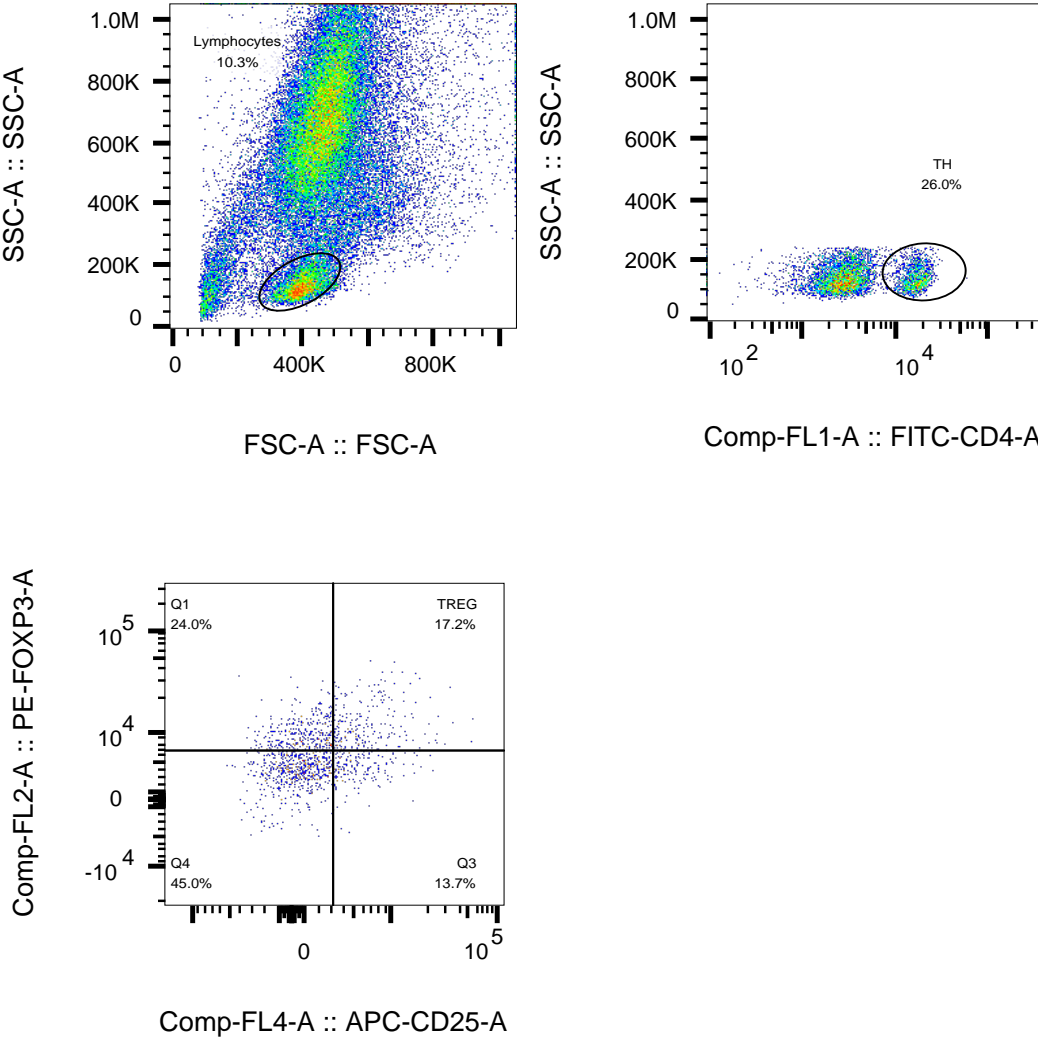

KEBAO\_spd-1\_2023\_12\_12\_18\_18\_24.fcs

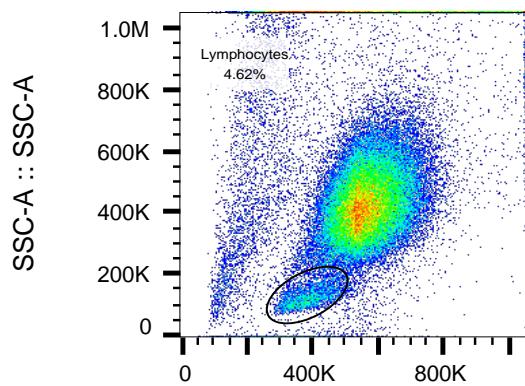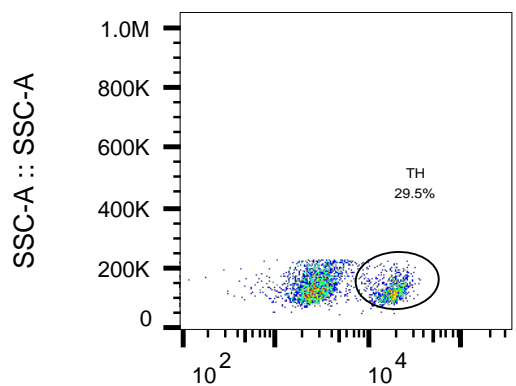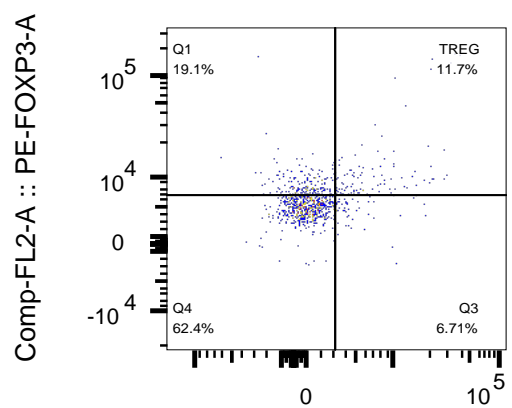

KEBAO\_ifn-y\_2023\_12\_12\_18\_18\_24.fcs

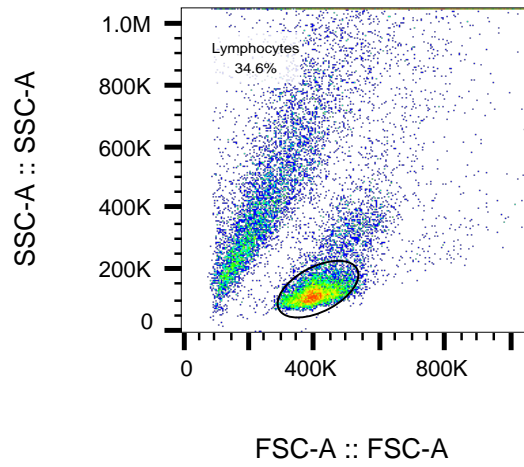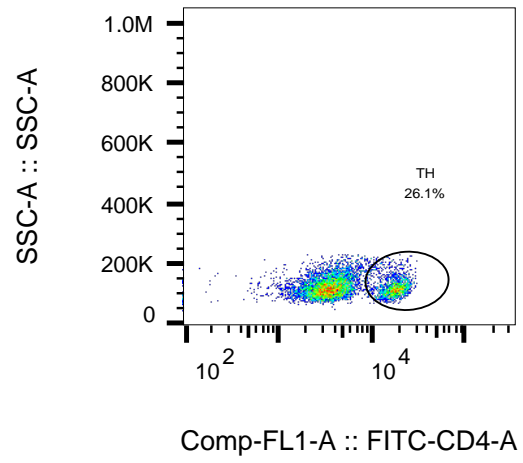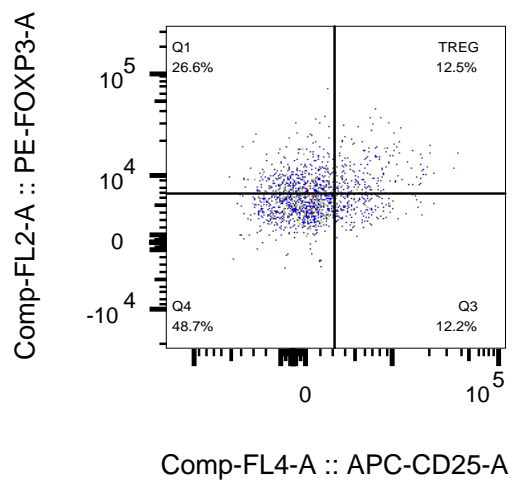

KEBAO\_ifn-y+spd-1\_2023\_12\_12\_18\_18\_24.fcs

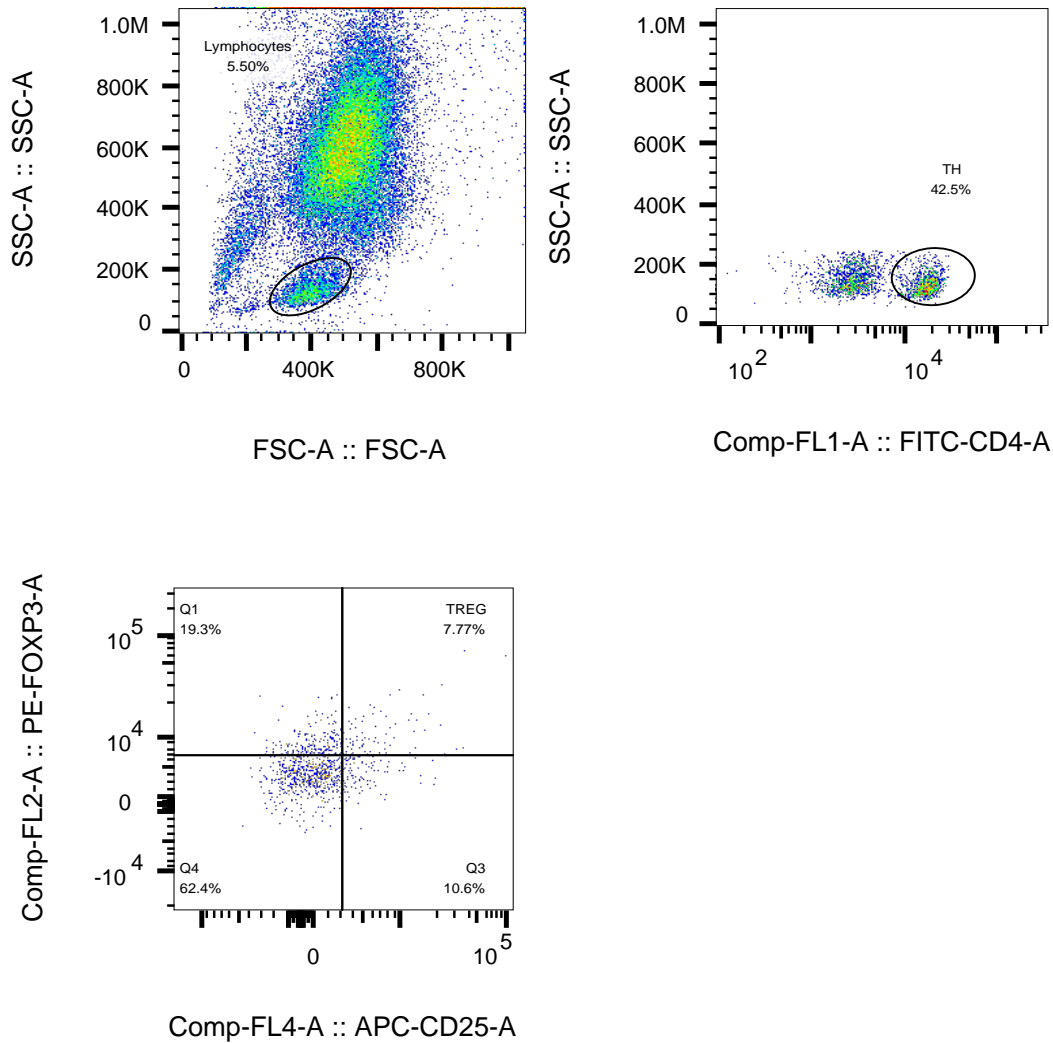

Supplement: Supplementary file 6 [file DataSheet6.zip › Identification of surface markers/TREG/12-dec-2023-TREG.pdf]

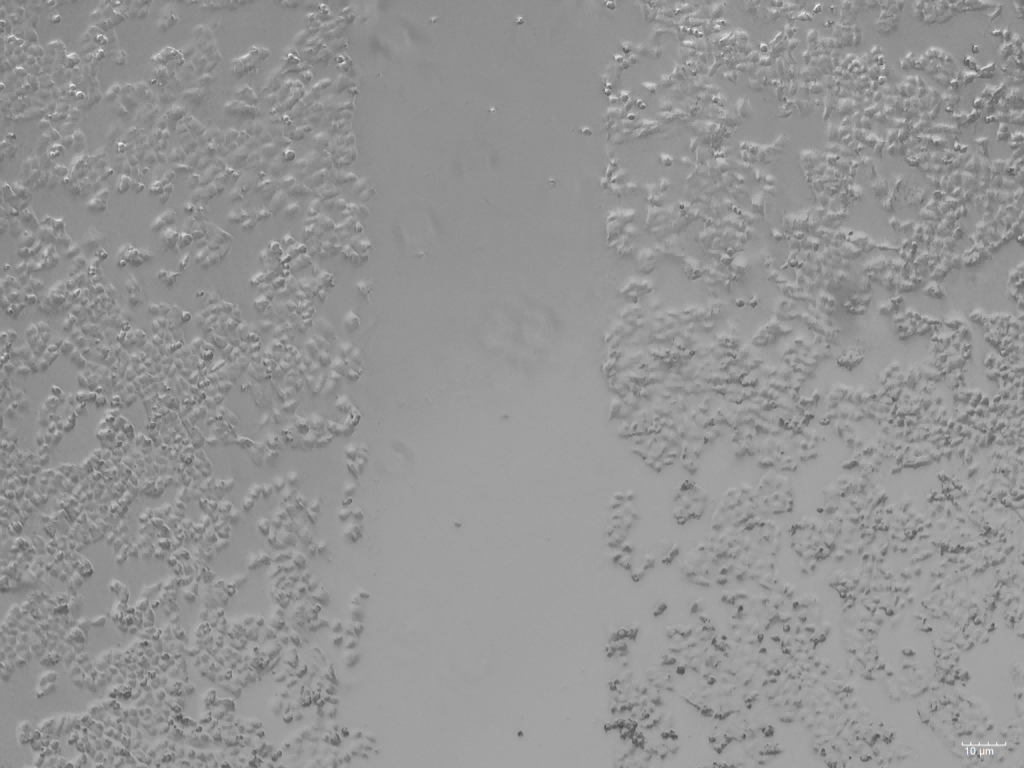

Supplement: Supplementary file 7 [file DataSheet7.zip › Scratch assay/A549-Control-0.png]

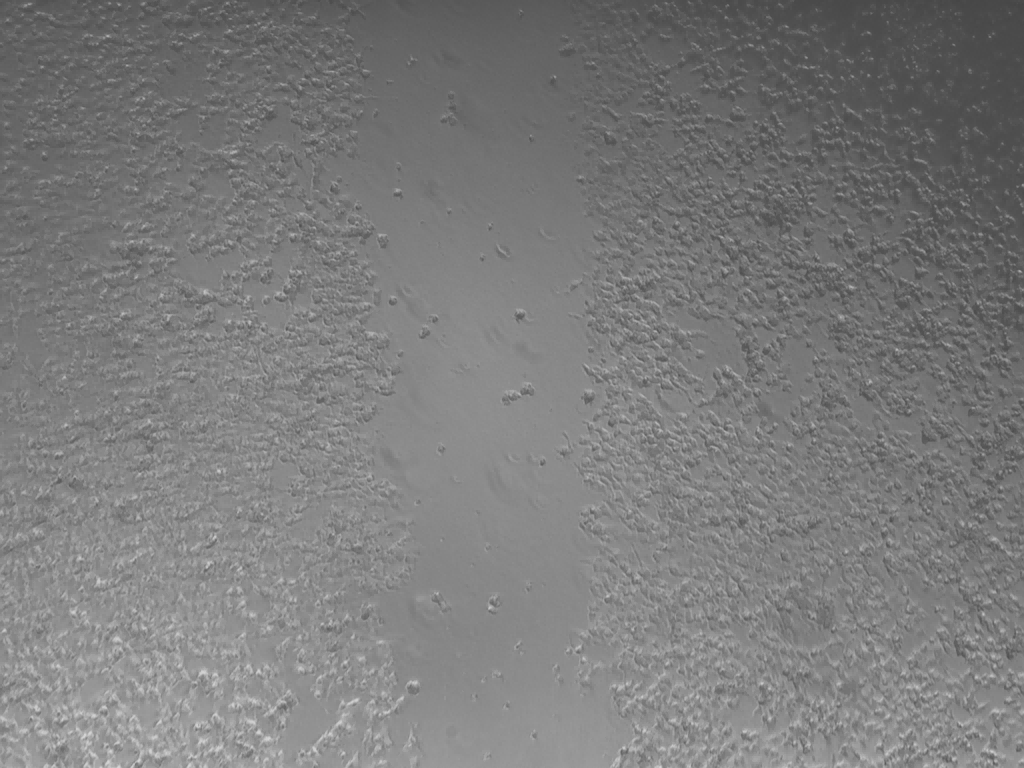

Supplement: Supplementary file 7 [file DataSheet7.zip › Scratch assay/A549-Control-36.png]

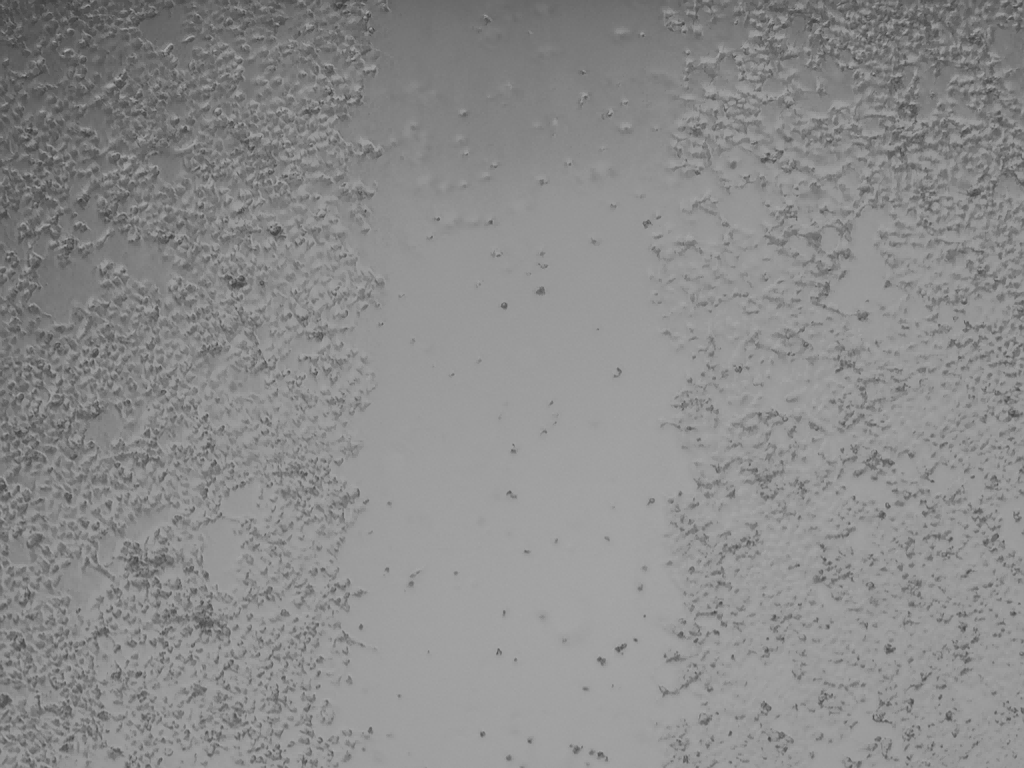

Supplement: Supplementary file 7 [file DataSheet7.zip › Scratch assay/A549-IFN-y+sPD-1-0.png]

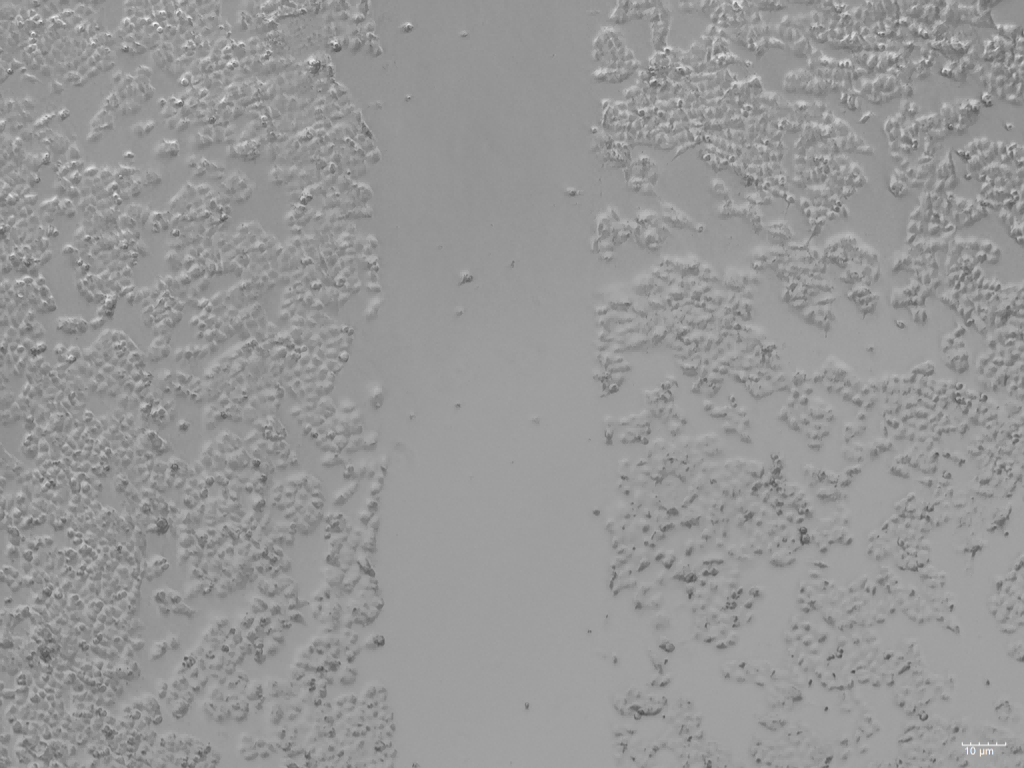

Supplement: Supplementary file 7 [file DataSheet7.zip › Scratch assay/A549-IFN-y+sPD-1-36.png]

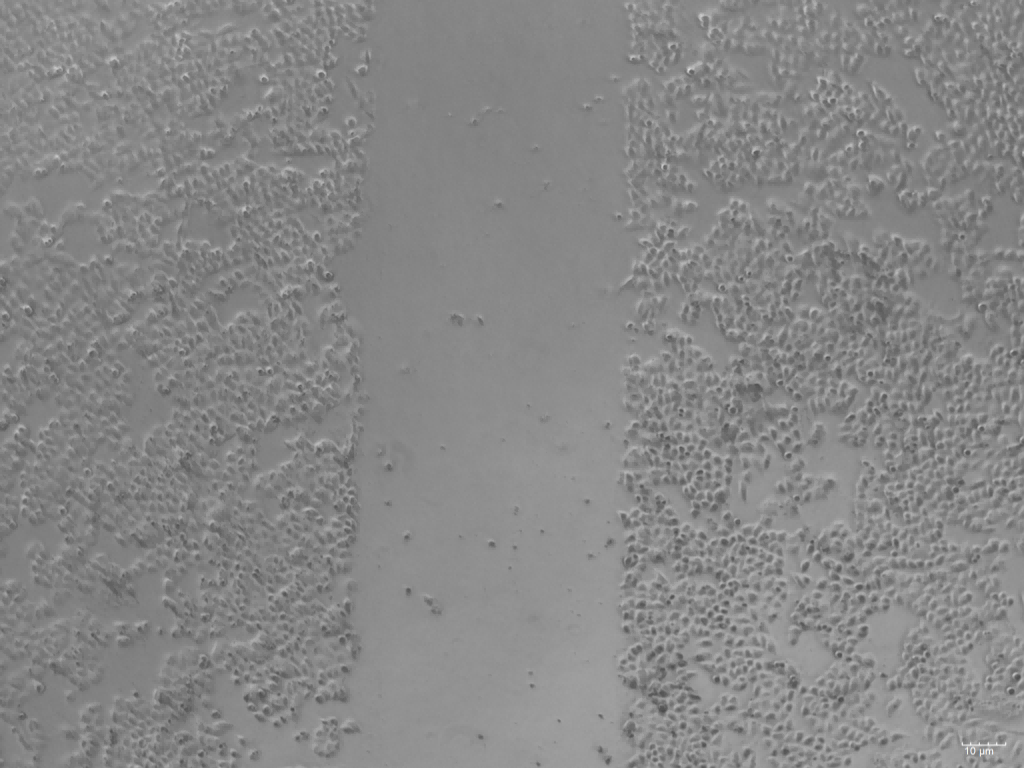

Supplement: Supplementary file 7 [file DataSheet7.zip › Scratch assay/A549-IFN-y-0.png]

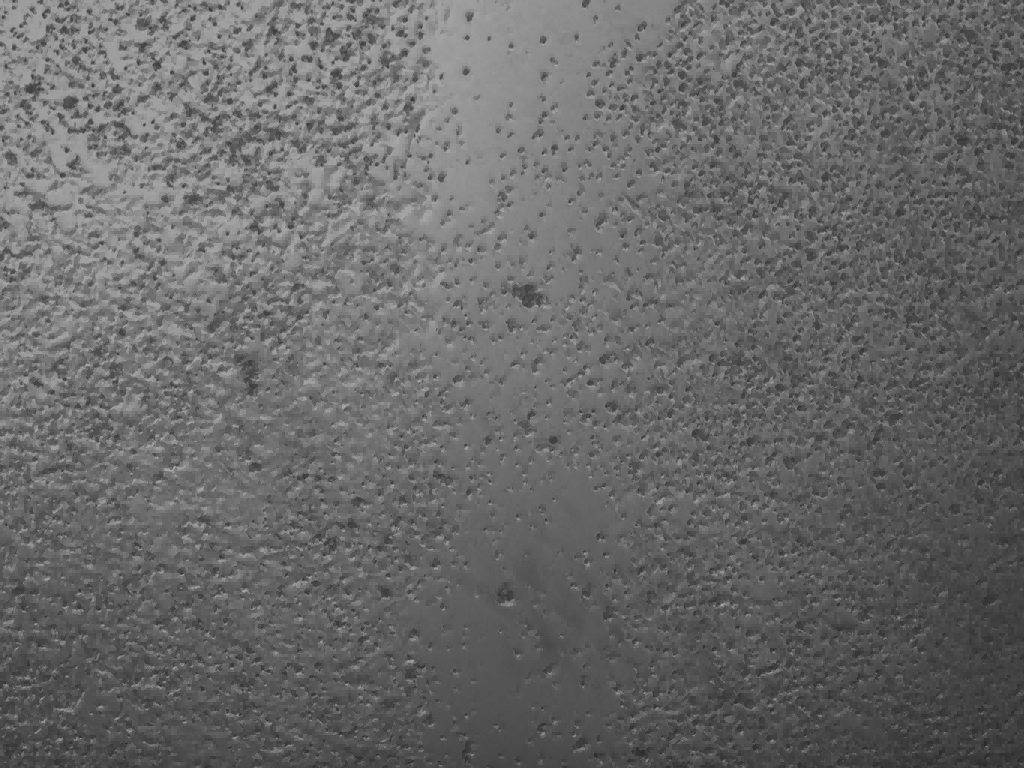

Supplement: Supplementary file 7 [file DataSheet7.zip › Scratch assay/A549-IFN-y-36.png]

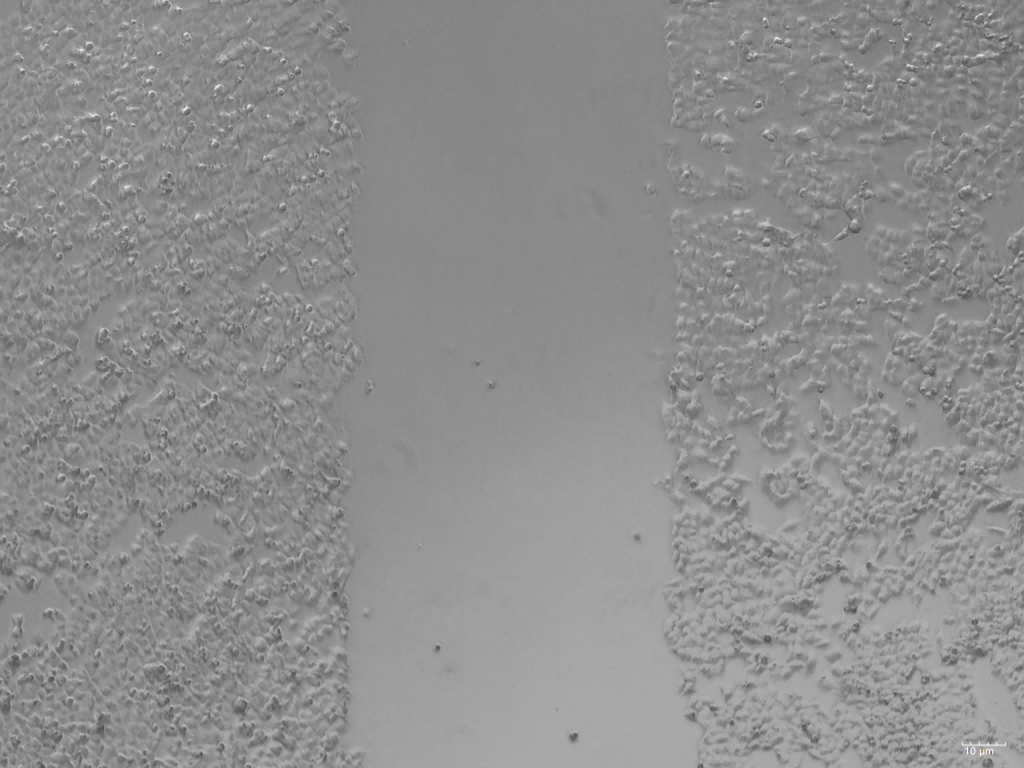

Supplement: Supplementary file 7 [file DataSheet7.zip › Scratch assay/A549-sPD-1-0.png]

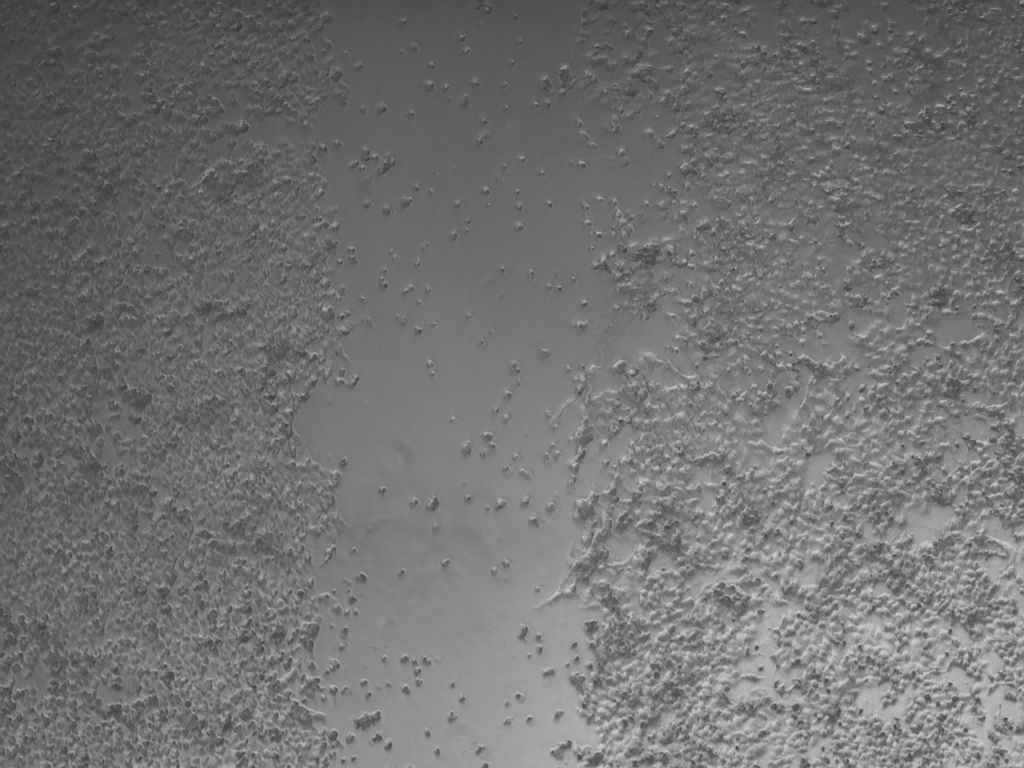

Supplement: Supplementary file 7 [file DataSheet7.zip › Scratch assay/A549-sPD-1-36.png]

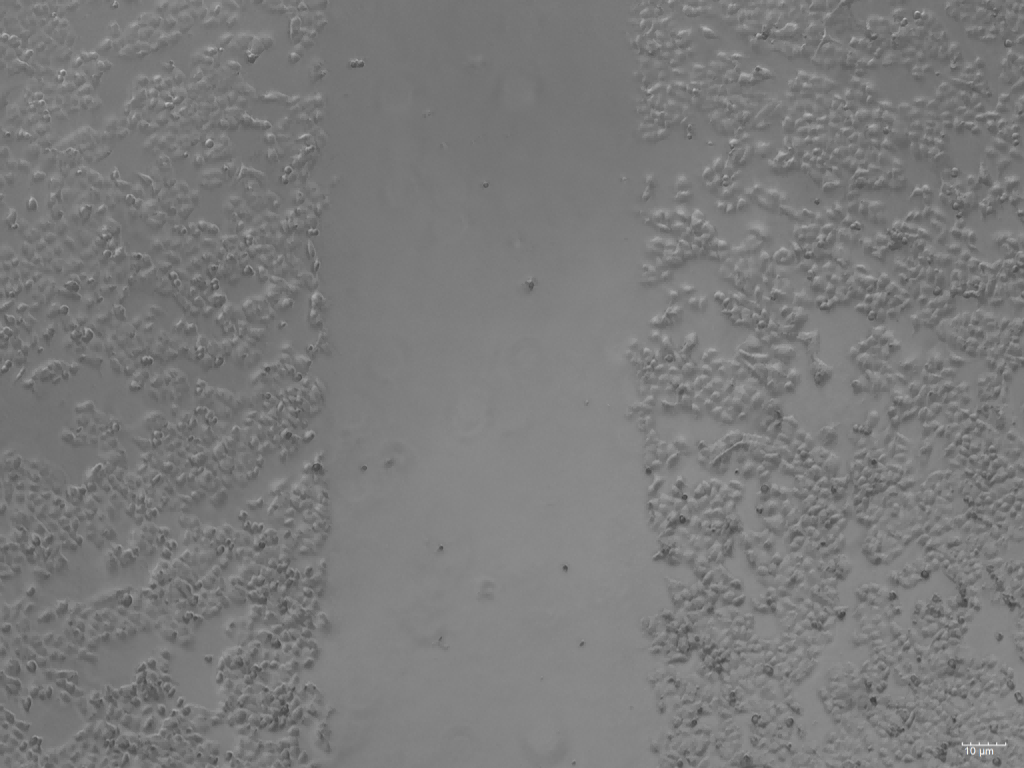

Supplement: Supplementary file 7 [file DataSheet7.zip › Scratch assay/A549-Vector-0.png]

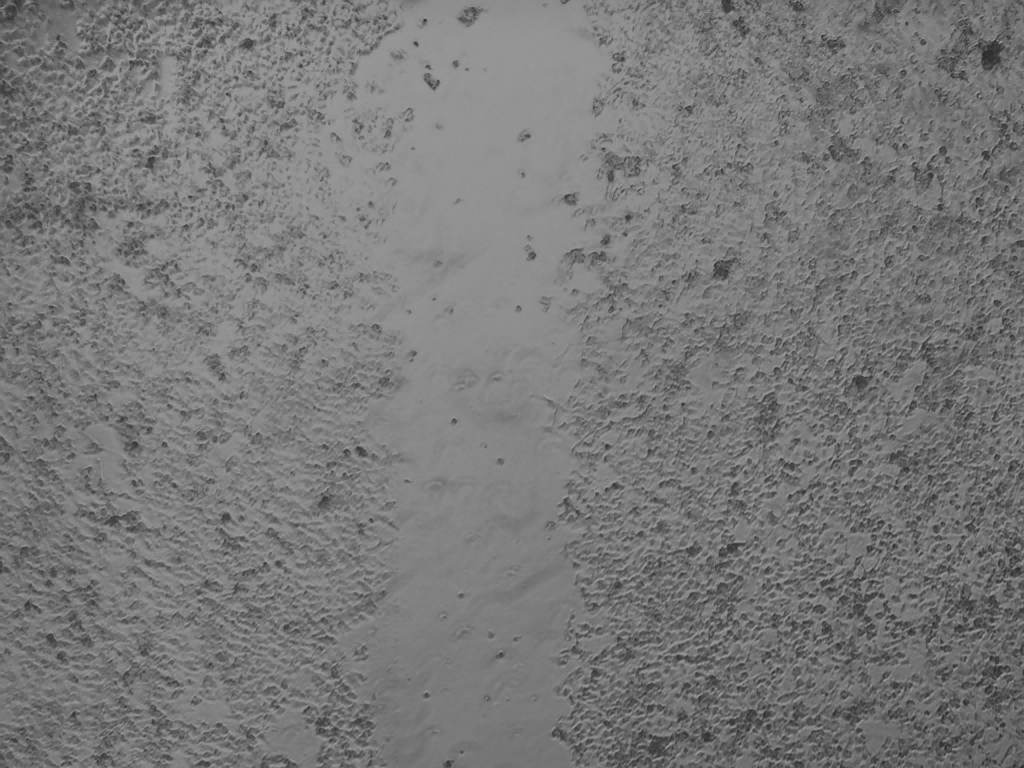

Supplement: Supplementary file 7 [file DataSheet7.zip › Scratch assay/A549-Vector-36.png]

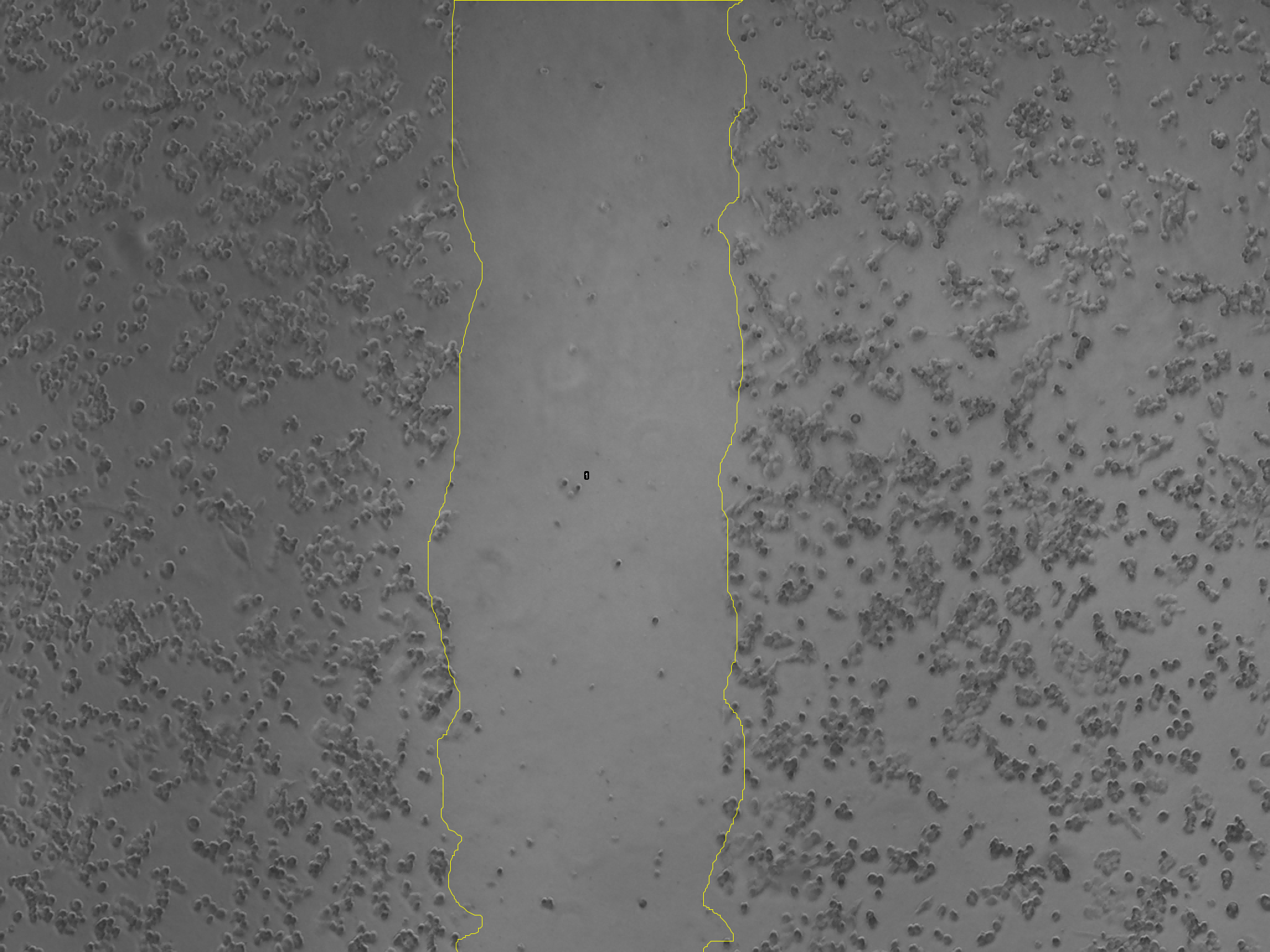

Supplement: Supplementary file 7 [file DataSheet7.zip › Scratch assay/lewis-Control-0.png]

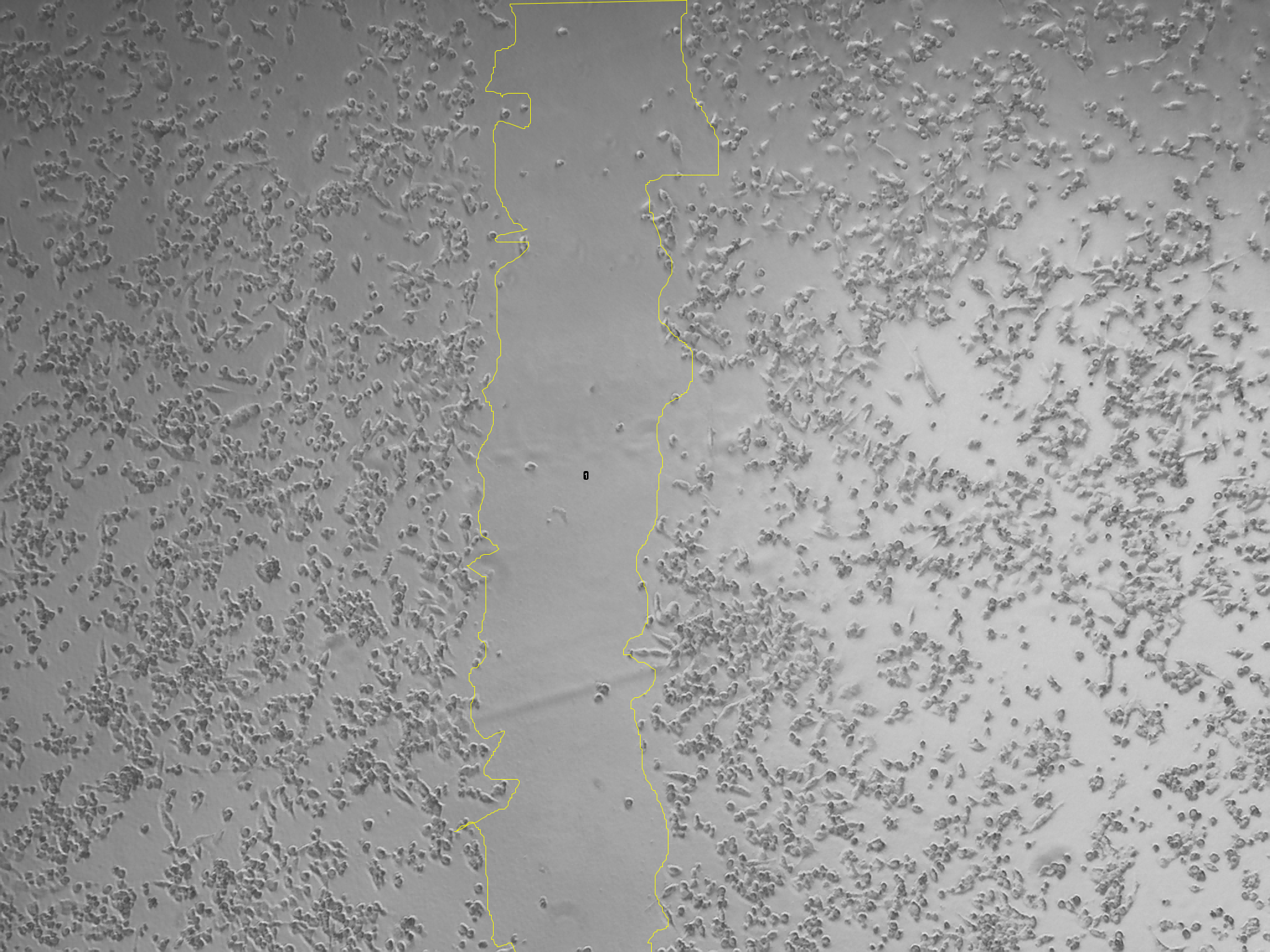

Supplement: Supplementary file 7 [file DataSheet7.zip › Scratch assay/lewis-Control-36.png]

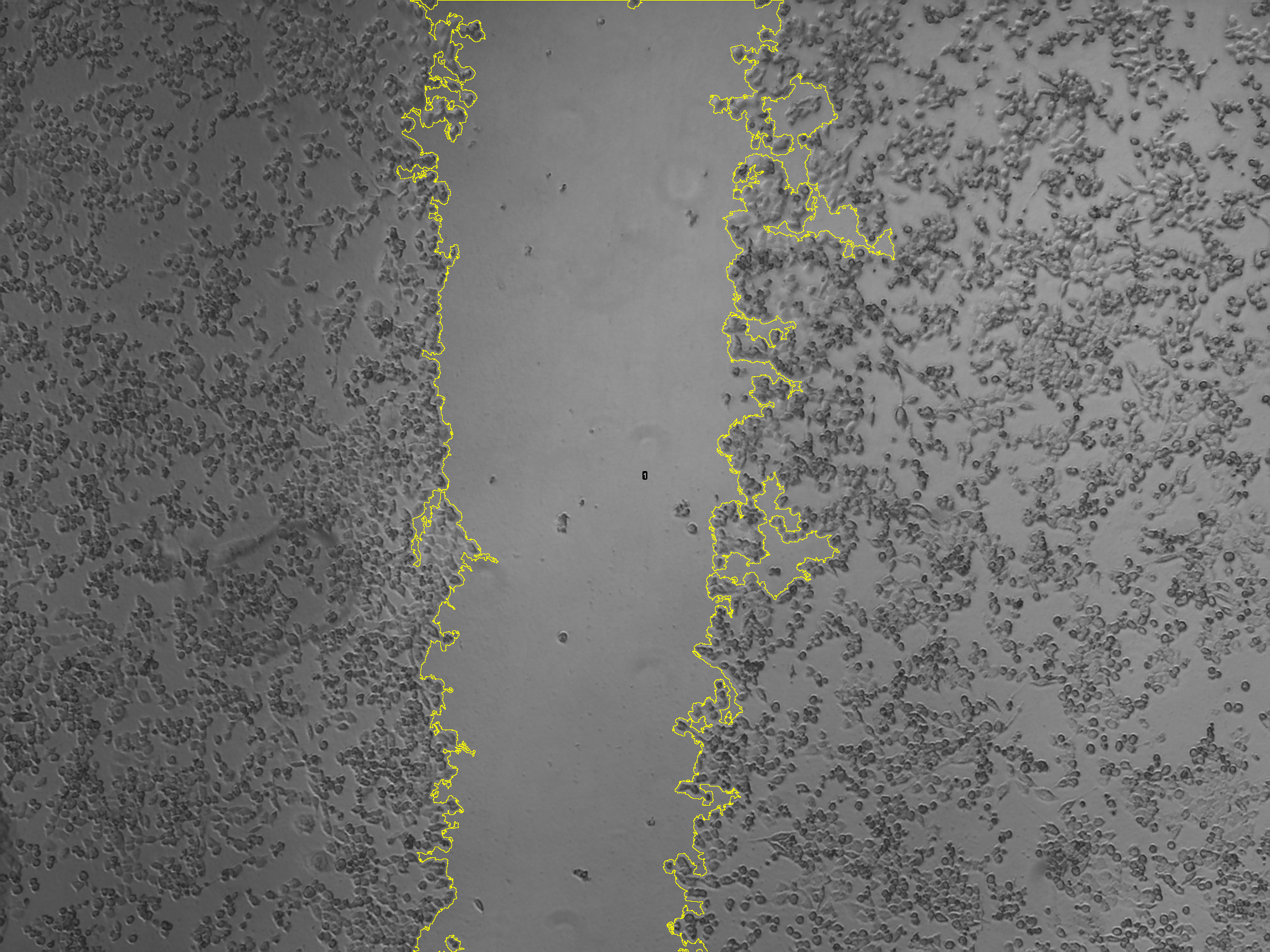

Supplement: Supplementary file 7 [file DataSheet7.zip › Scratch assay/lewis-IFN-y+sPD-1-0.png]

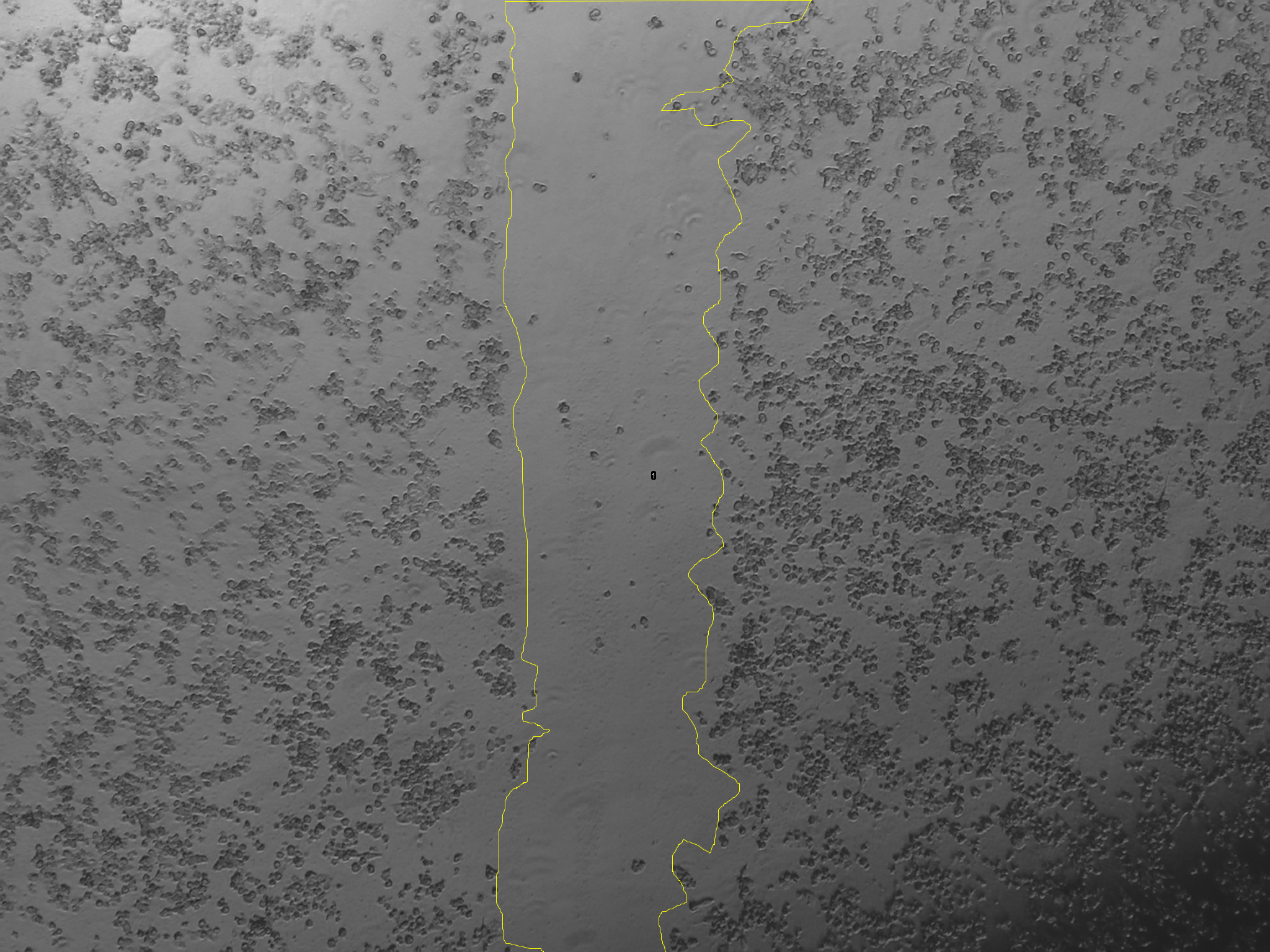

Supplement: Supplementary file 7 [file DataSheet7.zip › Scratch assay/lewis-IFN-y+sPD-1-36.png]

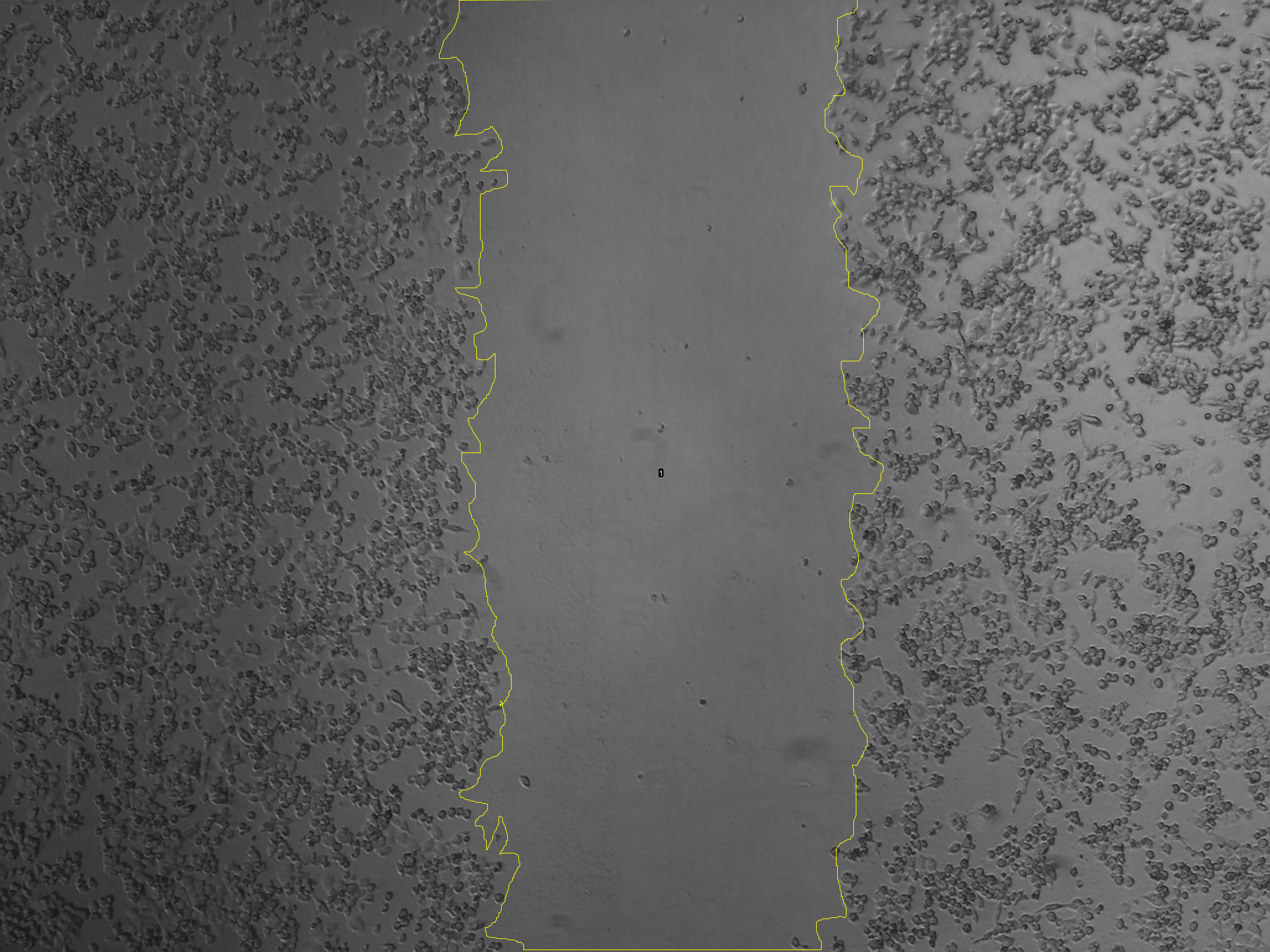

Supplement: Supplementary file 7 [file DataSheet7.zip › Scratch assay/lewis-IFN-y-0.png]

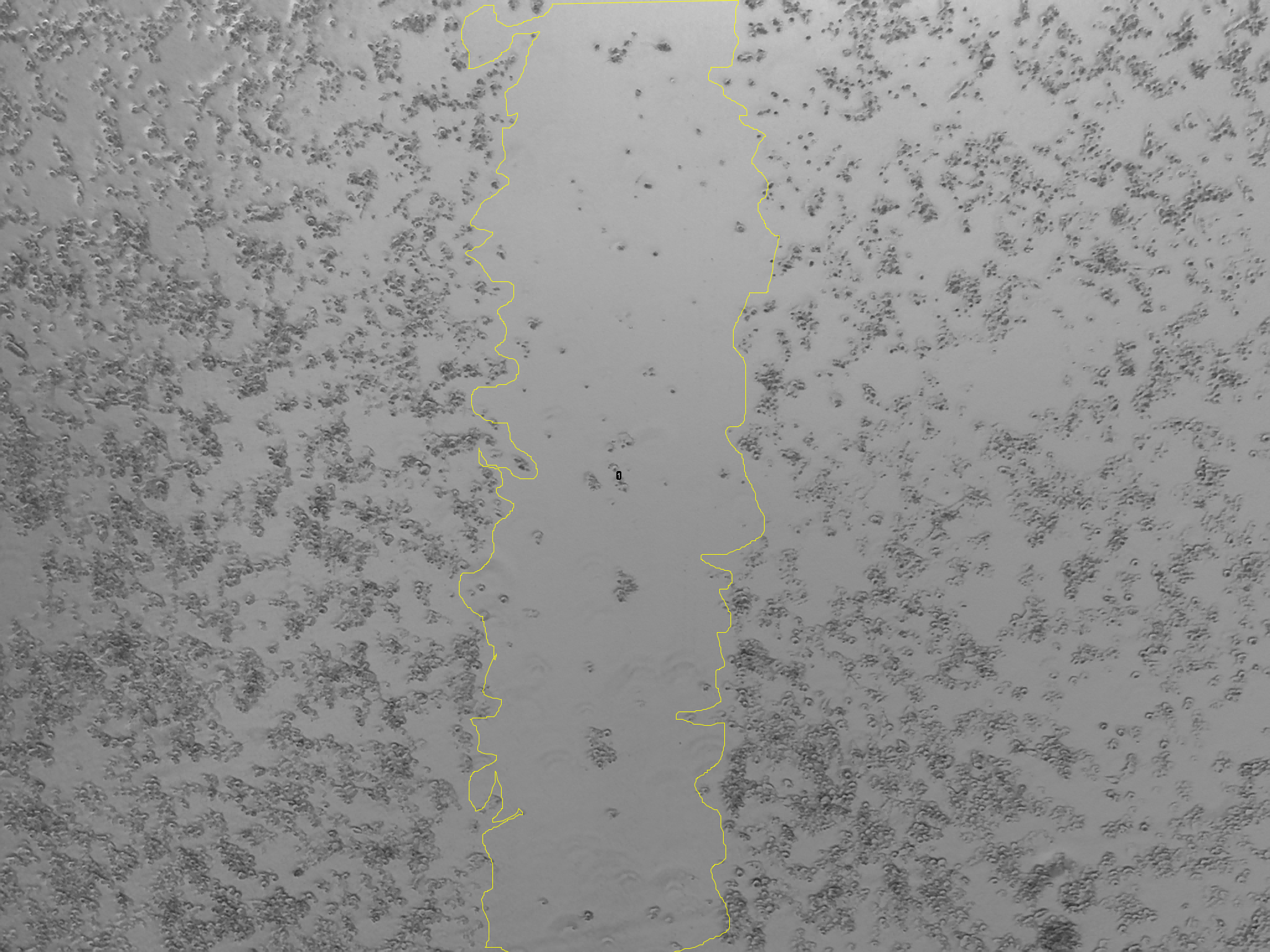

Supplement: Supplementary file 7 [file DataSheet7.zip › Scratch assay/lewis-IFN-y-36.png]

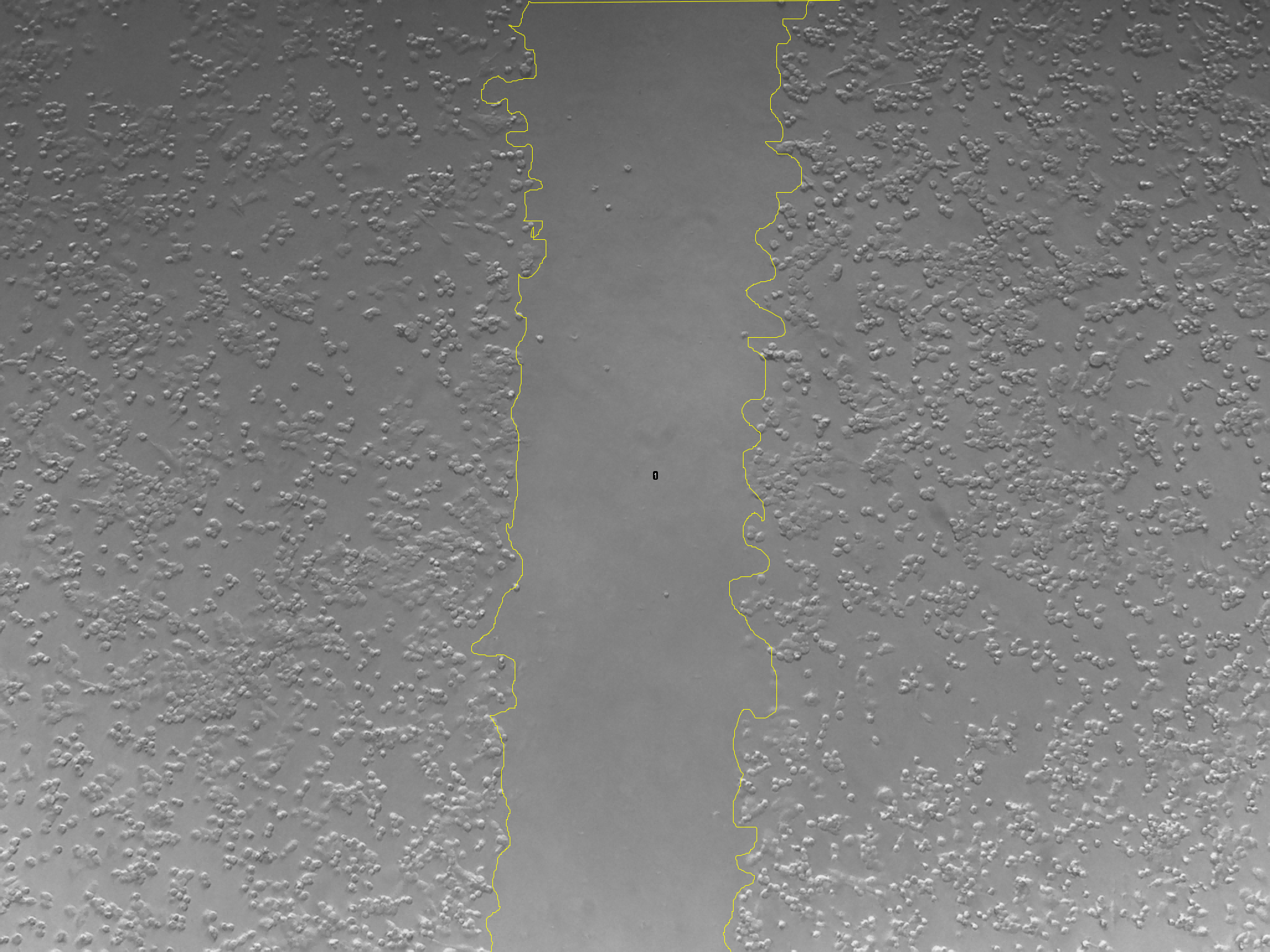

Supplement: Supplementary file 7 [file DataSheet7.zip › Scratch assay/lewis-sPD-1-0.png]

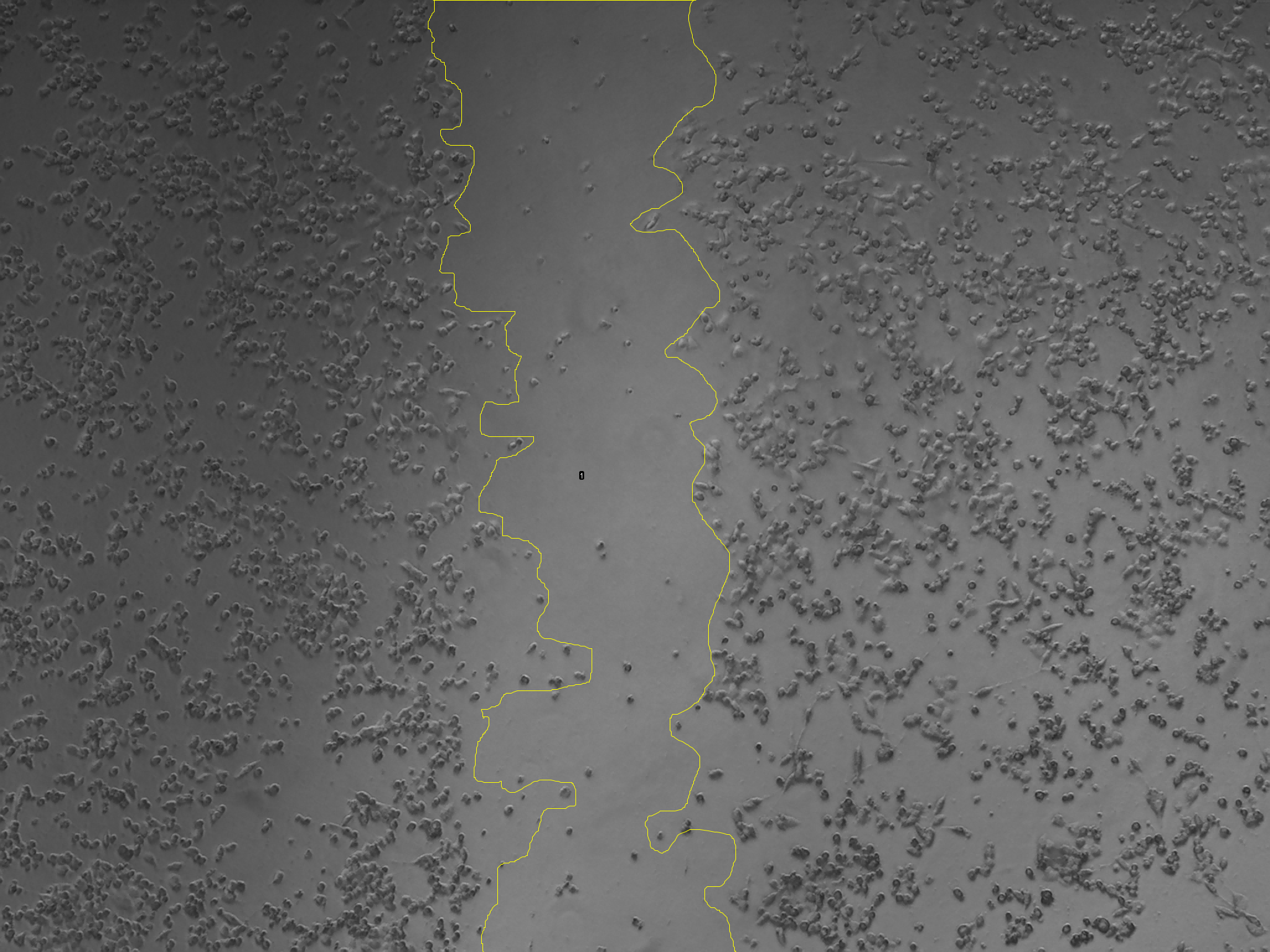

Supplement: Supplementary file 7 [file DataSheet7.zip › Scratch assay/lewis-sPD-1-36.png]

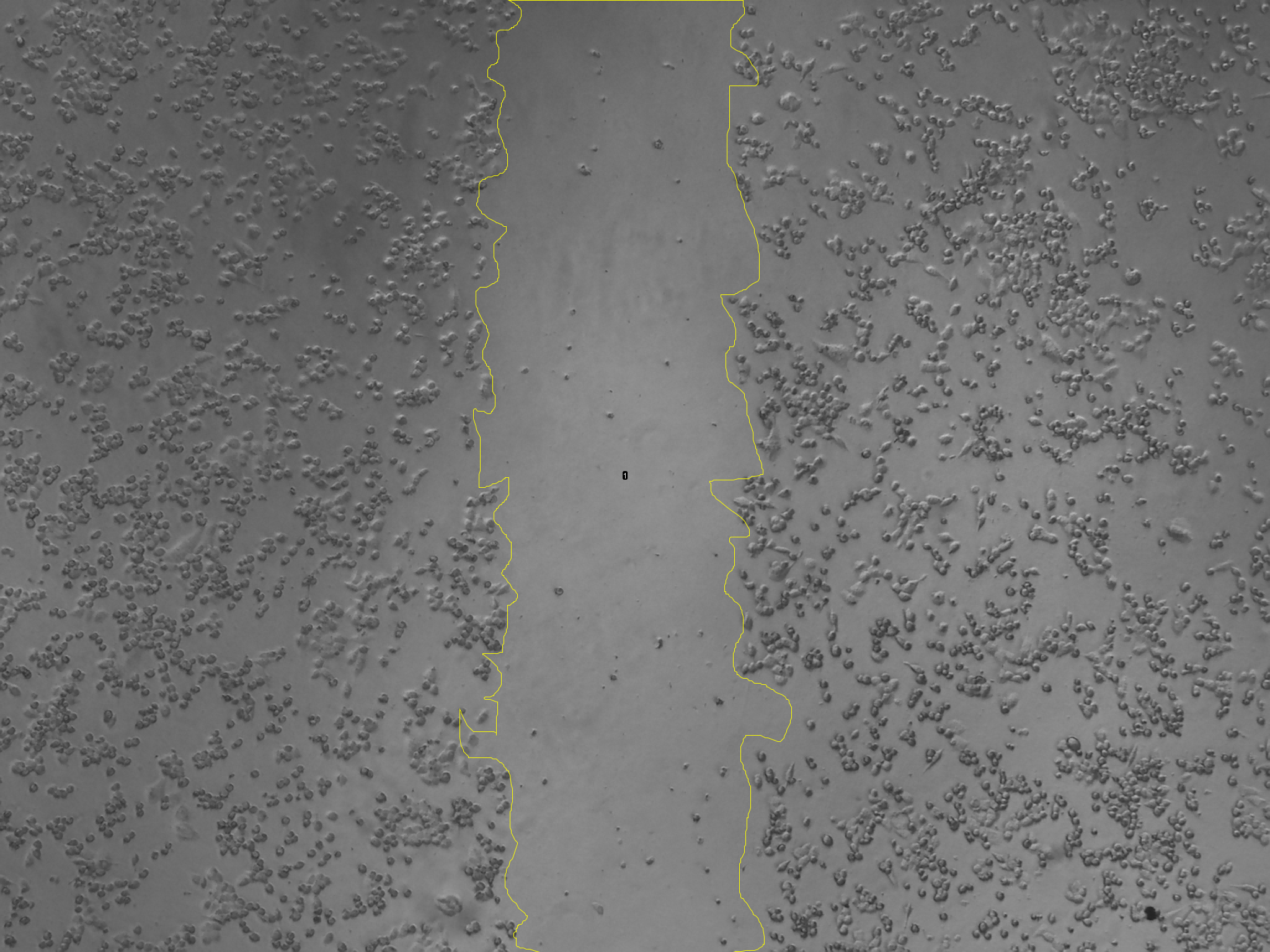

Supplement: Supplementary file 7 [file DataSheet7.zip › Scratch assay/lewis-Vector-0.png]

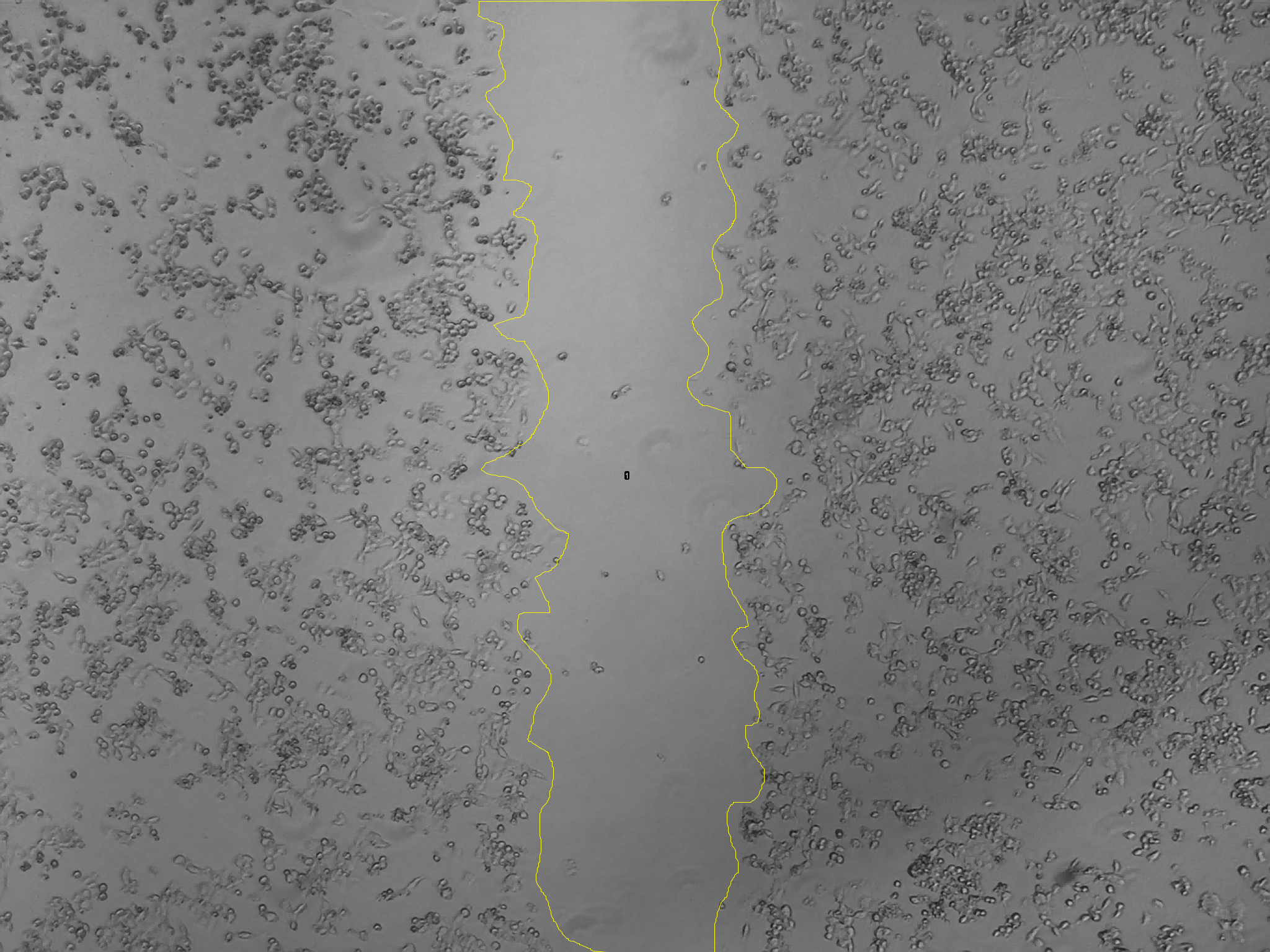

Supplement: Supplementary file 7 [file DataSheet7.zip › Scratch assay/lewis-Vector-36.png]

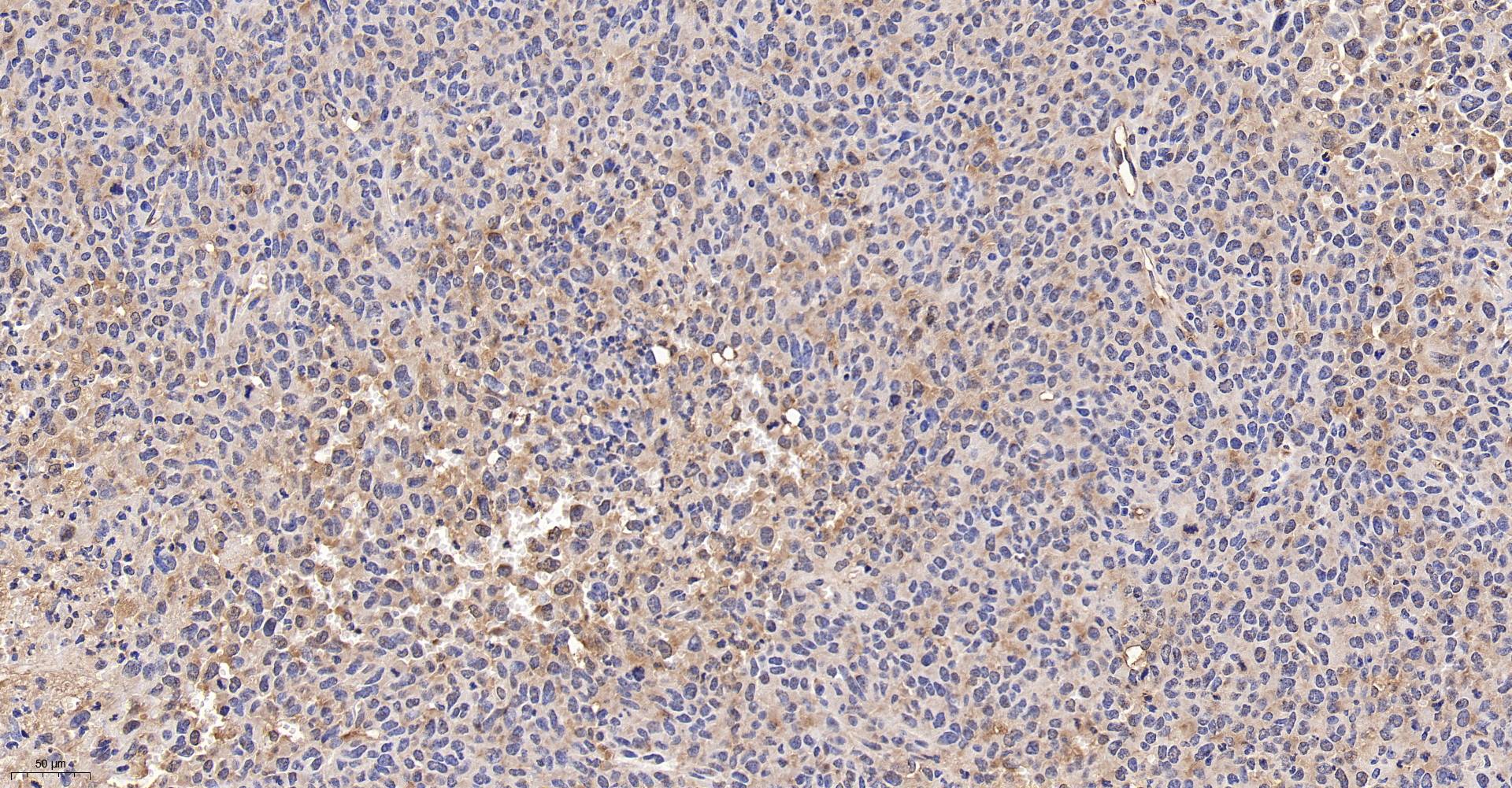

Supplement: Supplementary file 8 [file DataSheet8.zip › Expression of Bax and Bcl-2 in tumor tissue of each group/AKT/IFN-γ AKT_20.0x(1).jpg]

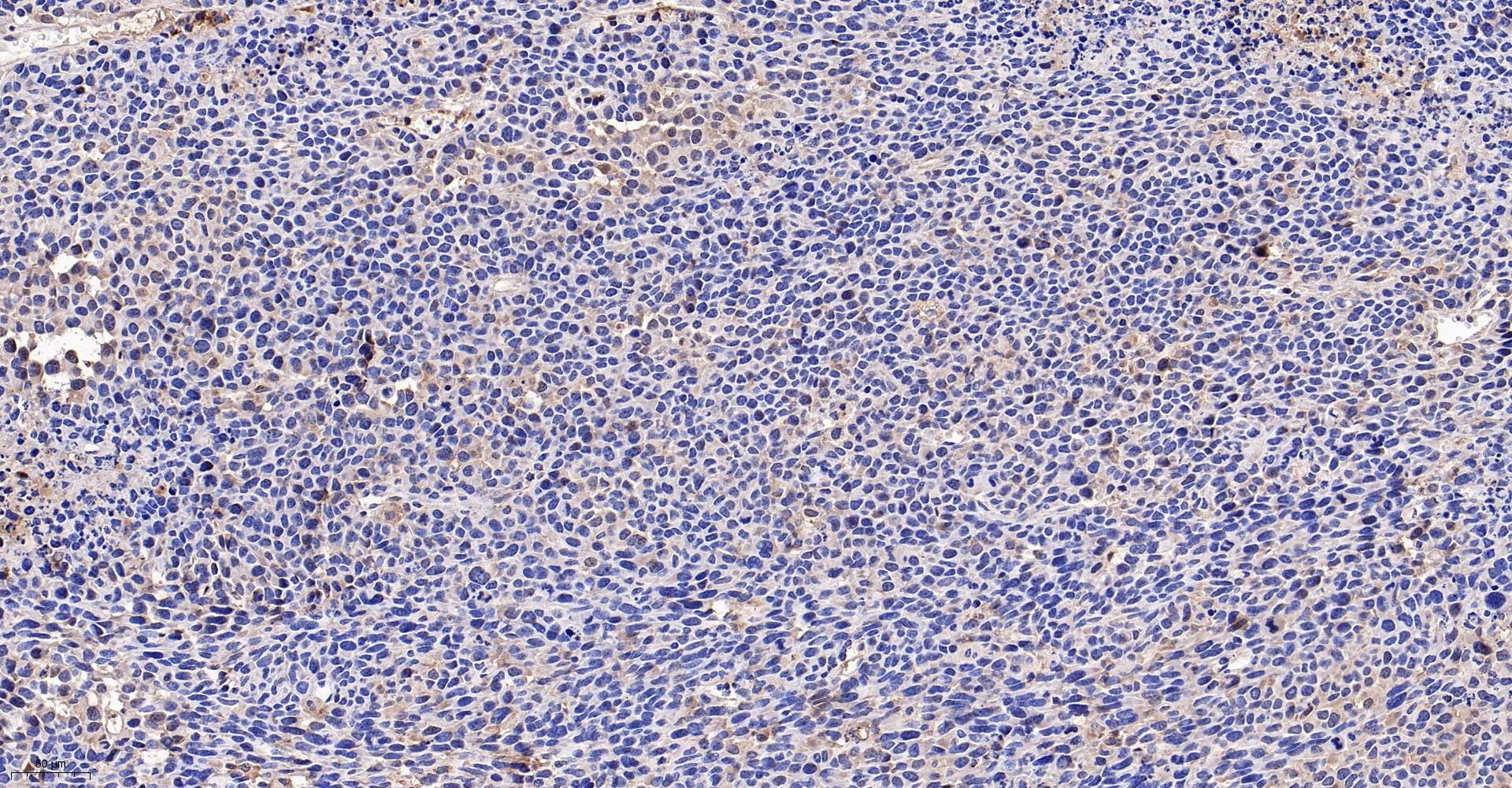

Supplement: Supplementary file 8 [file DataSheet8.zip › Expression of Bax and Bcl-2 in tumor tissue of each group/AKT/IFN-γ+sPD-1 AKT_20.0x(1).jpg]

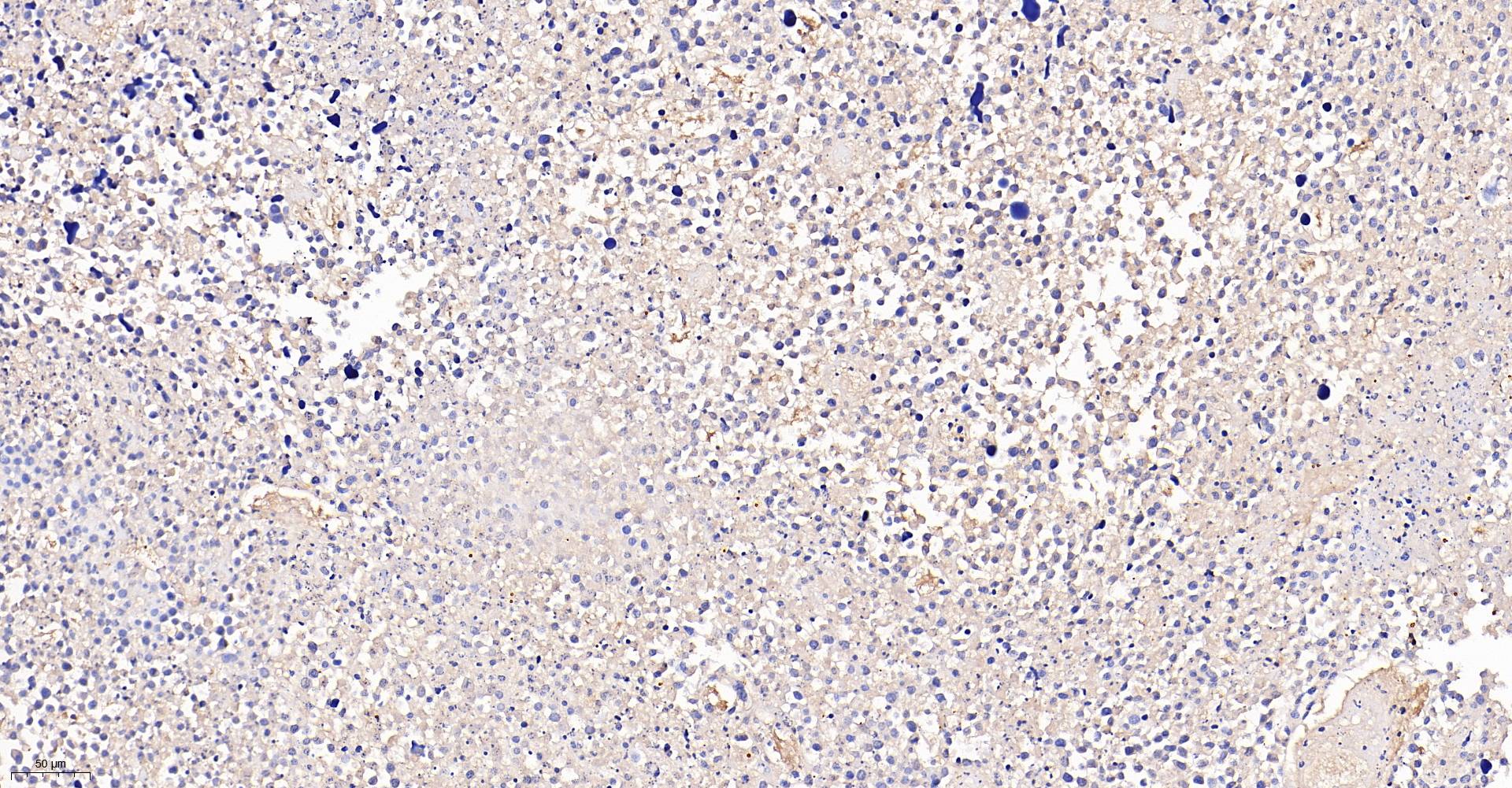

Supplement: Supplementary file 8 [file DataSheet8.zip › Expression of Bax and Bcl-2 in tumor tissue of each group/AKT/Model AKT_20.0x(1).jpg]

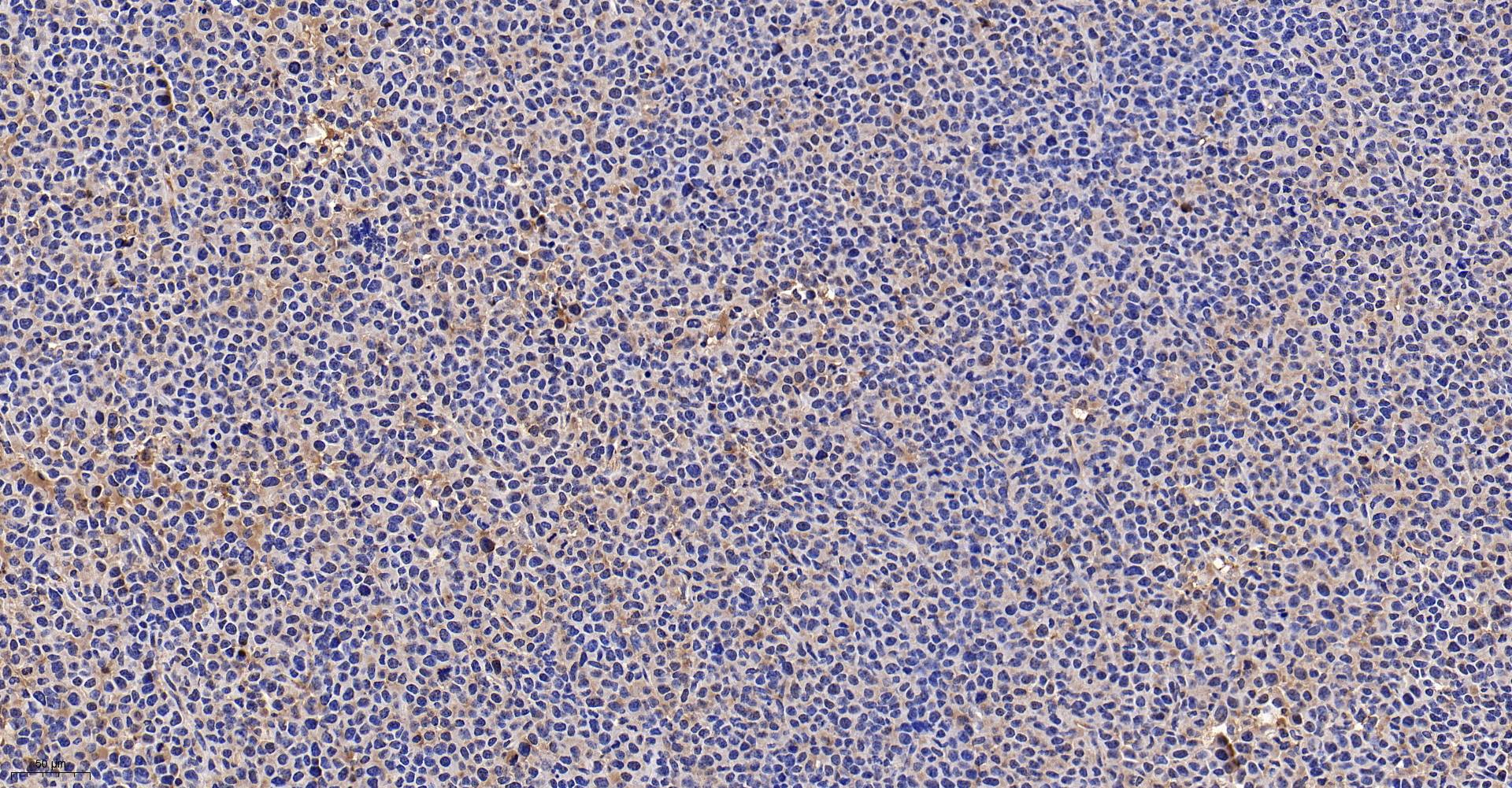

Supplement: Supplementary file 8 [file DataSheet8.zip › Expression of Bax and Bcl-2 in tumor tissue of each group/AKT/sPD-1 AKT_20.0x(1).jpg]

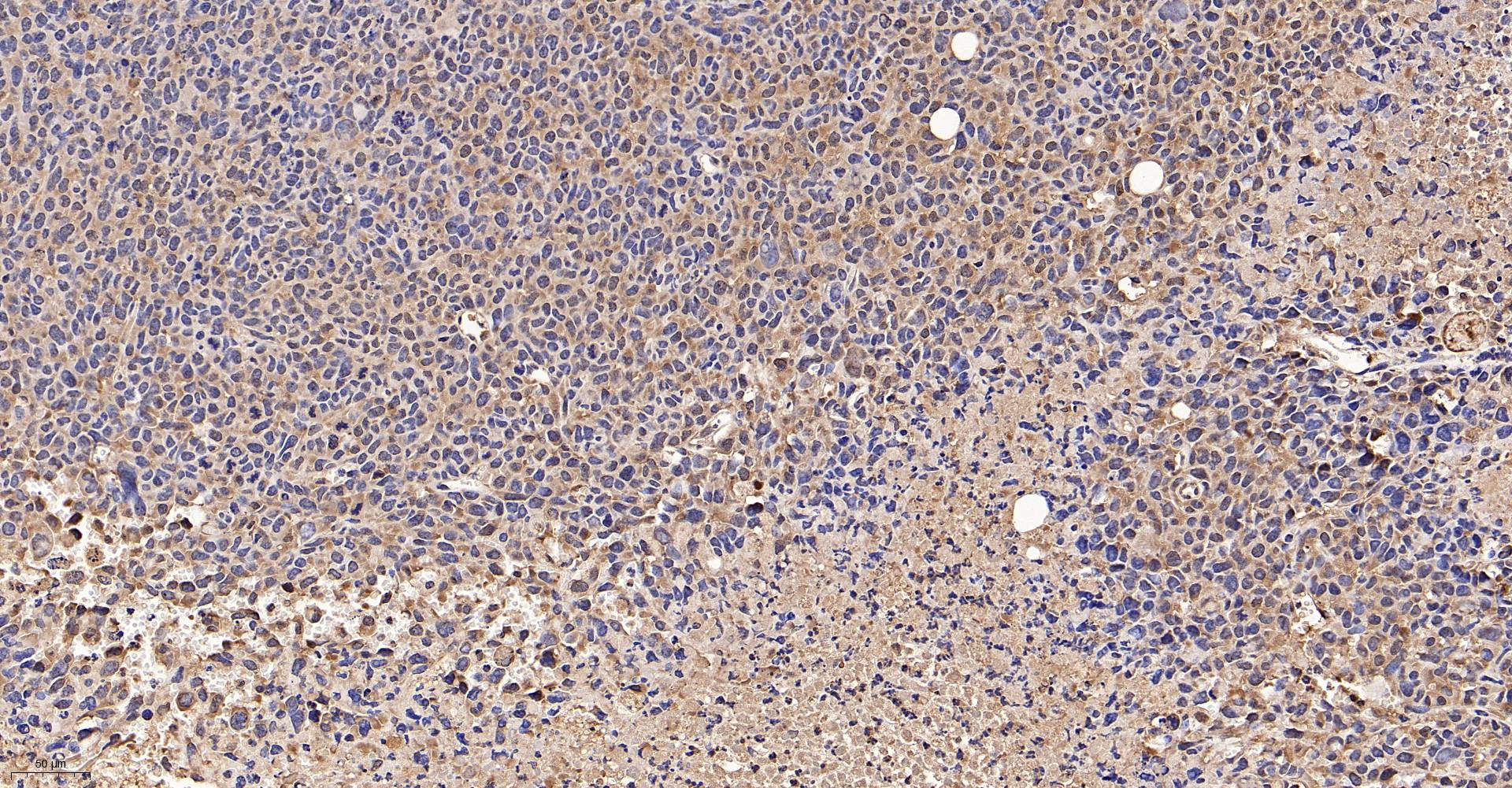

Supplement: Supplementary file 8 [file DataSheet8.zip › Expression of Bax and Bcl-2 in tumor tissue of each group/AKT/Vector AKT_20.0x(1).jpg]

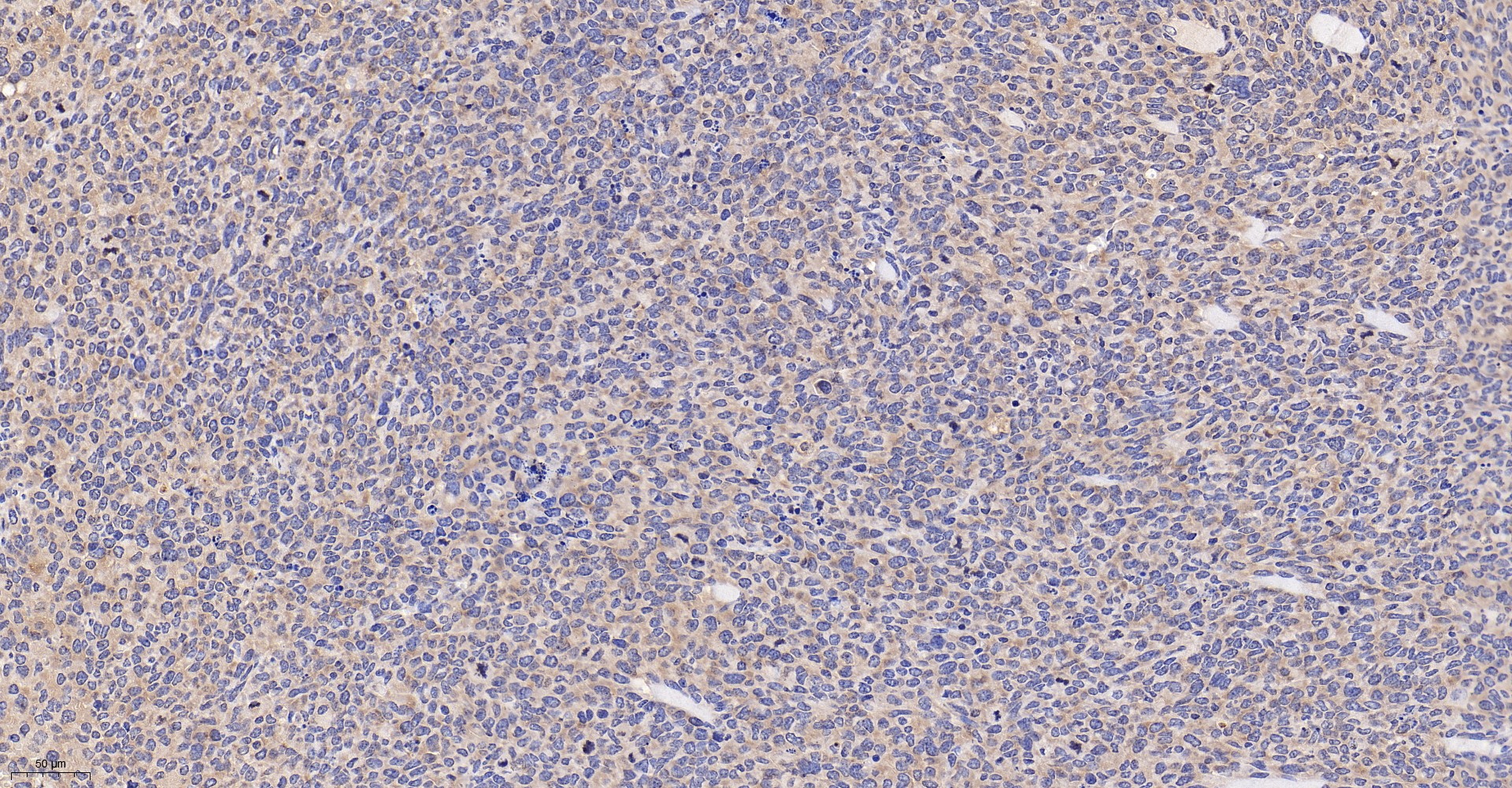

Supplement: Supplementary file 8 [file DataSheet8.zip › Expression of Bax and Bcl-2 in tumor tissue of each group/Bax/IFN-γ BAX_20.0x.jpg]

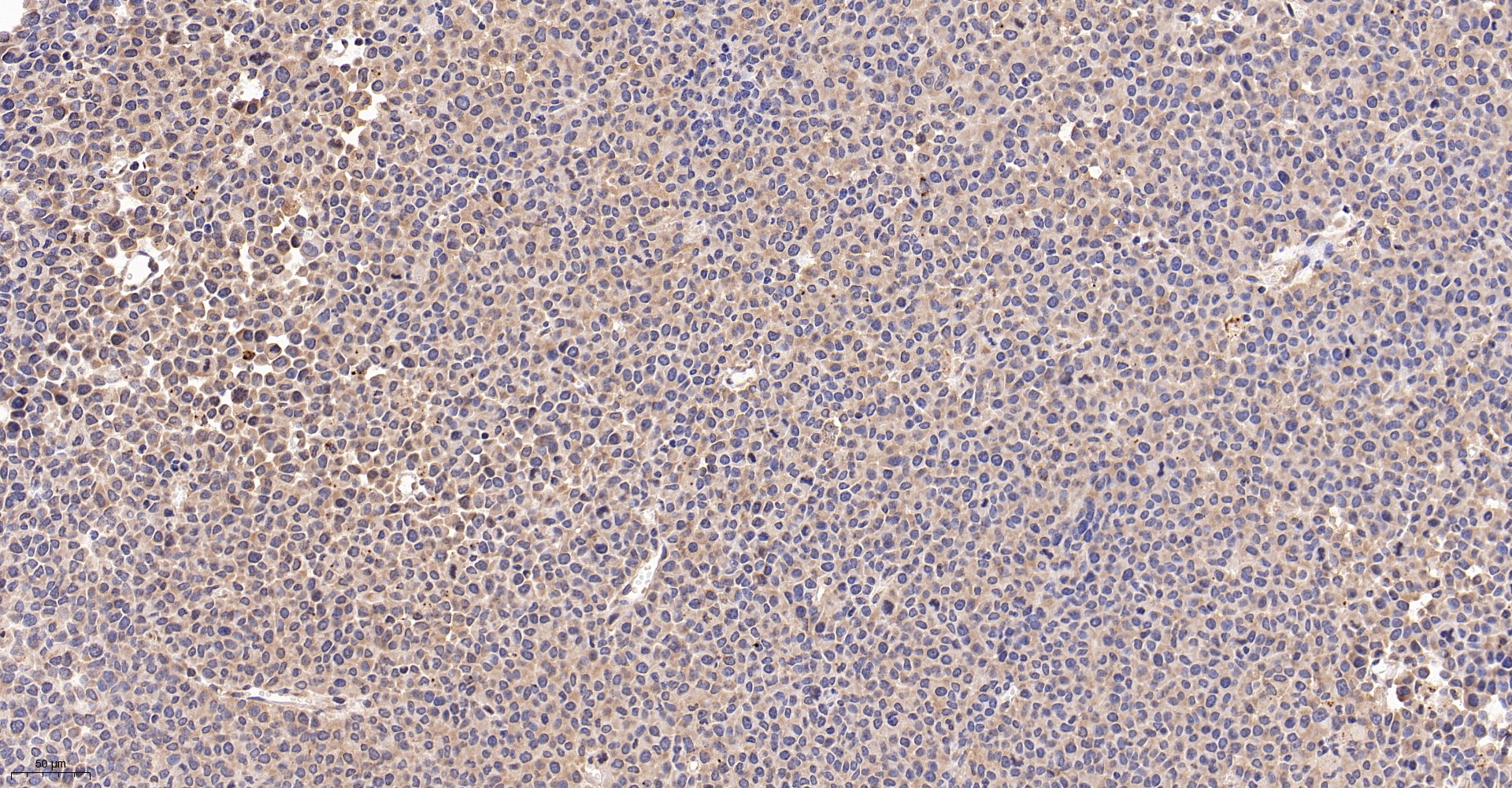

Supplement: Supplementary file 8 [file DataSheet8.zip › Expression of Bax and Bcl-2 in tumor tissue of each group/Bax/IFN-γ+sPD-1 BAX_20.0x.jpg]

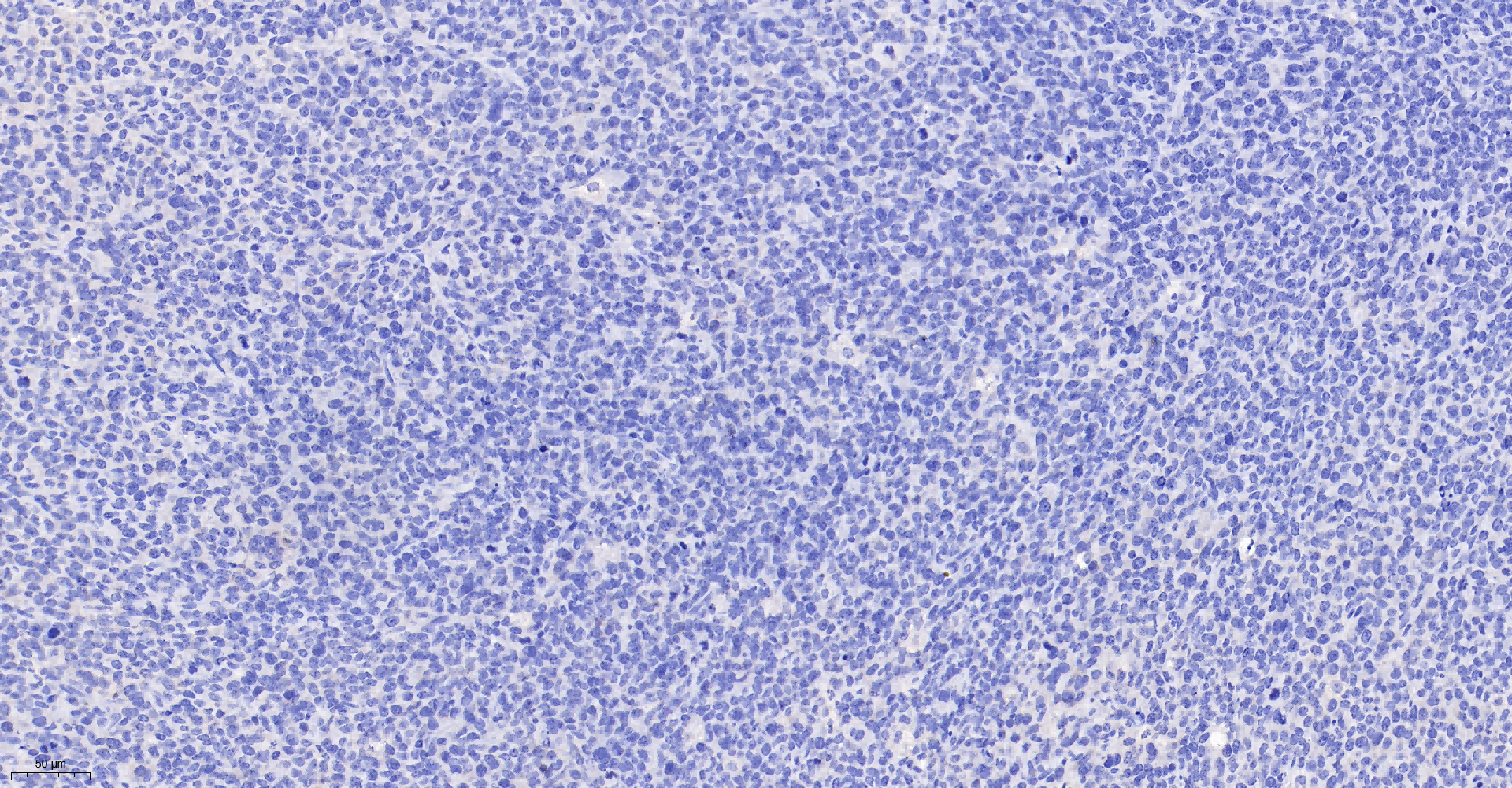

Supplement: Supplementary file 8 [file DataSheet8.zip › Expression of Bax and Bcl-2 in tumor tissue of each group/Bax/Model- BAX_20.0x.png]

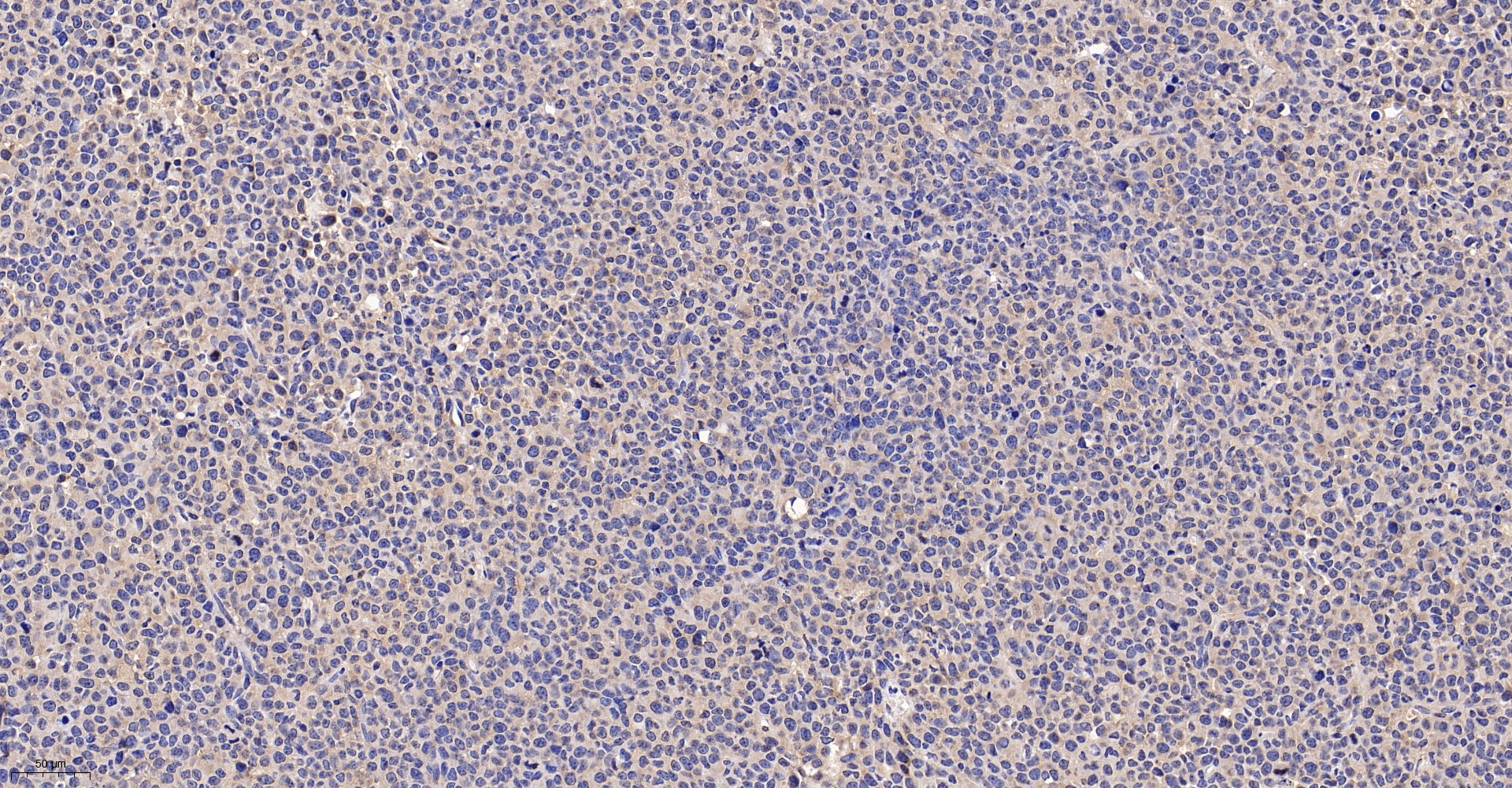

Supplement: Supplementary file 8 [file DataSheet8.zip › Expression of Bax and Bcl-2 in tumor tissue of each group/Bax/sPD-1 BAX_20.0x.jpg]

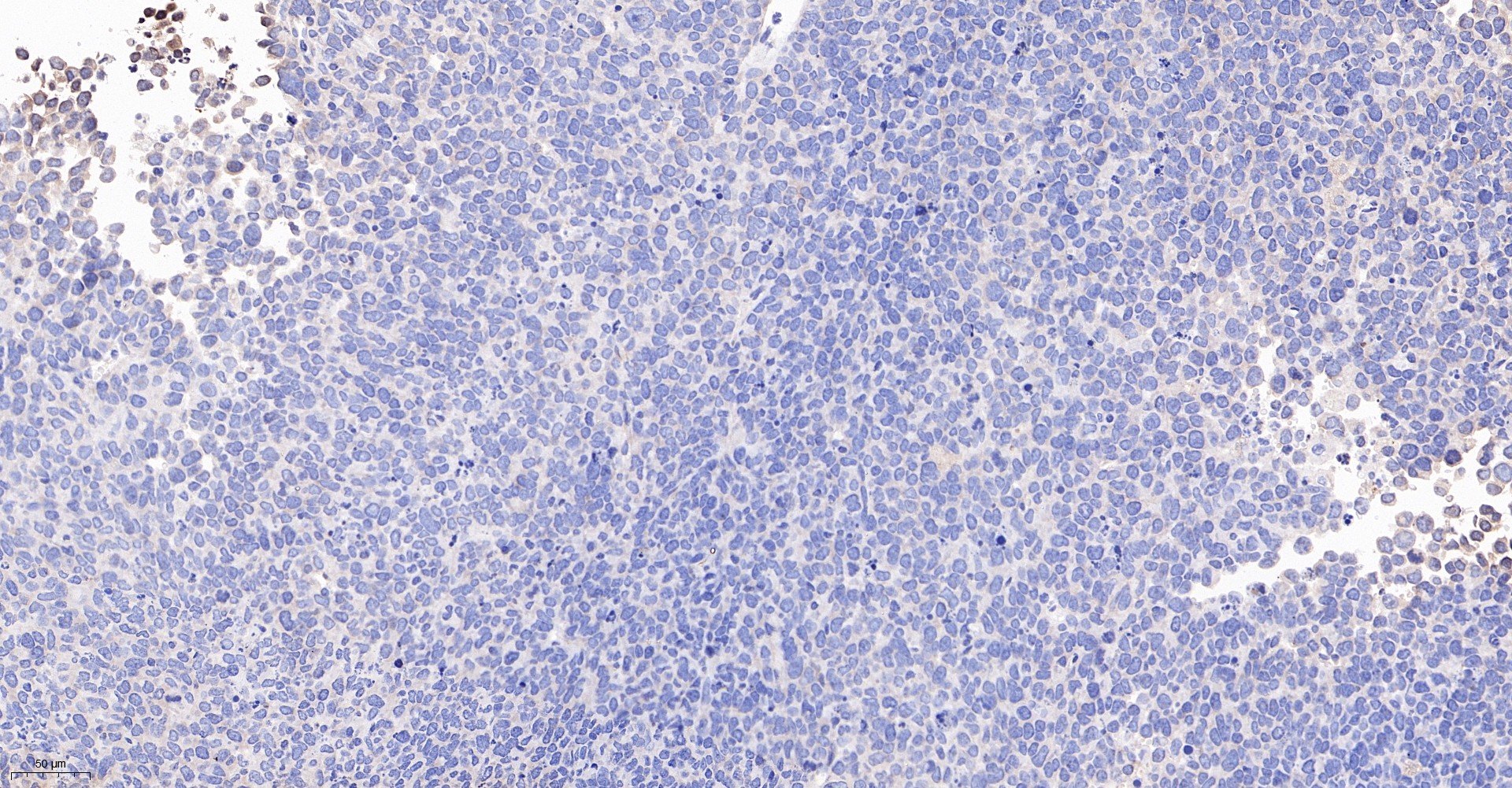

Supplement: Supplementary file 8 [file DataSheet8.zip › Expression of Bax and Bcl-2 in tumor tissue of each group/Bax/Vector BAX_20.0x.jpg]

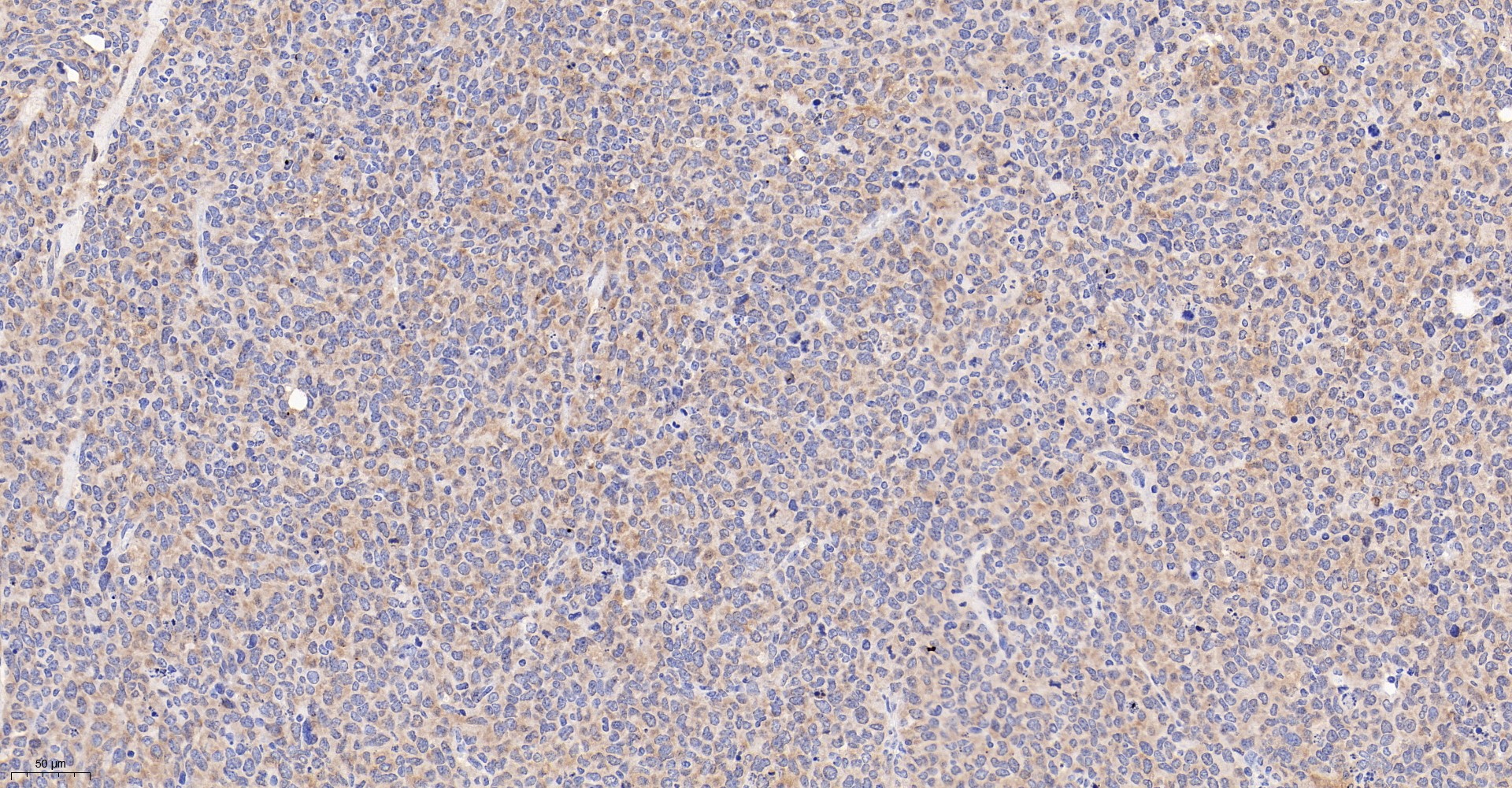

Supplement: Supplementary file 8 [file DataSheet8.zip › Expression of Bax and Bcl-2 in tumor tissue of each group/Bcl-2/IFN-γ BCL-2_20.0x.jpg]

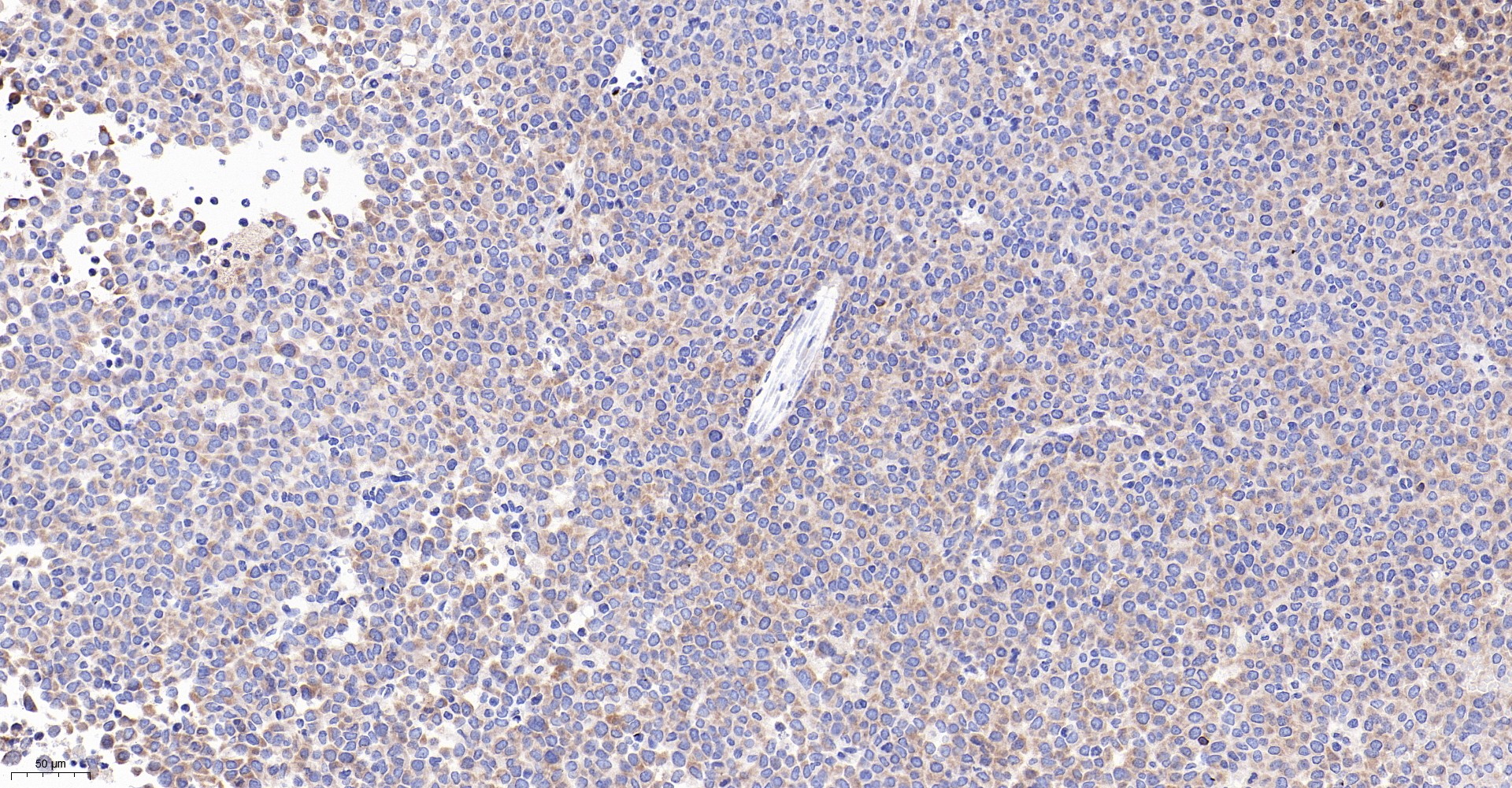

Supplement: Supplementary file 8 [file DataSheet8.zip › Expression of Bax and Bcl-2 in tumor tissue of each group/Bcl-2/IFN-γ+sPD-1 BCL-2_20.0x.jpg]

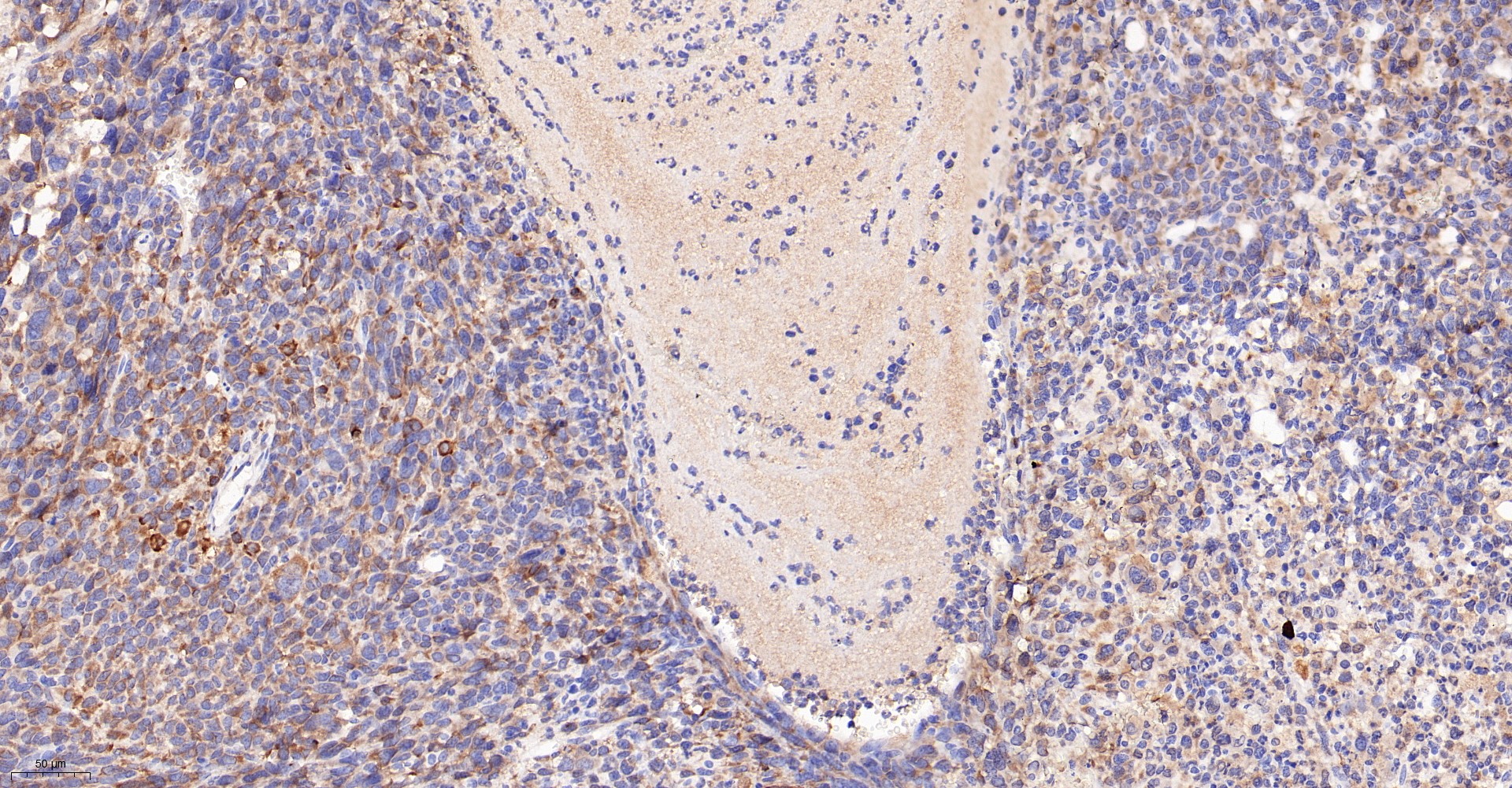

Supplement: Supplementary file 8 [file DataSheet8.zip › Expression of Bax and Bcl-2 in tumor tissue of each group/Bcl-2/Model BCL-2_20.0x.jpg]

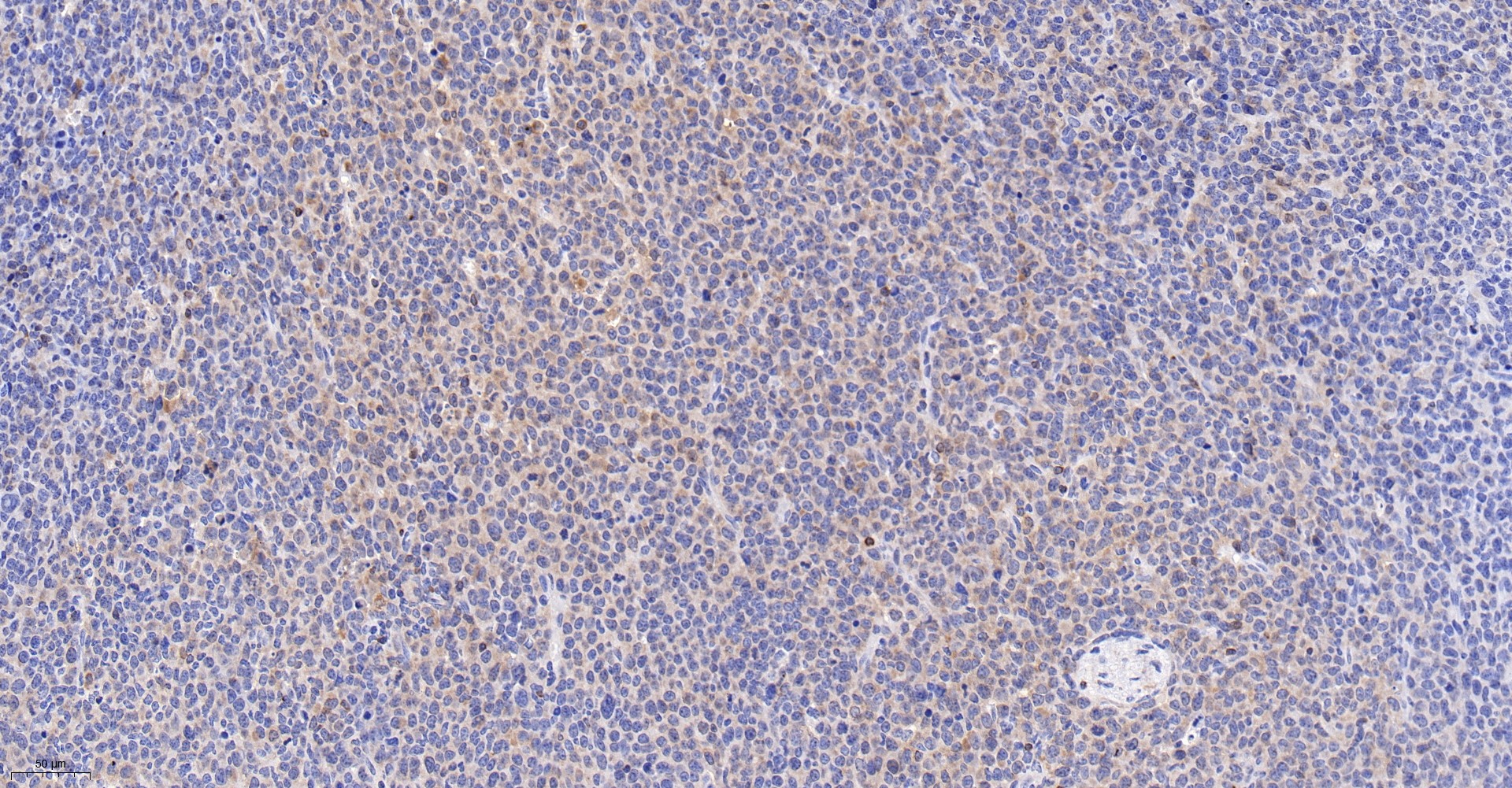

Supplement: Supplementary file 8 [file DataSheet8.zip › Expression of Bax and Bcl-2 in tumor tissue of each group/Bcl-2/sPD-1 BCL-2_20.0x.jpg]

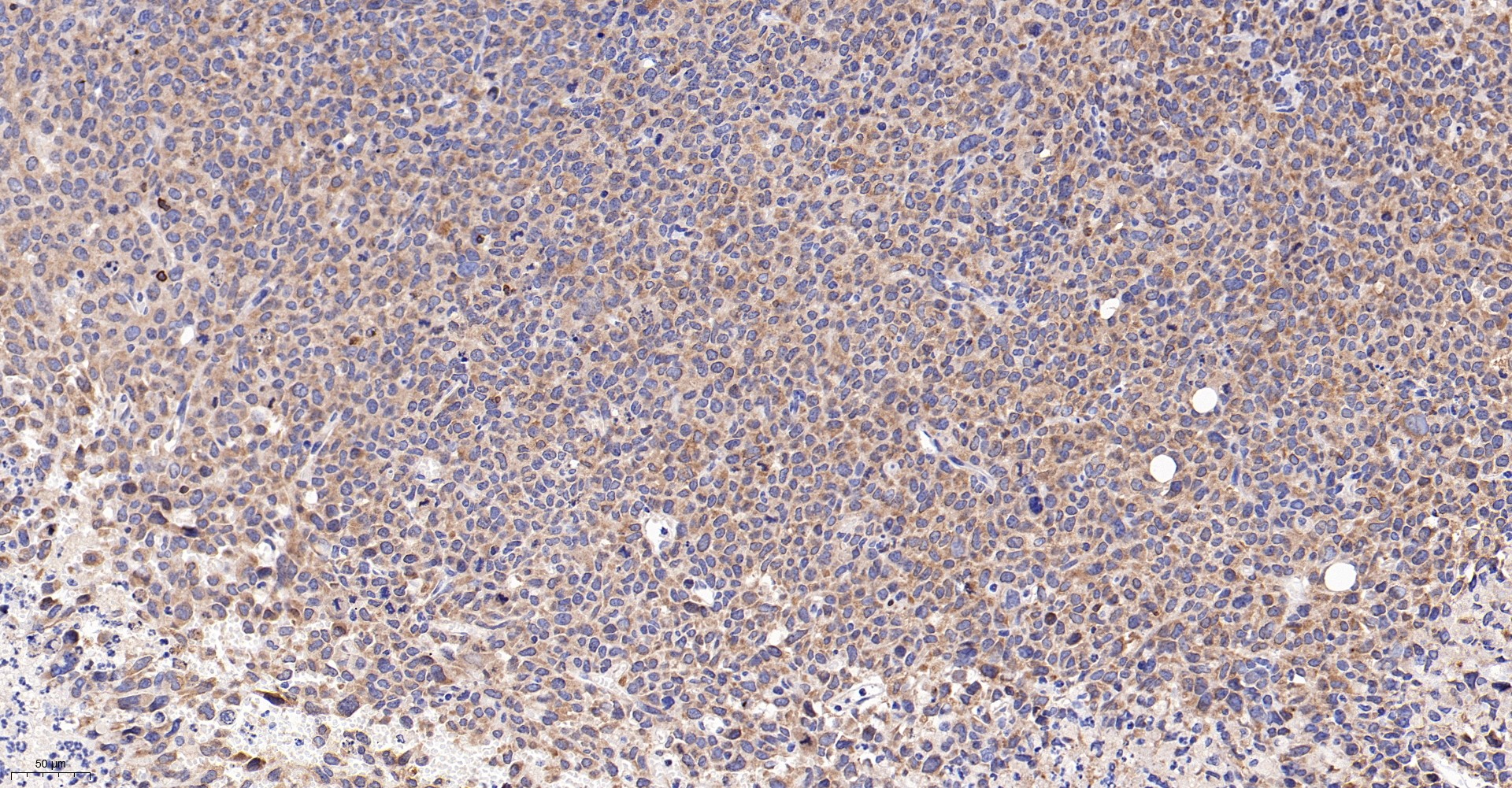

Supplement: Supplementary file 8 [file DataSheet8.zip › Expression of Bax and Bcl-2 in tumor tissue of each group/Bcl-2/Vector BCL-2_20.0x.jpg]

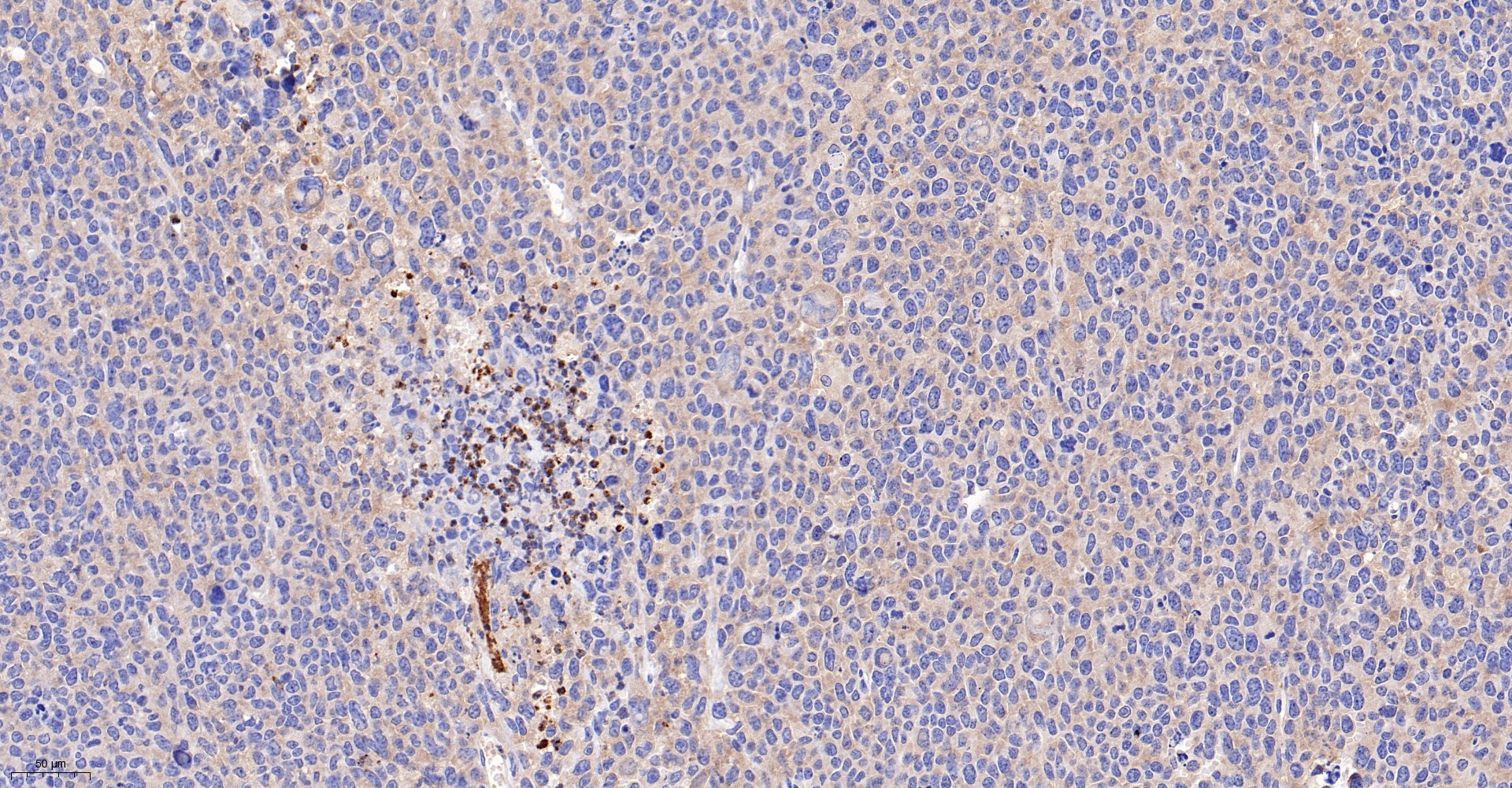

Supplement: Supplementary file 8 [file DataSheet8.zip › Expression of Bax and Bcl-2 in tumor tissue of each group/PD-L1/IFN-γ PDL1_20.0x.jpg]

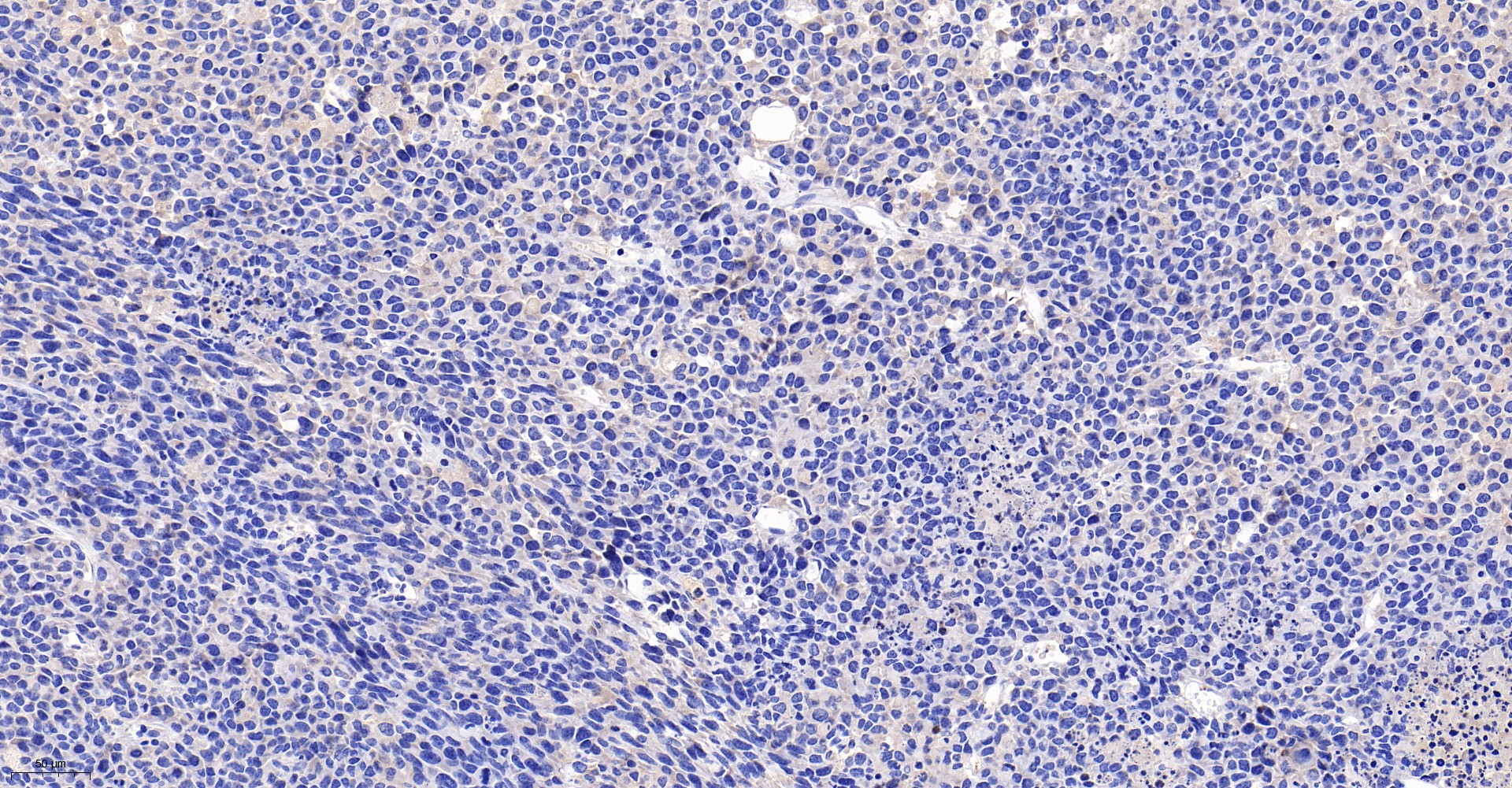

Supplement: Supplementary file 8 [file DataSheet8.zip › Expression of Bax and Bcl-2 in tumor tissue of each group/PD-L1/IFN-γ+sPD-1 PDL1_20.0x.jpg]

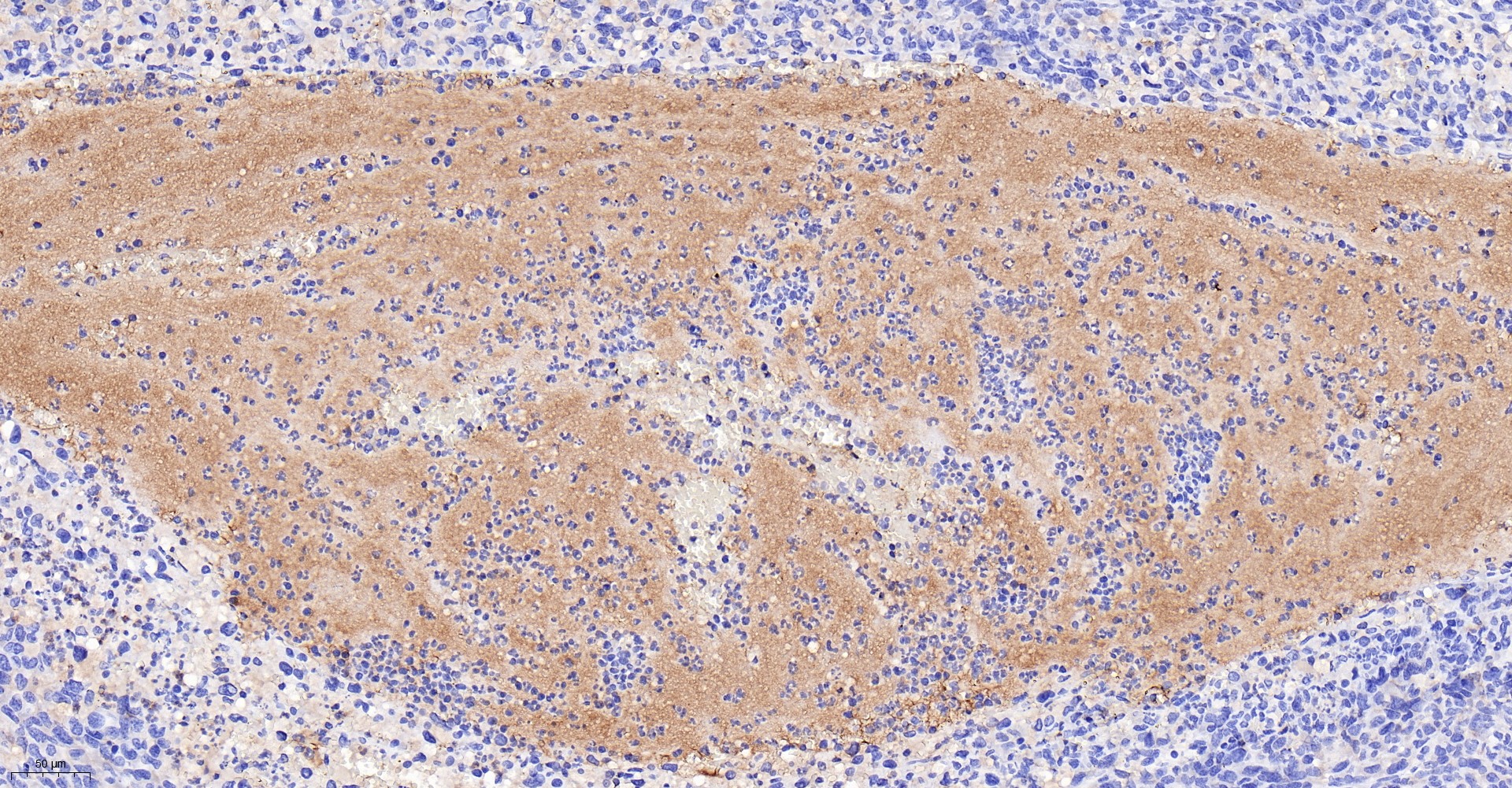

Supplement: Supplementary file 8 [file DataSheet8.zip › Expression of Bax and Bcl-2 in tumor tissue of each group/PD-L1/Model PDL1_20.0x.jpg]

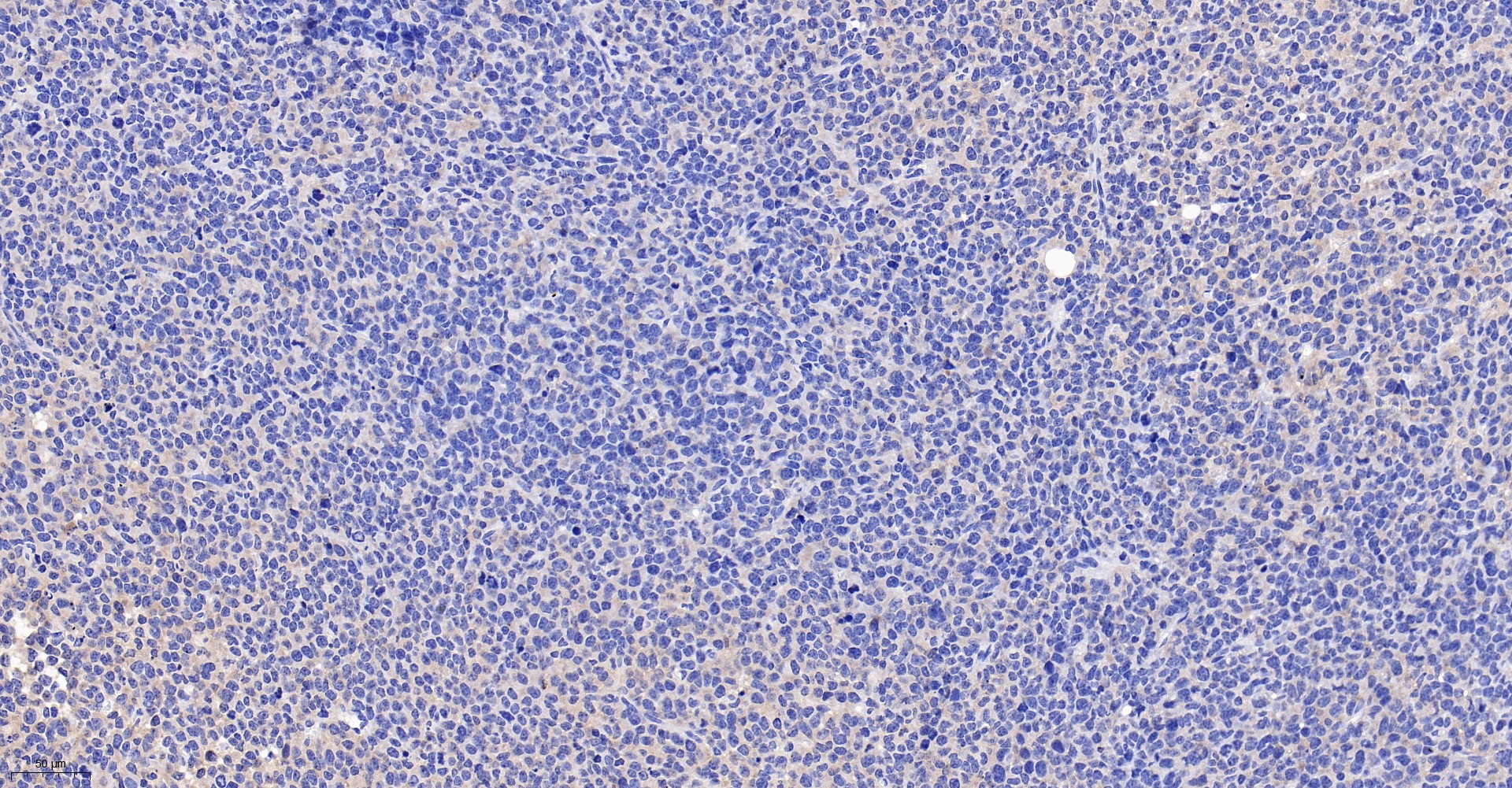

Supplement: Supplementary file 8 [file DataSheet8.zip › Expression of Bax and Bcl-2 in tumor tissue of each group/PD-L1/sPD-1 PDL1_20.0x.jpg]

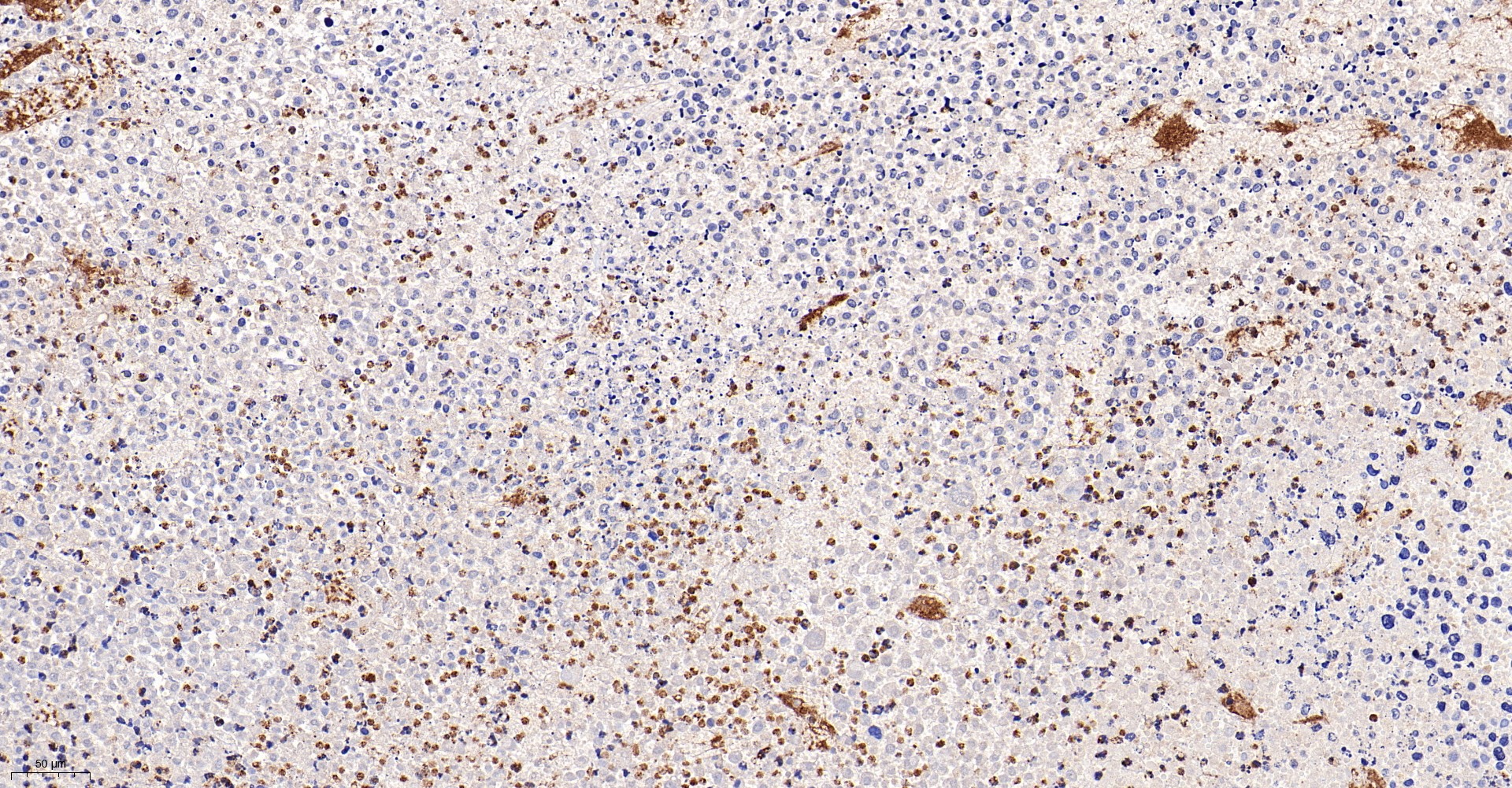

Supplement: Supplementary file 8 [file DataSheet8.zip › Expression of Bax and Bcl-2 in tumor tissue of each group/PD-L1/Vector PDL1_20.0x.jpg]

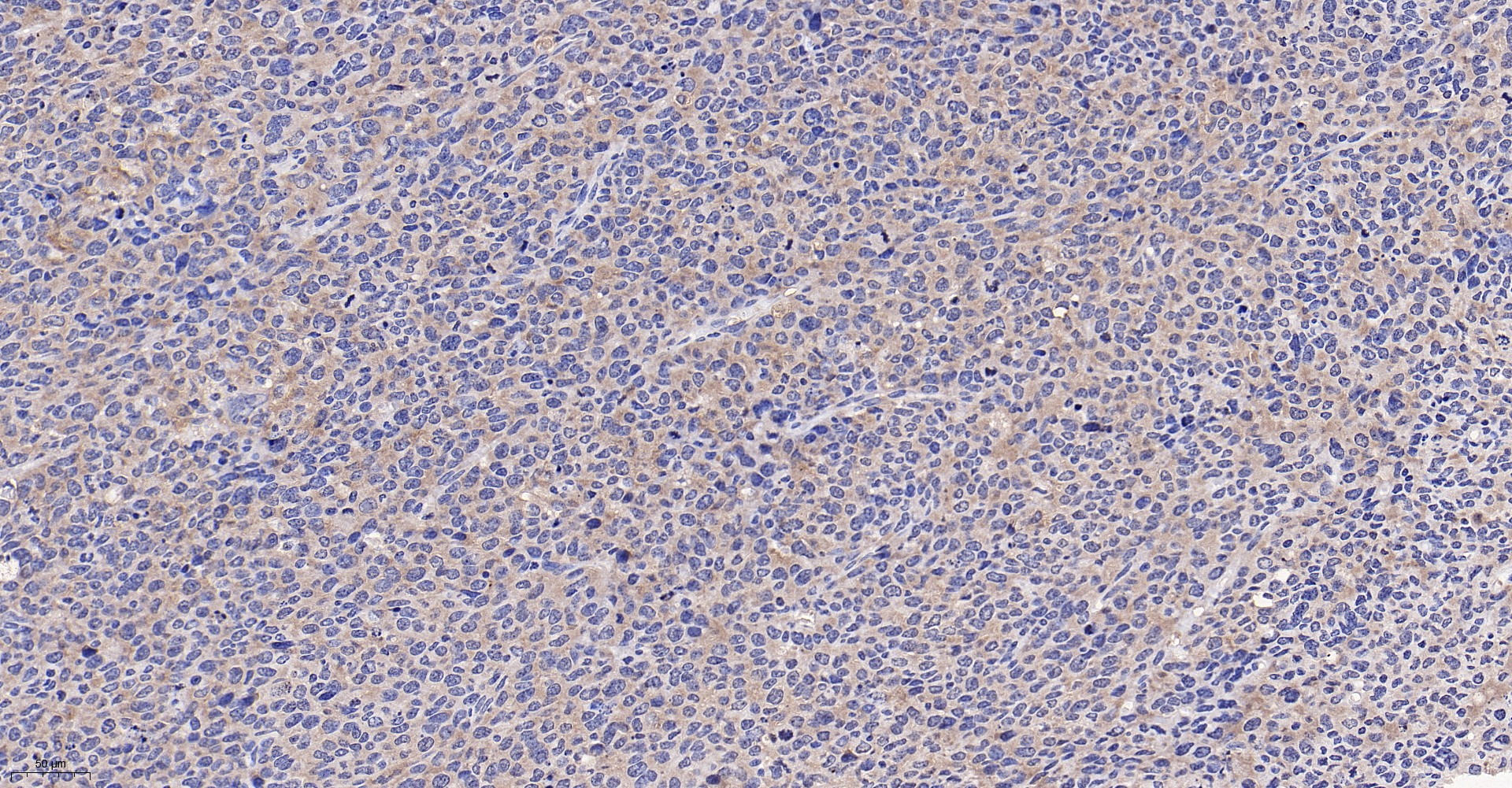

Supplement: Supplementary file 8 [file DataSheet8.zip › Expression of Bax and Bcl-2 in tumor tissue of each group/PI3K/IFN-γ PI3K_20.0x.jpg]

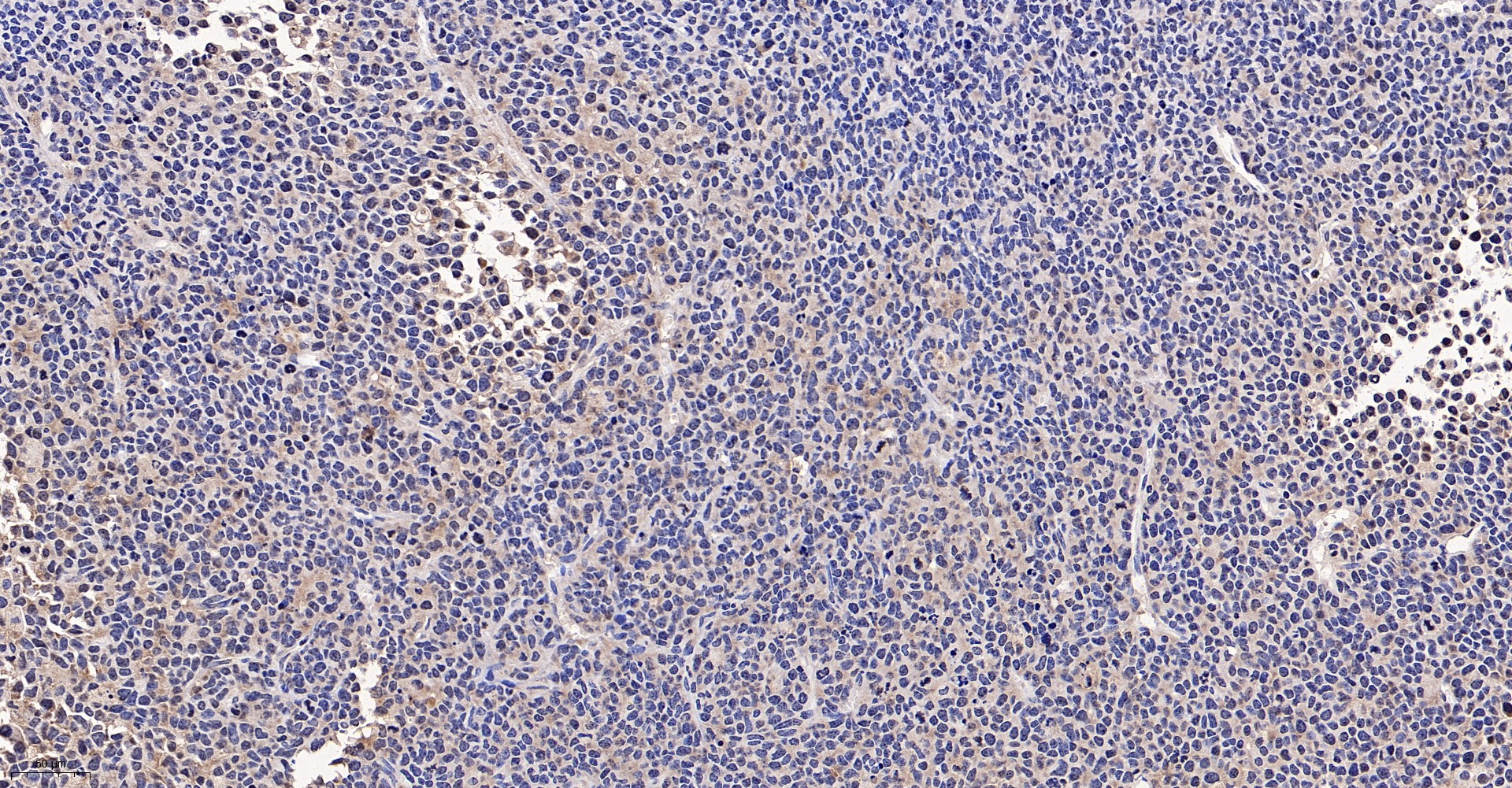

Supplement: Supplementary file 8 [file DataSheet8.zip › Expression of Bax and Bcl-2 in tumor tissue of each group/PI3K/IFN-γ+sPD-1 PI3K_20.0x.jpg]

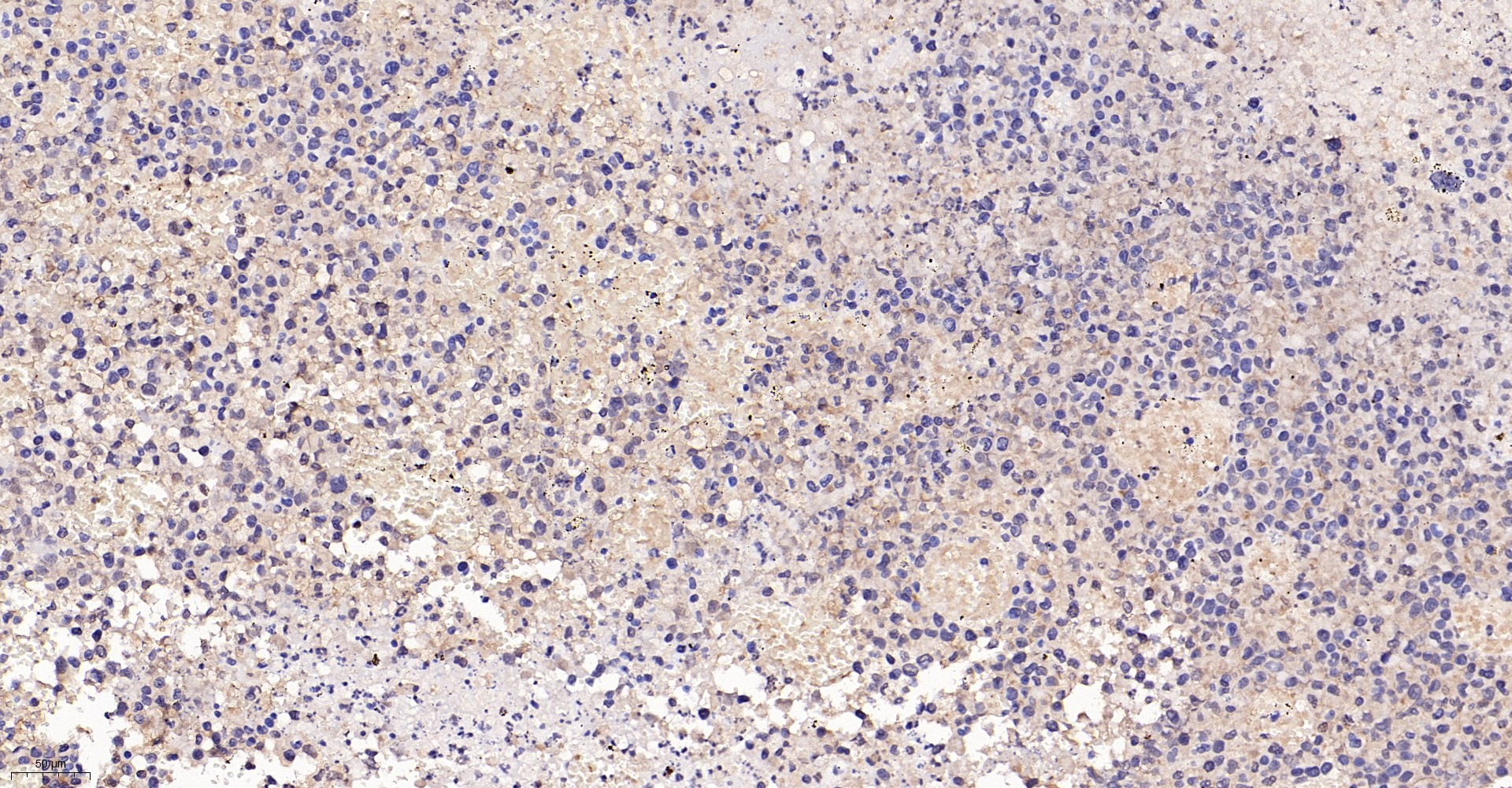

Supplement: Supplementary file 8 [file DataSheet8.zip › Expression of Bax and Bcl-2 in tumor tissue of each group/PI3K/Model PI3K_20.0x.jpg]

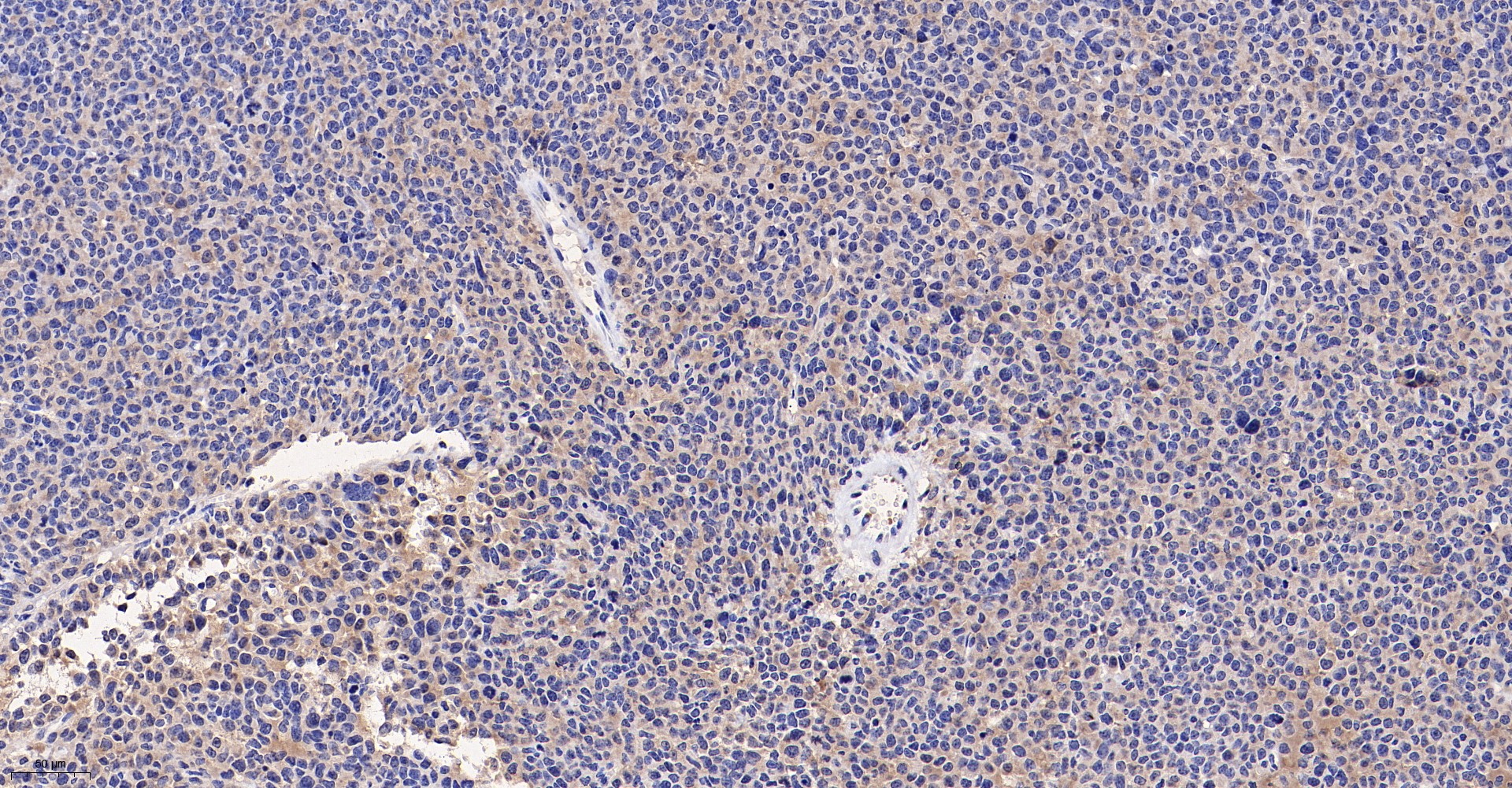

Supplement: Supplementary file 8 [file DataSheet8.zip › Expression of Bax and Bcl-2 in tumor tissue of each group/PI3K/sPD-1 PI3K_20.0x.jpg]

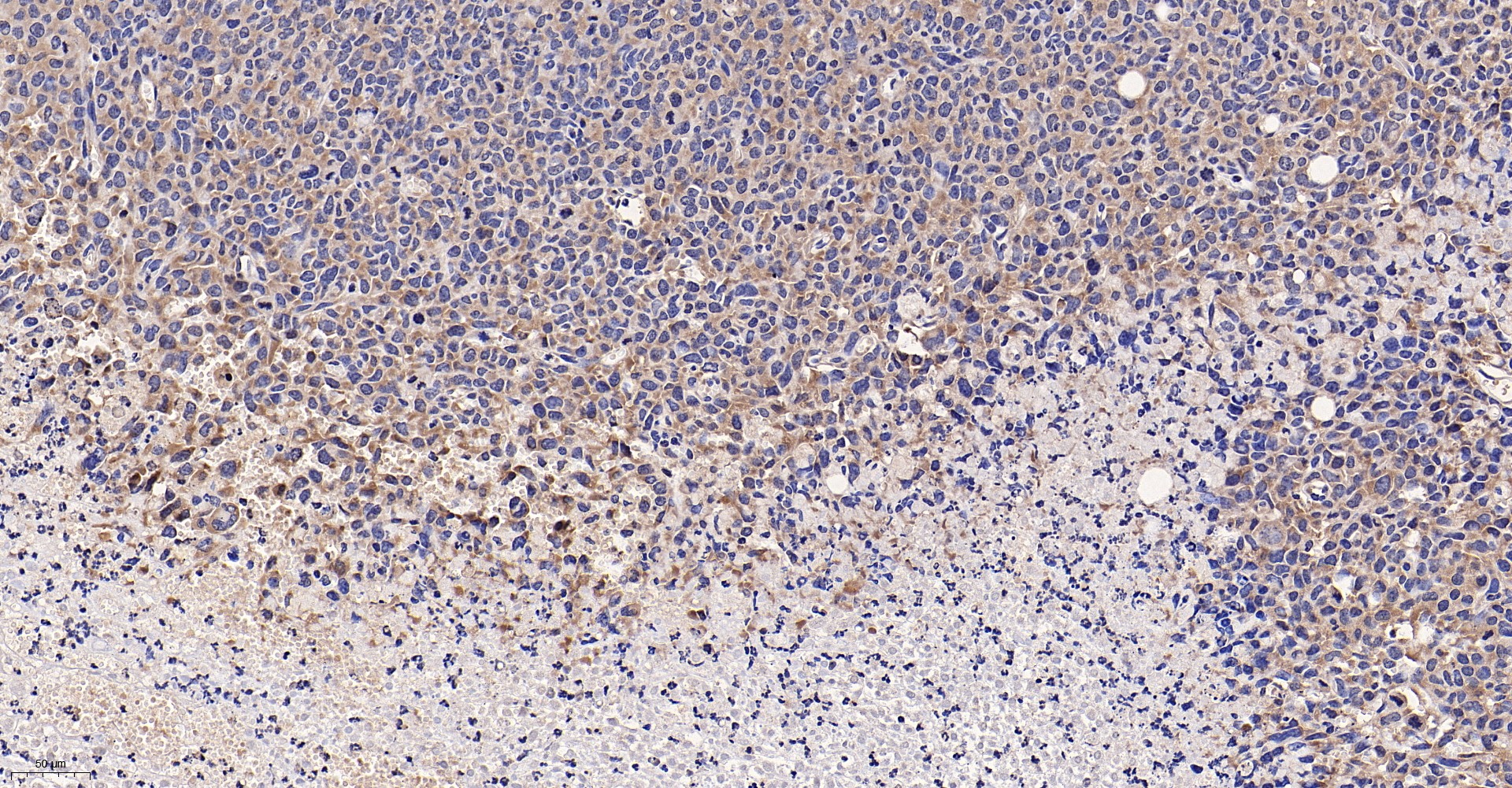

Supplement: Supplementary file 8 [file DataSheet8.zip › Expression of Bax and Bcl-2 in tumor tissue of each group/PI3K/Vector PI3K_20.0x.jpg]

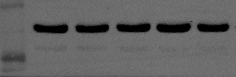

Supplement: Supplementary file 9 [file DataSheet9.zip › Original images and data of WB/Figure1D-WB/gapdh.tif]

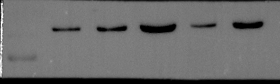

Supplement: Supplementary file 9 [file DataSheet9.zip › Original images and data of WB/Figure1D-WB/IFN-γ.tif]

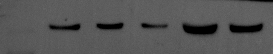

Supplement: Supplementary file 9 [file DataSheet9.zip › Original images and data of WB/Figure1D-WB/sPD-1.tif]

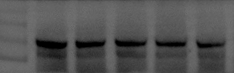

Supplement: Supplementary file 9 [file DataSheet9.zip › Original images and data of WB/Figure5C、7C 、 8C -WB/akt.tif]

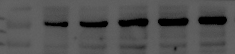

Supplement: Supplementary file 9 [file DataSheet9.zip › Original images and data of WB/Figure5C、7C 、 8C -WB/bax.tif]

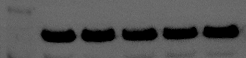

Supplement: Supplementary file 9 [file DataSheet9.zip › Original images and data of WB/Figure5C、7C 、 8C -WB/gapdh.tif]

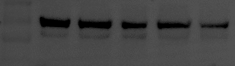

Supplement: Supplementary file 9 [file DataSheet9.zip › Original images and data of WB/Figure5C、7C 、 8C -WB/pdl1.tif]

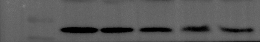

Supplement: Supplementary file 9 [file DataSheet9.zip › Original images and data of WB/Figure5C、7C 、 8C -WB/pi3k.tif]
