# Supplementary material for: Study on the efficacy of IFN-γ- and sPD-1-overexpressing BMSCs in enhancing immune effects for the treatment of lung adenocarcinoma
Source: Front Immunol. 2025 Mar 13;16:1554467. doi: 10.3389/fimmu.2025.1554467 (PMC11965897; doi:10.3389/fimmu.2025.1554467)
Supplement: Supplementary file 6 [file DataSheet6.zip › Identification of surface markers/identification of BMSCs/original data.pdf]

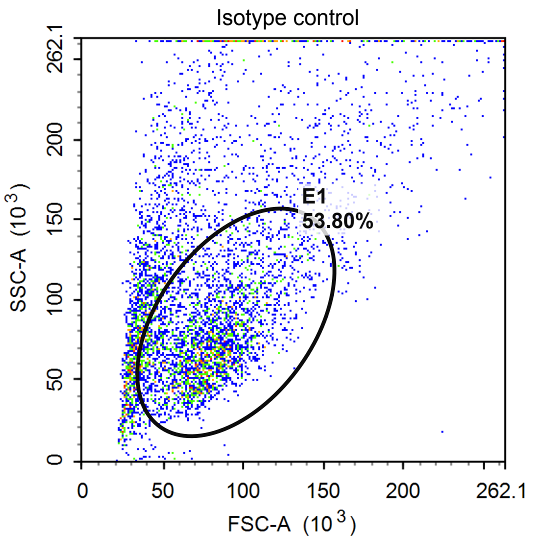
Isotype control
E1
53.80%
SSC-A (10^3)
FSC-A (10^3)
0
50
100
150
200
262.1

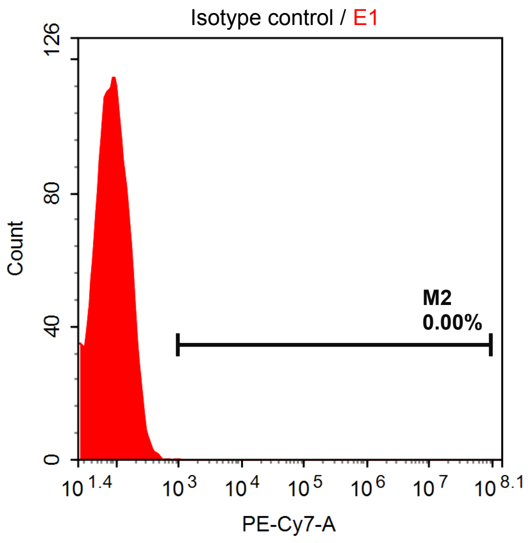
Isotype control / E1
M2
0.00%
Count
PE-Cy7-A
0
40
80
126

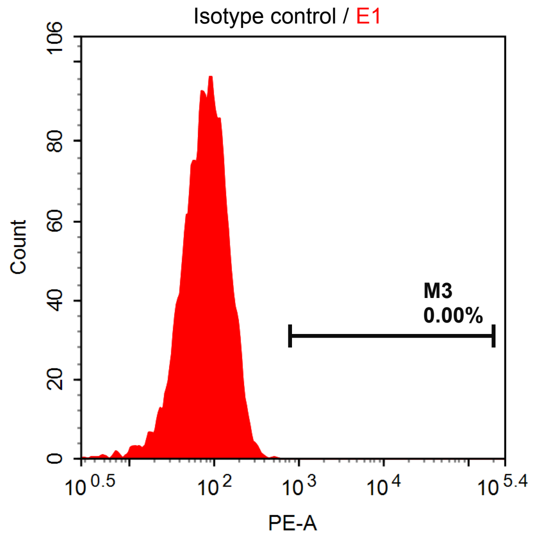
Isotype control / E1
M3
0.00%
Count
PE-A
0
20
40
60
80
106

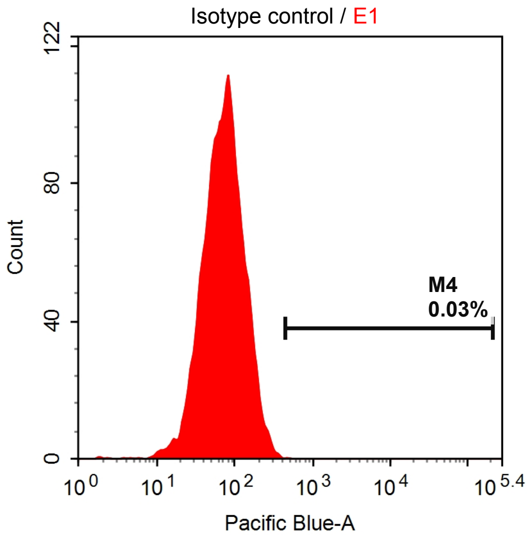
Isotype control / E1
M4
0.03%
Count
Pacific Blue-A
0
40
80
122

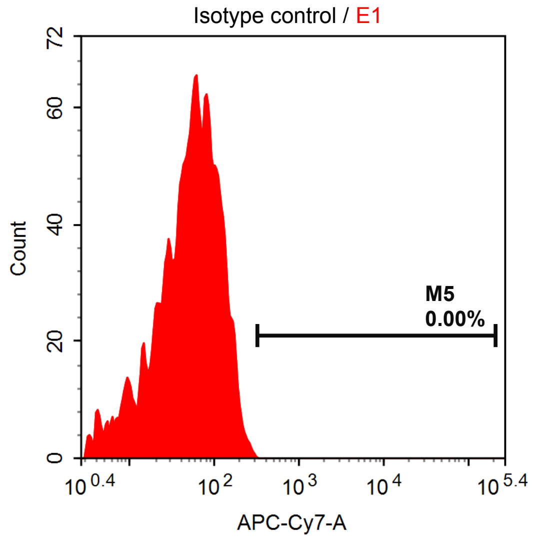
Isotype control / E1
M5
0.00%
Count
APC-Cy7-A
0
20
40
60
72

样本统计表格 - Isotype control

Gate	Color	Count	Abs. Count	% Parent	% Grandparent	% All
All	6	5,781				
E1	1	3,110	-	53.80%		53.80%
M2	2	0	-	0.00%	0.00%	0.00%
M3	3	0	-	0.00%	0.00%	0.00%
M4	4	1	-	0.03%	0.02%	0.02%
M5	5	0	-	0.00%	0.00%	0.00%

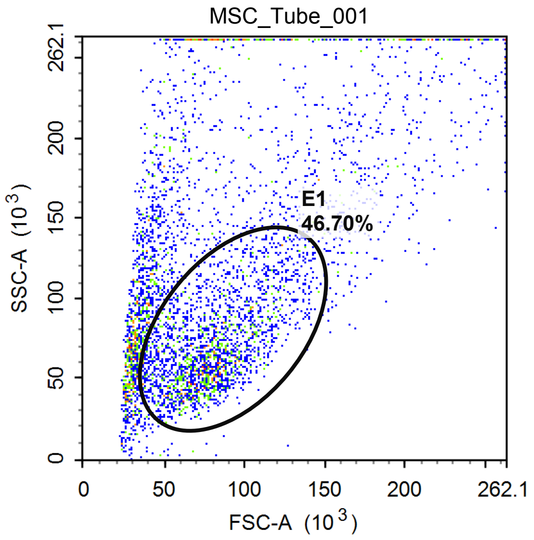

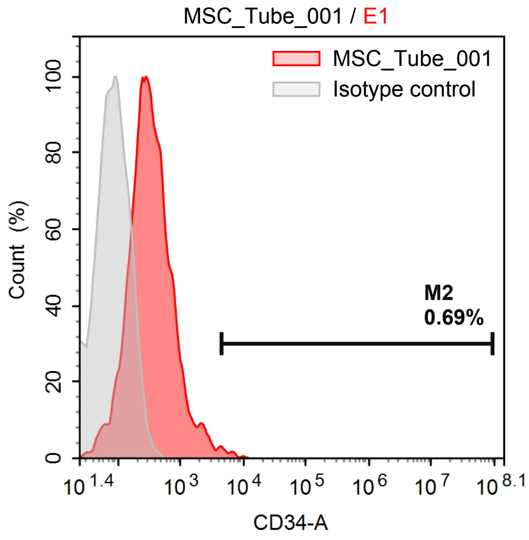

样本统计表格 - MSC_Tube_001

Gate	Color	Count	Abs. Count	% Parent	% Grandparent	% All
All	3	4,640				
└ E1	1	2,167	-	46.70%		46.70%
└ M2	2	15	-	0.69%	0.32%	0.32%

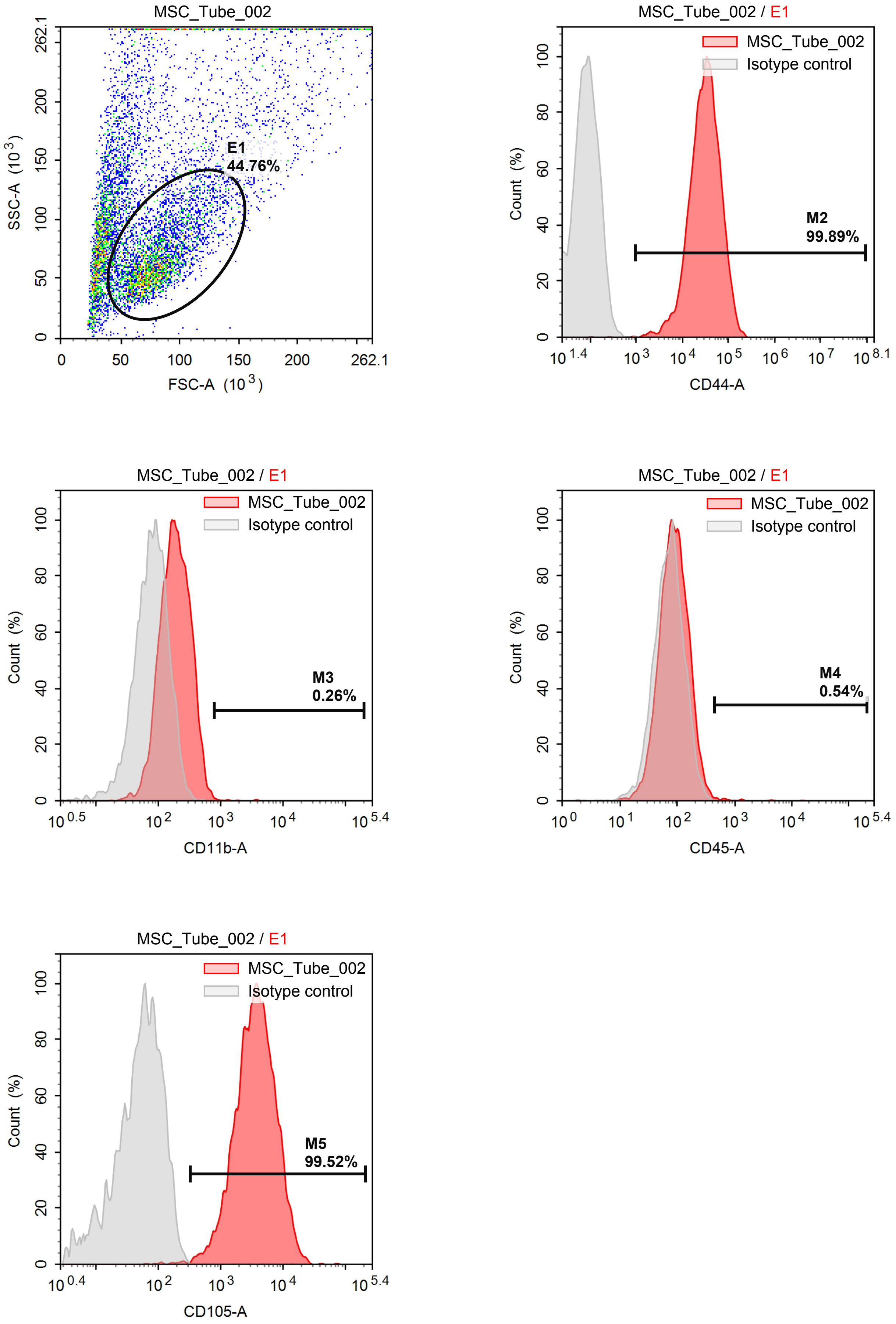

MSC_Tube_002
E1
44.76%
SSC-A (10^3)
FSC-A (10^3)
0
50
100
150
200
262.1
MSC_Tube_002 / E1
MSC_Tube_002
Isotype control
M2
99.89%
Count (%)
CD44-A
MSC_Tube_002 / E1
MSC_Tube_002
Isotype control
M3
0.26%
Count (%)
CD11b-A
MSC_Tube_002 / E1
MSC_Tube_002
Isotype control
M4
0.54%
Count (%)
CD45-A
MSC_Tube_002 / E1
MSC_Tube_002
Isotype control
M5
99.52%
Count (%)
CD105-A

样本统计表格 - MSC_Tube_002

Gate	Color	Count	Abs. Count	% Parent	% Grandparent	% All
All	6	7,844				
E1	1	3,511	-	44.76%		44.76%
M2	2	3,507	-	99.89%	44.71%	44.71%
M3	3	9	-	0.26%	0.11%	0.11%
M4	4	19	-	0.54%	0.24%	0.24%
M5	5	3,494	-	99.52%	44.54%	44.54%
